# Esterification of Aryl/Alkyl Acids Catalysed by *N*-bromosuccinimide under Mild Reaction Conditions

**DOI:** 10.3390/molecules23092235

**Published:** 2018-09-02

**Authors:** Klara Čebular, Bojan Đ. Božić, Stojan Stavber

**Affiliations:** 1 Centre of Excellence for Integrated Approaches in Chemistry and Biology of Proteins, Jamova 39, 1000 Ljubljana, Slovenia; klara.cebular@ijs.si; 2 Jožef Stefan International Postgraduate School, Jamova 39, 1000 Ljubljana, Slovenia; 3 Department of Physical and Organic Chemistry, Jožef Stefan Institute, Jamova 39, 1000 Ljubljana, Slovenia; 4 Institute of Physiology and Biochemistry, Faculty of Biology, University of Belgrade, Studentski trg 16, 11000 Belgrade, Serbia; bbozic@bio.bg.ac.rs

**Keywords:** esterification, *N*-halosuccinimide, metal-free catalyst, aryl acids, alkyl acids

## Abstract

*N*-halosuccinimides (NXSs) are well-known to be convenient, easily manipulable and low-priced halogenation reagents in organic synthesis. In the present work, *N*-bromosuccinimide (NBS) has been promoted as the most efficient and selective catalyst among the NXSs in the reaction of direct esterification of aryl and alkyl carboxylic acids. Comprehensive esterification of substituted benzoic acids, mono-, di- and tri-carboxy alkyl derivatives has been performed under neat reaction conditions. The method is metal-free, air- and moisture-tolerant, allowing for a simple synthetic and isolation procedure as well as the large-scale synthesis of aromatic and alkyl esters with yields up to 100%. Protocol for the recycling of the catalyst has been proposed.

## 1. Introduction

Esterification reaction is one of the most important synthetic routes in organic synthesis due to the significance of its products. It is an irreplaceable reaction step during the synthesis of pharmaceuticals, cosmetics, plasticizers, perfumes, flavour chemicals, fine chemicals, electronic materials, solvents and chiral auxiliaries [1] and together with transesterification, it is a transformation of major significance in the biodiesel production [2,3,4,5]. Aside from being among the most prevalent final products and/or intermediates in the fields of science and industry, esters also play a significant role in biology, as the ester bonds are key linking groups in many primary lipid metabolites as well as secondary cyclodepsipeptide and polyketide metabolites [6]. As a result, a plethora of approaches have been reported for ester preparation, one of the most common being the reaction of direct esterification between carboxylic acids and alcohols (Fischer esterification) [7]. Conventionally, it is performed with excessive amounts of reagents/dehydrating agents or with activated carboxylic acid derivatives in the presence of a stoichiometric base, which results in significant amounts of by products and waste at the end of the process as well as in energy-, time- and solvent-consuming purification [8]. Therefore, methods of catalytic direct condensation between carboxylic acids and alcohols, which lack these disadvantages, have recently become an attractive research subject, employing a broad spectra of different catalysts, such as Brønsted acids [9], metal catalysts [10,11], Lewis acids [12,13], solid-supported catalysts [14,15] and solid acids [16,17], ionic liquids [18,19], PPh_3_-based catalysts [20], enzymes [21,22], zeolites [23,24], etc. From the industrial and sustainability perspective, the ideal esterification method would include the use of easily manipulable, metal-free, low-cost, water- and air-tolerant recyclable catalyst and mild solvent-free reaction conditions without the need for stoichiometric amounts of activators, large excesses of reagents and simultaneous removal of water. It should also be applicable to a broad substrate scope with high selectivity; it should be suitable for large-scale synthesis and allow a simple purification procedure, providing high product yields. The majority of known methods do not comply with at least one of the mentioned criteria; therefore, the field of sustainable design of esterification methods remains an attractive research challenge.

*N*-halosuccinimides (NXSs) belong to the class of *N*-halo reagents which are widely used in organic synthesis as halogenating, hydroxyhalogenating, oxidizing and condensing agents [25]. Their reactivity originates from the great lability of the N–X bond and various modes of its splitting [26]. Depending on the reaction conditions, different highly reactive species can be formed: *N*-radicals, *N*-cations, *N*-anions as well as their corresponding halogen counter particles, etc. [27]. Due to these convenient chemical properties, together with their metal-free character, low-cost, accessibility and higher stability relative to other *N*-halo reagents, NXSs have recently been attracting attention as mediators in substoichiometric amounts for different types of organic transformations [28,29,30,31]. The catalytic potential of *N*-bromosuccinimide (NBS) has recently been reviewed [32]; however, to the best of our knowledge, the use of *N*-bromosuccinimide as the only catalytic component in substoichiometric amounts for direct dehydrative esterification between alcohols and carboxylic acids has not been explored so far.

**Scheme 1:**
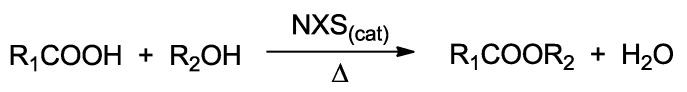
Direct dehydrative esterification of carboxylic acids catalysed by substoichiometric amounts of NXSs.

In our continuous pursuit of sustainable synthetic protocols [33,34,35,36,37], including those where NXSs are used as reagents [38,39,40,41,42,43,44,45], we now report and introduce the use of substoichiometric amounts of these compounds as mediators for efficient and selective comprehensive metal-free direct esterification of carboxyl functionality in organic molecules under neat reaction conditions (Scheme 1).

## 2. Results and Discussion

Previously, some advantages of *N*-bromosuccinimide (NBS) as a catalyst in reactions of transesterification have been observed, but the reaction was limited to transesterification of α-keto esters [46] or acetylation of alcohols using acetic anhydride [47]. In addition, esterification of carboxylic acids with alcohols in the presence of triphenylphosphine (PPh_3_) and *N*-bromo/iodosuccinimides has been reported as a method for ester preparation [48]. However, its significant drawback is posed by the by-product, phosphine oxide, formed in equimolar amounts, as it is difficult to remove during the purification step. Besides, the esterification was performed in halogenated solvent (dichloromethane) in the presence of equimolar amounts of base (pyridine) and NBS/NIS. Furthermore, molecular iodine has been presented as a convenient Lewis acid catalyst for direct dehydrative esterification, but attempts to prepare esters from corresponding aromatic acids have been found unsuccessful [49] or unselective [50,51]. Furthermore, bromine (Br_2_)-mediated esterification of carbocyclic acids with methanol has been reported [52], though this methodology carries handling safety risks due to the potentially hazardous effects of Br_2_/MeOH solution [53]. All the aforementioned provided us with an impetus to improve the reaction of direct esterification of aryl/alkyl acids. Since *N*-halosuccinimides have been widely used as more convenient and safer X_2_ substitutes in halogenation reactions, the catalytic activity of corresponding *N*-halosuccinimide derivatives in the reaction of direct condensation between various structurally different carboxylic acid and alcohols has been investigated and presented herein.

Initially, benzoic acid (**1**) has been chosen as an aromatic acid model molecule to verify expectations and optimize reaction protocols. In the typical experimental procedure, **1** has been refluxed with methanol (MeOH) in the presence of substoichiometric amounts of NXS: *N*-chlorosuccinimide (NCS), NBS, or *N*-iodosuccinimide (NIS). The results presented in Table 1 reveal that NBS seems to be the most promising catalyst in reactions of esterification, while without the presence of any of the NXSs, no conversion of starting material has been observed. In search of optimal reaction conditions, the effect of catalyst NBS loading has been examined by changing the amount of catalyst from 3 mol% to 15 mol% (Table 1). The amount below 7 mol% NBS furnished significantly lower conversion, while increasing the amount above 7 mol% did not significantly improve the yield of the corresponding ester. Therefore, further studies were carried out with 7 mol% of NBS. Moreover, the influence of temperature on the conversion of **1** to methyl benzoate (**1a**, Table 1) has been studied. The variation of temperature from 30 °C to 100 °C has been found to have a significant impact on the conversion of the acid to ester with the optimal temperature being 70 °C. Moreover, under dry reaction conditions (in the presence of Na_2_SO_4_) the efficiency of the esterification dropped considerably (entry 8). The promoting activities of NXSs, halogen mineral acids and molecular bromine were compared in the present esterification reaction. In the case of both aqueous HCl (entry 12) and molecular Br_2_ (entry 15), the conversions were comparable and their activity was similar to that of NBS (entry 6). On the other hand, the esterification efficiency in the case of both HBr and HI was considerably lower (entry 13 and 14) and resulted in the conversion yields of 84% and 68%, respectively. From the green-chemical point of view, NBS exhibited the highest catalytic activity among the examined catalysts.

**Figure 1:**
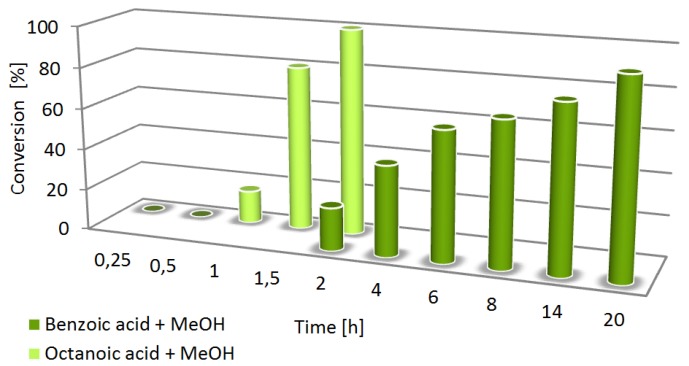
The effect of reaction time on the efficiency of esterification of benzoic (**1**) and octanoic acid (**2**) with methanol in the presence of 7 mol% of NBS at 70 °C.

The optimal reaction conditions presented above (entry 6, Table 1) have been applied to 1-octanoic acid (**2**) as an alkyl acid model compound. As expected, 1-octanoic acid has been quantitatively converted to the corresponding methyl octanoate (**2a**, Figure 1), while in the absence of NBS, no conversion of the starting material was observed.

To compare the reactivity of the aromatic and alkyl acids, the optimization of reaction time has been performed (Figure 1). Relative to benzoic acid, significantly higher activity of alkyl acid (**2**) has been observed, which was in accordance with our expectations. Due to resonance stabilization of carboxyl group in aromatic acids, its lower activation resulted in longer reaction time and slightly lower conversion. 

Encouraged by these promising results, the scope of the alcohols, suitable for esterification with acids **1** and **2**, has been studied (Table 2). It can be observed that almost in all cases, alkyl acid (**2**) is significantly more active than aromatic acid (**1**) and the conversions to the corresponding esters (**2a**–**i**) are higher. Since nucleophilic characters, as well as steric properties, have been envisioned to have a considerable impact on reaction kinetics, different alcohols have been tested (**a**–**i**, Table 2). 2-Fluoro-1-ethanol (FCH_2_CH_2_OH, **b**) is a significantly weaker nucleophile than MeOH, therefore the reaction time for esterification of **1** as well as of **2** had to be prolonged. The elongation of the alcohol alkyl chain (**a**, **d**, **f**) had a stronger impact on esterification efficiency of benzoic acid than on esterification efficiency of octanoic acid, resulting in higher yields of octanoate esters (**2a**, **2d**, **2f**) relative to benzoate esters (**1a**, **1d**, **1f**). Moreover, the effect of increased steric hindrance of the nucleophilic alcohol component was followed by varying the bulkiness of alcohol from primary to tertiary structure (**a**, **c** and **g**, **e** and **i**). Interestingly, the esterification of octanoic acid was considerably less affected by the structural change from primary (MeOH, **2a**) to secondary alcohol (*i*-PrOH and cyclopentanol, **2c** and **2g**) than the transformation of benzoic acid, where low (*i*-PrOH, **1a**) or no conversion (cyclopentanol, **1g**) was detected. Unfortunately, the limitation of the method was observed in reactions with bulky tertiary alcohols *t*-BuOH (**e**) and adamantanol (**i**), where no product was detected. Similarly, when phenol (**h**) has been used as the nucleophile, no reaction products were noticed, which can be assigned to the low nucleophilicity originating from the relatively high acidity of phenol molecule.

Due to the commercial importance of certain methyl esters in biodiesel production (fatty acid methyl esters, FAME) and perfumery (methyl benzoate), esterification of different types of carbocyclic acids with MeOH under optimal reaction conditions has been furtherly investigated (Table 3). As can be noticed, electronic effects of substituents, as well as their position on the phenyl ring of the investigated aromatic acids have exhibited a significant influence on the conversion of acids (**3**–**12**) to their corresponding methyl esters. 

Strong electron-withdrawing (4-NO_2_, 3-NO_2_), as well as weak electron-donating groups (4-Me, 3-Me) with only positive inductive +I and no mesomeric effect (M) have expressed moderate impact on the yield, while substituents with positive mesomeric (+M) and negative inductive (–I) effects (4-F, 3-F) have resulted in a decrease in the conversion. The strong electron-donating group at para position (–OMe, compound **9**) has completely inhibited the reaction due to the strong resonance interaction between the lone electron pair of the substituent and carboxyl functional group, while *m*–positioned methoxy group which could not resonate with carboxyl moiety, resulted in fair yield of target benzoate **10a**. On the other hand, in the case of aliphatic carboxylic acids, no scope limits have been observed and methyl stearate (**13a**), as well as methyl oleate (**14a**), methyl 2-cyanoacetate (**20a**), (*S*)-methyl 2-acetamido-3-phenylpropanoate (**21a**), methyl 2-(1*H*-indol-3-yl)acetate (**22a**) and methyl 4-(4-chlorophenyl)-4-oxobutanoate (**23a**) have been obtained almost quantitatively, while dimethyl oxalate (**15a**), trimethyl citrate (**16a**), and adamantane-1-carboxylic acid methyl ester (**17a**) have been gained in excellent yields.

Furthermore, the applicability of the method on more complex structural backbones has been demonstrated by performing the esterification of two significant steroidal carboxylic acids: cholic acid as one of the most common bile acids, formed as an end product of cholesterol metabolism in the liver [54], and its derivative dehydrocholic acid, which is the main component in many drugs against cholestatic liver disease and for dissolution of cholesterol gallstones [55]. In both cases, an excellent conversion of the starting material was achieved already after 1 h. Although in the crude reaction mixture obtained after esterification of dehydrocholic acid, partial conversion to ketal was observed, it was quantitatively converted into the corresponding ester during the isolation step by washing the mixture with 10% HCl _(aq)_.

Moreover, to confirm the synthetic value of the presented methodology, synthesis of methyl benzoate (**1a**), methyl stearate (**13a**) and methyl citrate (**16a**) has been performed on 10–40 mmol scale with high to excellent yields (85–100%).

## 3. Materials and Methods 

### 3.1. General Information

All reactions were performed in Mettler-Toledo Easymax 102 Advanced Synthesis Workstation using 25 mL reactor tubes. NMR spectra were recorded on Varian Inova 300 spectrometer (300 MHz ^1^H, 75 MHz ^13^C, 285 MHz ^19^F) at 25 °C. ^1^H-NMR spectra were obtained as solutions in CDCl_3_ with TMS as the internal standard. ^19^F-NMR spectra were obtained as solutions in CDCl_3_ with CFCl_3_ as the internal standard. *N*-bromosuccinimide was freshly recrystallized before use. All other chemicals used for synthetic procedures were obtained from commercial sources and were of reagent grade purity or better (Merck, Sigma Aldrich, Carlo Erba, Fluka, Fisher Scientific, Apollo Scientific, etc.). Reactions were monitored by TLC with silica gel coated plates Silica gel/TLC cards, DC-Alufolien-Kieselgel with 60 Å medium pore diameter (Sigma Aldrich) and detection was conducted by UV absorption (254 nm). Purification of certain products was conducted on preparative silica gel glass plates PLC Kieselgel 60 F254 with 2 mm layer thickness. Succinimide, isolated at the end of the reaction, can easily be recycled back to *N*-bromosuccinimide according to the standard procedure by NaOH, as elaborated in other reports [56]. Copies of ^1^H-NMR, ^13^C-NMR and ^19^F-NMR spectra of isolated final products are available in Supplementary material file online.

### 3.2. Experimental Procedures

#### 3.2.1. General Procedure for the Esterification between Carboxylic Acids and Alcohols

The mixture of carboxylic acid, alcohol and *N*-bromosuccinimide was stirred in a 25 mL reactor tube at 70 °C for 2–40 h. After the completion of the reaction, the mixture was cooled to room temperature and alcohol was evaporated under reduced pressure. The isolation procedure was as follows, except where noted differently in Section 3.2.6. The residue was dissolved in ethyl acetate and consecutively washed with 10 mL of 10% Na_2_S_2_O_3 (aq)_, 5 mL of saturated NaHCO_3 (aq)_ and 10 mL of distilled water. The water phase was extracted with ethyl acetate (3 × 5 mL). The organic layers were combined, dried over Na_2_SO_4_ and the solvent was evaporated under reduced pressure.

#### 3.2.2. Scale-Up Procedure for Preparation of Methyl Benzoate (**1a**) and Isolation of Succinimide

The mixture of benzoic acid (40 mmol, 4.88 g), MeOH (20 mL) and *N*-bromosuccinimide (2.80 mmol, 0.50 g) was stirred in a 25 mL reactor tube at 70 °C for 20 h. After the completion of the reaction, the mixture was cooled to room temperature and alcohol was evaporated under reduced pressure. The residue was washed with distilled water (20 mL) and the water phase was extracted with ethyl acetate (2 × 20 mL). The organic layers were combined and washed with the mixture of 10 mL of saturated NaHCO_3 (aq)_, 10 mL of 10% Na_2_S_2_O_3 (aq)_ and 15 mL of distilled water. The water layer was again extracted with ethyl acetate (2 × 20 mL). The organic layers were combined, dried over Na_2_SO_4_ and the solvent was evaporated under reduced pressure to furnish methyl benzoate as colourless oil. The water layer from the first washing of the crude reaction mixture was evaporated under the reduced pressure to give succinimide as a white solid.

Yield (methyl benzoate): 4.60 g, 85%.
Yield (succinimide [57]): 272 mg, 98%.
^1^H NMR (300 MHz, CDCl_3_) δ 10.04 (s, 1H), 2.72 (s, 4H).
^13^C NMR (76 MHz, CDCl_3_) δ 178.8, 29.4.

#### 3.2.3. Scale-Up Procedure for Preparation of Trimethyl Citrate (**16a**)

The mixture of citric acid (11 mmol, 2.11 g), MeOH (20 mL) and *N*-bromosuccinimide (0.77 mmol, 0.138 g) was stirred in a 25 mL reactor tube at 70 °C for 20 h. After the completion of the reaction, the mixture was cooled to room temperature and alcohol was evaporated under reduced pressure. The residue was dissolved in 50 mL of ethyl acetate, washed with the mixture of 10 mL of saturated NaHCO_3 (aq)_, 10 mL of 10% Na_2_S_2_O_3 (aq)_ and 25 mL of distilled water and the water phase was extracted with ethyl acetate (2 × 25 mL). The organic layers were combined, dried with Na_2_SO_4_ and the solvent was evaporated under reduced pressure to furnish methyl citrate as a white solid.

Yield: 2.55 g, 99%.

#### 3.2.4. Scale-Up Procedure for Preparation of Methyl Stearate (**13a**)

The mixture of stearic acid (10 mmol, 2.85 g), MeOH (20 mL) and *N*-bromosuccinimide (0.70 mmol, 0.125 g) was stirred in a 25 mL reactor tube at 70 °C for 20 h. After the completion of the reaction, the mixture was cooled to room temperature and alcohol was evaporated under reduced pressure. The residue was dissolved in 50 mL of ethyl acetate, washed with the mixture of 10 mL of saturated NaHCO_3 (aq)_, 10 mL of 10% Na_2_S_2_O_3 (aq)_ and 15 mL of distilled water and the water phase was extracted with ethyl acetate (2 × 25 mL). The organic layers were combined, washed with distilled water (2 × 20 mL), dried with Na_2_SO_4_ and the solvent was evaporated under reduced pressure to furnish methyl stearate as a white solid.

Yield: 2.99 g, 100%.

#### 3.2.5. Procedure for Recycling of *N*-bromosuccinimide (NBS) from Waste Succinimide

Succinimide (0.272 g, 2.75 mmol) was dissolved in a mixture of 1.36 g (3.29 mmol) NaOH, 0.5 g crushed ice and 1.5 mL of cold water. To this mixture, 0.156 mL (3.02 mmol, 0.483 g) of Br_2_ was added while stirring. It was stirred for five minutes and then the product was filtered, washed with cold water and dried in a desiccator to isolate 0.348 g (71%) of NBS.

#### 3.2.6. Detailed Procedures for the Preparation of Synthesized Compounds

*Methyl benzoate* (**1a**) [17]*.* Synthesized according to the general procedure. Reaction conditions: benzoic acid (1 mmol, 122.1 mg), MeOH (0.5 mL), NBS (0.070 mmol, 12.5 mg), 70 °C, 20 h. Purification: Not necessary. Yield: 122 mg of colourless oil (90%); ^1^H-NMR (300 MHz, CDCl_3_) δ 8.07–8.01 (m, 2H), 7.59–7.51 (m, 1H), 7.43 (ddt, *J* = 8.2, 6.8, 1.1 Hz, 2H), 3.91 (s, 3H); ^13^C-NMR (76 MHz, CDCl3) δ 167.2, 133.0, 130.3, 129.7, 128.4, 52.2; HRMS (ESI) for C_8_H_8_O_2_: calculated *m*/*z* = 137.0603 (MH^+^); found *m*/*z* = 137.0606 (MH^+^).

*Methyl octanoate* (**2a**) [58]*.* Synthesized according to the general procedure. Reaction conditions: octanoic acid (1 mmol, 158.5 μL), MeOH (0.5 mL), NBS (0.070 mmol, 12.5 mg), 70 °C, 2 h. Purification: Not necessary. Yield: 153 mg of colourless oil (97%); ^1^H-NMR (300 MHz, CDCl_3_) δ 3.67 (s, 3H), 2.30 (t, *J* = 7.5 Hz, 2H), 1.69–1.56 (m, 2H), 1.35–1.25 (m, 8H), 0.88 (t, *J* = 6.9 Hz, 3H); ^13^C-NMR (76 MHz, CDCl_3_) δ 174.5, 51.5, 34.2, 31.8, 29.2, 29.0, 25.1, 22.7, 14.2; HRMS (ESI) for C_9_H_18_O_2_: calculated *m*/*z* = 159.1385 (MH^+^); found *m*/*z* = 159.1389 (MH^+^).

*Methyl 4-nitrobenzoate* (**3a**) [17]*.* Synthesized according to the general procedure. Reaction conditions: 4-nitrobenzoic acid (1 mmol, 167.1 mg), MeOH (1.5 mL), NBS (0.070 mmol, 12.5 mg), 70 °C, 20 h. Purification: Not necessary. Yield: 167 mg of white solid (92%); ^1^H-NMR (300 MHz, CDCl_3_) δ 8.34–8.19 (m, 4H), 3.99 (s, 3H); ^13^C-NMR (76 MHz, CDCl_3_) δ 165.2, 150.6, 135.6, 130.8, 123.6, 77.2, 52.9.

*Methyl 3-nitrobenzoate* (**4a**) [59]. Synthesized according to the general procedure. Reaction conditions: 3-nitrobenzoic acid (1 mmol, 167.1 mg), MeOH (2 mL), NBS (0.070 mmol, 12.5 mg), 70 °C, 20 h. Purification: Not necessary. Yield: 141 mg of yellow solid (78%); ^1^H-NMR (300 MHz, CDCl_3_) δ 8.94–8.75 (m, 1H), 8.50–8.30 (m, 2H), 7.68 (t, *J* = 8.0 Hz, 1H), 4.00 (s, 3H); ^13^C-NMR (76 MHz, CDCl_3_) δ 165.0, 148.3, 135.3, 131.9, 129.7, 127.4, 124.6, 52.8; HRMS (ESI) for C_8_H_7_NO_4_: calculated *m*/*z* = 182.0453 (MH^+^); found *m*/*z* = 182.0457 (MH^+^).

*Methyl 4-fluorobenzoate* (**5a**) [60]*.* Synthesized according to the general procedure. Reaction conditions: 4-fluorobenzoic acid (1 mmol, 140.1 mg), MeOH (2 mL), NBS (0.070 mmol, 12.5 mg), 70 °C, 20 h. Purification: Not necessary. Yield: 105 mg of colourless oil (68%); ^1^H-NMR (300 MHz, CDCl_3_) δ 8.02 (ddd, *J* = 10.1, 5.2, 2.5 Hz, 2H), 7.13–7.03 (m, 2H), 3.89 (s, 3H); ^13^C-NMR (76 MHz, CDCl3) δ 166.2, 165.8 (d, *J* = 253.5 Hz) 132.2 (d, *J* = 9.4 Hz), 126.5 (d, *J* = 3.0 Hz), 115.6 (d, *J* = 22.0 Hz), 52.2; ^19^F-NMR (285 MHz, CDCl3) δ -106.34 (tt, *J* = 8.5, 5.4 Hz); HRMS (ESI) for C_8_H_7_FO_2_: calculated *m*/*z* = 155.0508 (MH^+^); found *m*/*z* = 155.0505 (MH^+^).

*Methyl 3-fluorobenzoate* (**6a**) [61]*.* Synthesized according to the general procedure. Reaction conditions: 3-fluorobenzoic acid (1 mmol, 140.1 mg), MeOH (0.5 mL), NBS (0.070 mmol, 12.5 mg), 70 °C, 20 h. Purification: Not necessary. Yield: 108 mg of colourless oil (70%); ^1^H-NMR (300 MHz, CDCl_3_) δ 7.83 (dt, *J* = 7.7, 1.2 Hz, 1H), 7.71 (ddd, *J* = 9.4, 2.6, 1.5 Hz, 1H), 7.41 (td, *J* = 8.0, 5.6 Hz, 1H), 7.25 (tdd, *J* = 8.3, 2.5, 1.0 Hz, 1H), 3.92 (s, 3H); ^13^C-NMR (76 MHz, CDCl_3_) δ 166.0, 162.6 (d, *J* = 246.6 Hz), 132.4 (d, *J* = 7.6 Hz), 130.1 (d, *J* = 7.6 Hz), 125.4 (d, *J* = 2.9 Hz), 120.1 (d, *J* = 21.4 Hz), 116.6 (d, *J* = 23.0 Hz), 52.4; ^19^F-NMR (285 MHz, CDCl_3_) δ -112.92 (ddd, *J* = 9.4, 8.4, 5.6 Hz); HRMS (ESI) for C_8_H_7_FO_2_: calculated *m*/*z* = 155.0508 (MH^+^); found *m*/*z* = 155.0511 (MH^+^).

*Methyl 4-methylbenzoate* (**7a**) [60]*.* Synthesized according to the general procedure. Reaction conditions: 4-methylbenzoic acid (1 mmol, 136.1 mg), MeOH (2 mL), NBS (0.070 mmol, 12.5 mg), 70 °C, 20 h. Purification: Not necessary. Yield: 126 mg of colourless oil (84%); ^1^H-NMR (300 MHz, CDCl_3_) δ 7.97–7.86 (m, 2H), 7.22 (d, *J* = 8.2 Hz, 2H), 3.88 (s, 3H), 2.39 (s, 3H); ^13^C-NMR (76 MHz, CDCl_3_) δ 167.2, 143.6, 129.6, 129.1, 127.5, 52.0, 21.7; HRMS (ESI) for C_9_H_10_O_2_: calculated *m*/*z* = 151.0759 (MH^+^); found *m*/*z* = 151.0755 (MH^+^).

*Methyl 3-methylbenzoate* (**8a**) [60]*.* Synthesized according to the general procedure. Reaction conditions: 3-methylbenzoic acid (1 mmol, 136.1 mg), MeOH (2 mL), NBS (0.070 mmol, 12.5 mg), 70 °C, 20 h. Purification: Not necessary. Yield: 136 mg of colourless oil (90%); ^1^H-NMR (300 MHz, CDCl_3_) δ 7.88–7.81 (m, 1H), 7.39–7.28 (m, 1H), 3.91 (s, 2H), 2.40 (s, 1H); ^13^C-NMR (76 MHz, CDCl_3_) δ 167.3, 138.2, 133.7, 130.2, 130.2, 128.3, 126.8, 52.1, 21.3; HRMS (ESI) for C_9_H_10_O_2_: calculated *m*/*z* = 151.0759 (MH^+^); found *m*/*z* = 151.0762 (MH^+^).

*Methyl 3-methoxybenzoate* (**10a**) [62]. Synthesized according to the general procedure. Reaction conditions: 3-methoxybenzoic acid (1 mmol, 152.1 mg), MeOH (2 mL), NBS (0.070 mmol, 12.5 mg), 70 °C, 20 h. Purification: Not necessary. Yield: 75 mg of yellow oil (45%); ^1^H-NMR (300 MHz, CDCl_3_) δ 7.66–7.53 (m, 2H), 7.33 (t, *J* = 7.9 Hz, 1H), 7.09 (ddd, *J* = 8.2, 2.6, 0.9 Hz, 1H), 3.91 (s, 3H), 3.84 (s, 3H); ^13^C-NMR (76 MHz, CDCl_3_) δ 167.0, 159.6, 131.5, 129.4, 122.0, 119.5, 114.0, 55.4, 52.2; HRMS (ESI) for C_9_H_10_O_3_: calculated *m*/*z* = 167.0708 (MH^+^); found *m*/*z* = 167.0717 (MH^+^).

*Methyl 4-octylbenzoate* (**11a**) [63]*.* Synthesized according to the general procedure. Reaction conditions: 4-octylbenzoic acid (1 mmol, 234.3 mg), MeOH (2 mL), NBS (0.070 mmol, 12.5 mg), 70 °C, 20 h. Purification: Not necessary. Yield: 194 mg of yellow oil, (78%); ^1^H-NMR (300 MHz, CDCl_3_) δ 8.02–7.87 (m, 2H), 7.31–7.16 (m, 2H), 3.89 (s, 3H), 2.73–2.57 (m, 2H), 1.69–1.54 (m, 2H), 1.43–1.19 (m, 10H), 0.87 (t, *J* = 6.7 Hz, 3H); ^13^C-NMR (76 MHz, CDCl_3_) δ 167.3, 148.6, 129.7, 128.5, 127.7, 52.0, 36.1, 32.0, 31.2, 29.5, 29.4, 29.3, 22.8, 14.2; HRMS (ESI) for C_16_H_24_O_2_: calculated *m*/*z* = 249.1855 (MH^+^); found *m*/*z* = 249.1853 (MH^+^).

*Methyl 2-iodobenzoate* (**12a**) [64]. Synthesized according to the general procedure. Reaction conditions: 2-iodobenzoic acid (1 mmol, 248.0 mg), MeOH (0.5 mL), NBS (0.070 mmol, 12.5 mg), 70 °C, 20 h. Purification: Not necessary. Yield: 144 mg of colourless oil (55%); ^1^H-NMR (300 MHz, CDCl_3_) δ 7.95 (dd, *J* = 7.9, 1.1 Hz, 1H), 7.76 (dd, *J* = 7.8, 1.7 Hz, 1H), 7.36 (td, *J* = 7.6, 1.1 Hz, 1H), 7.11 (td, *J* = 7.7, 1.7 Hz, 1H), 3.90 (s, 3H); ^13^C-NMR (76 MHz, CDCl_3_) δ 166.9, 141.3, 135.1, 132.7, 130.9, 127.9, 94.1, 52.5; HRMS (ESI) for C_8_H_7_IO_2_: calculated *m*/*z* = 262.9569 (MH^+^); found *m*/*z* = 262.9563 (MH^+^).

*Methyl stearate* (**13a**) [14]*.* Synthesized according to the general procedure. Reaction conditions: stearic acid (1 mmol, 284.5 mg), MeOH (0.5 mL), NBS (0.070 mmol, 12.5 mg), 70 °C, 20 h. Purification: Not necessary. Yield: 285 mg of white solid (95%); ^1^H-NMR (300 MHz, CDCl_3_) δ 3.67 (s, 3H), 2.30 (t, *J* = 7.5 Hz, 2H), 1.69–1.55 (m, 2H), 1.37–1.20 (m, 28H), 0.88 (t, *J* = 7.0 Hz, 3H); ^13^C-NMR (76 MHz, CDCl_3_) δ 174.5, 51.6, 34.3, 32.1, 29.8, 29.8, 29.8, 29.8, 29.8, 29.8, 29.8, 29.7, 29.6, 29.5, 29.4, 29.3, 25.1, 22.8, 14.3; HRMS (ESI) for C_19_H_38_O_2_: calculated *m*/*z* = 299.2950 (MH^+^); found *m*/*z* = 299.2957 (MH^+^).

*Methyl oleate* (**14a**) [14]*.* Synthesized according to the general procedure. Reaction conditions: oleic acid (1 mmol, 315.6 µL), MeOH (2 mL), NBS (0.070 mmol, 12.5 mg), 70 °C, 15 h. Purification: Not necessary. Yield: 289 mg of colorless oil, (97%); ^1^H-NMR (300 MHz, CDCl_3_) δ 5.34 (ddd, *J* = 5.6, 3.5, 2.1 Hz, 2H), 3.66 (s, 3H), 2.30 (t, *J* = 7.5 Hz, 2H), 2.10–1.91 (m, 4H), 1.71–1.54 (m, 2H), 1.42–1.17 (m, 18H), 0.88 (t, *J* = 6.7 Hz, 3H); ^13^C-NMR (76 MHz, CDCl_3_) δ 174.4, 130.1, 129.9, 51.5, 34.2, 32.0, 29.9, 29.8, 29.7, 29.5, 29.4, 29.3, 29.3, 29.2, 27.3, 27.3, 25.1, 22.8, 14.2; HRMS (ESI) for C_19_H_36_O_2_: calculated *m*/*z* = 297.2794 (MH^+^); found *m*/*z* = 297.2798 (MH^+^).

*Dimethyl oxalate* (**15a**) [65]*.* Synthesized according to the general procedure. Reaction conditions: oxalic acid (1 mmol, 90.0 mg), MeOH (2 mL), NBS (0.070 mmol, 12.5 mg), 70 °C, 15 h. Purification: Not necessary. Yield: 110 mg of white solid (93%); ^1^H-NMR (300 MHz, CDCl_3_) δ 3.92 (s, 6H); ^13^C-NMR (76 MHz, CDCl_3_) δ 157.8, 53.5; HRMS (ESI) for C_4_H_6_O_4_: calculated *m*/*z* = 119.0344 (MH^+^); found *m*/*z* = 119.0342 (MH^+^).

*Trimethyl citrate* (**16a**) [66]*.* Synthesized according to the general procedure. Reaction conditions: citric acid (1 mmol, 210.1 mg), MeOH (2 mL), NBS (0.070 mmol, 12.5 mg), 70 °C, 20 h. Purification: Not necessary. Yield: 220 mg of white solid (94%); ^1^H-NMR (300 MHz, CDCl_3_) δ 4.10 (s, 1H), 3.81 (s, 3H), 3.67 (s, 6H), 2.89 (d, *J* = 15.6 Hz, 2H), 2.79 (d, *J* = 15.6 Hz, 2H); ^13^C-NMR (76 MHz, CDCl_3_) δ 173.8, 170.2, 73.3, 53.2, 52.0, 43.1; HRMS (ESI) for C_9_H_14_O_7_: calculated *m*/*z* = 235.0818 (MH^+^); found *m*/*z* = 235.0815 (MH^+^).

*Adamantane-1-carboxylic acid methyl ester* (**17a**) [61]*.* Synthesized according to the general procedure. Reaction conditions: 1-adamantanecarboxylic acid (1 mmol, 180.2 mg), MeOH (0.5 mL), NBS (0.070 mmol, 12.5 mg), 70 °C, 20 h. Purification: Not necessary. Yield: 186 mg of white solid (96%); ^1^H-NMR (300 MHz, CDCl_3_) δ 3.60 (s, 3H), 2.01–1.92 (m, 3H), 1.88–1.80 (m, 6H), 1.74–1.59 (m, 3H); ^13^C-NMR (76 MHz, CDCl_3_) δ 178.08, 51.44, 40.64, 38.81, 36.46, 27.91; HRMS (ESI) for C_12_H_18_O_2_: calculated *m*/*z* = 195.1385 (MH^+^); found *m*/*z* = 195.1382 (MH^+^).

*Methyl 3,7,12-trioxo-5β-cholan-24-oate* (**18a**) [67]*.* The mixture of dehydrocholic acid (0.25 mmol, 101.6 mg), MeOH (2 mL) and *N*-bromosuccinimide (0.018 mmol, 3.1 mg) was stirred in a 25 mL reactor tube at 70 °C for 1 h. After the completion of the reaction, the mixture was cooled to room temperature and MeOH was evaporated under the reduced pressure. The residue was dissolved in ethyl acetate and first washed with the mixture of 10 mL of 10% Na_2_S_2_O_3 (aq)_, 5 mL of saturated NaHCO_3 (aq)_ and 10 mL of distilled water and then with 10 mL of 10% HCl _(aq)._ The water phase was extracted with ethyl acetate (3 × 5 mL). The organic layers were combined, dried with Na_2_SO_4_ and the solvent was evaporated under the reduced pressure. Purification: Not necessary. Yield: 93 mg of white solid (89%); ^1^H-NMR (300 MHz, CDCl_3_) δ 3.66 (s, 3H), 3.01–2.75 (m, 3H), 2.51–1.16 (m, 21H), 1.41 (s, 3H), 1.08 (s, 3H), 0.85 (d, *J* = 6.5 Hz, 3H); ^13^C-NMR (76 MHz, CDCl_3_) δ 212.0, 209.1, 208.8, 174.5, 56.9, 51.8, 51.5, 49.0, 46.9, 45.7, 45.6, 45.0, 42.8, 38.7, 36.5, 36.1, 35.6, 35.3, 31.3, 30.5, 27.7, 25.2, 22.0, 18.7, 11.9; HRMS (ESI) for C_25_H_36_O_5_: calculated *m*/*z* = 417.2641 (MH^+^); requires *m*/*z* = 417.2644 (MH^+^).

*Methyl 3α,7α,12α-trihydroxy-5β-cholan-24-oate* (**19a**) [68]*.* The mixture of cholic acid (0.25 mmol, 102.1 mg), MeOH (2 mL) and *N*-bromosuccinimide (0.018 mmol, 3.1 mg) was stirred in a 25 mL reactor tube at 70 °C for 1 h. After the completion of the reaction, the mixture was cooled to room temperature and MeOH was evaporated under the reduced pressure. The residue was dissolved in ethyl acetate and washed with the mixture of 10 mL of 10% Na_2_S_2_O_3 (aq)_, 5 mL of saturated NaHCO_3(aq)_ and 10 mL of distilled water. The water phase was extracted with ethyl acetate (3 × 5 mL). The organic layers were combined, dried with Na_2_SO_4_ and the solvent was evaporated under the reduced pressure. Purification: Not necessary. Yield: 93 mg of white solid (88%); ^1^H-NMR (300 MHz, CDCl_3_) δ 3.95 (s, 1H), 3.83 (s, 1H), 3.66 (s, 3H), 3.55 (s, 3H), 3.49–3.32 (m, 1H), 2.48–1.07 (m, 24H), 0.98 (d, *J* = 5.8 Hz, 3H), 0.88 (s, 3H), 0.67 (s, 3H); ^13^C-NMR (76 MHz, CDCl_3_) δ 174.7, 72.9, 71.7, 68.3, 51.3, 46.8, 46.2, 41.4, 41.4, 39.3, 39.3, 35.2, 35.2 34.6, 34.6, 31.0, 30.8, 30.2, 28.0, 27.4, 26.1, 23.1, 22.3, 17.2, 12.3; HRMS (ESI) for C_25_H_42_O_5_: calculated *m*/*z* = 423.3110 (MH^+^); found *m*/*z* = 423.3128 (MH^+^).

*Methyl 2-cyanoacetate* (**20a**) [69]. Synthesized according to the general procedure. Reaction conditions: Cyanoacetic acid (1 mmol, 85.1 mg), MeOH (0.5 mL), NBS (0.070 mmol, 12.5 mg), 70 °C, 20 h. Purification: Not necessary. Yield: 96 mg of colourless oil (97%); ^1^H-NMR (300 MHz, CDCl_3_) δ 3.76 (s, 3H), 3.46 (s, 2H); ^13^C-NMR (76 MHz, CDCl_3_) δ 163.6, 113.2, 53.5, 24.4; HRMS (ESI) for C_4_H_5_NO_2_: calculated *m*/*z* = 100.0399 (MH^+^); found *m*/*z* = 100.0398 (MH^+^).

*(S)-Methyl 2-acetamido-3-phenylpropanoate* (**21a**) [70]*.* Synthesized according to the general procedure. Reaction conditions: *N*-Acetyl-l-phenylalanine (1 mmol, 207.2 mg), MeOH (0.5 mL), NBS (0.070 mmol, 12.5 mg), 70 °C, 20 h. Purification: Not necessary. Yield: 208 mg of white solid (91%); ^1^H-NMR (300 MHz, CDCl_3_) δ 7.33–7.18 (m, 3H), 7.18–7.05 (m, 2H), 6.38 (d, *J* = 7.5 Hz, 1H), 4.95–4.77 (m, 1H), 3.69 (s, 3H), 3.21–2.93 (m, 2H), 1.95 (s, 3H); ^13^C-NMR (76 MHz, CDCl_3_) δ 172.2, 169.8, 136.0, 129.2, 128.5, 127.0, 53.2, 52.2, 37.8, 22.9; HRMS (ESI) for C_12_H_15_NO_3_: calculated *m*/*z* = 222.1130 (MH^+^); found *m*/*z* = 222.1135 (MH^+^).

*Methyl 2-(1H-indol-3-yl)acetate* (**22a**) [71]*.* Synthesized according to the general procedure. Reaction conditions: Heteroauxin (1 mmol, 175.2 mg), MeOH (0.5 mL), NBS (0.070 mmol, 12.5 mg), 70 °C, 20 h. Purification: Not necessary. Yield: 185 mg of brown oil (98%); ^1^H-NMR (300 MHz, CDCl_3_) δ 8.16 (s, 1H), 7.56 (dd, *J* = 6.7, 1.6 Hz, 2H), 7.24–6.98 (m, 3H), 6.84 (d, *J* = 2.4 Hz, 1H), 3.72 (s, 2H), 3.63 (s, 3H); ^13^C-NMR (76 MHz, CDCl_3_) δ 173.0, 136.1, 127.1, 123.4, 122.0, 119.5, 118.6, 111.4, 107.8, 52.0, 31.1; HRMS (ESI) for C_11_H_11_NO_2_: calculated *m*/*z* = 190.0868 (MH^+^); found *m*/*z* = 190.0865 (MH^+^).

*Methyl 4-(4-chlorophenyl)-4-oxobutanoate* (**23a**) [72]*.* Synthesized according to the general procedure. Reaction conditions: 3-(4-Chlorobenzoyl)propionic acid (1 mmol, 212.6 mg), MeOH (0.5 mL), NBS (0.070 mmol, 12.5 mg), 70 °C, 20 h. Purification: Not necessary. Yield: 224 mg of white solid (99%); ^1^H-NMR (300 MHz, CDCl_3_) δ 7.90–7.81 (m, 2H), 7.41–7.33 (m, 2H), 3.64 (s, 3H), 3.22 (t, *J* = 6.6 Hz, 2H), 2.70 (t, *J* = 6.6 Hz, 2H); ^13^C-NMR (76 MHz, CDCl_3_) δ 196.8, 173.2, 139.6, 134.8, 129.4, 128.9, 51.8, 33.3, 27.9; HRMS (ESI) for C_12_H_18_O_2_: calculated *m*/*z* = 249.0294 (MNa^+^); found *m*/*z* = 249.0297 (MNa^+^).

*2-Fluoroethyl benzoate* (**1b**) [73]*.* Synthesized according to the general procedure. Reaction conditions: benzoic acid (1 mmol, 122.1 mg), 2-fluoroethanol (0.5 mL), NBS (0.07 mmol, 12.5 mg), 70 °C, 40 h. Purification: Preparative TLC (CH_2_Cl_2_/MeOH = 200:1). Yield: 160 mg of colourless oil (95%); ^1^H-NMR (300 MHz, CDCl_3_) δ 8.20–7.86 (m, 2H), 7.60–7.52 (m, 1H), 7.49–7.37 (m, 2H), 4.85–4.44 (m, 4H); ^13^C-NMR (76 MHz, CDCl_3_) δ 166.4, 133.3, 129.8, 128.5, 81.5 (d, *J* = 170.6 Hz), 63.9 (d, *J* = 20.2 Hz); ^19^F NMR (285 MHz, CDCl3) δ 5.03 (tt, *J* = 47.4, 28.6 Hz); HRMS (ESI) for C_9_H_9_FO_2_: calculated *m*/*z* = 169.0665 (MH^+^); found *m*/*z* = 169.0668 (MH^+^).

*Isopropyl benzoate (**1c**)* [74]*.* Synthesized according to the general procedure. Reaction conditions: benzoic acid (1 mmol, 122.1 mg), isopropanol (0.5 mL), NBS (0.07 mmol, 12.5 mg), 70 °C, 20 h. Purification: Not necessary. Yield: 12 mg of colourless oil (7%); ^1^H-NMR (300 MHz, CDCl_3_) δ 8.09–7.99 (m, 2H), 7.59–7.50 (m, 1H), 7.47–7.38 (m, 2H), 5.26 (hept, *J* = 6.3 Hz, 1H), 1.37 (d, *J* = 6.3 Hz, 6H); ^13^C-NMR (76 MHz, CDCl_3_) δ 166.3, 132.8, 131.1, 129.6, 128.4, 68.5, 22.1.

*2-Fluoroethyl octanoate* (**2b**) [75]*.* Synthesized according to the general procedure. Reaction conditions: octanoic acid (1 mmol, 158.5 μL), 2-fluoroethanol (0.5 mL), NBS (0.07 mmol, 12.5 mg), 70 °C, 20 h. Purification: Not necessary. Yield: 175 mg of yellow oil (92%); ^1^H-NMR (300 MHz, CDCl_3_) δ 4.73–4.20 (m, 4H), 2.36 (t, *J* = 7.5 Hz, 2H), 1.71–1.57 (m, 2H), 1.42–1.19 (m, 8H), 0.88 (t, *J* = 7.1 Hz, 3H); ^13^C-NMR (76 MHz, CDCl_3_) δ 173.7, 81.5 (d, *J* = 170.3 Hz), 63.2 (d, *J* = 20.1 Hz), 34.2, 31.7, 29.1, 29.0, 25.0, 22.7, 14.1; ^19^F-NMR (285 MHz, CDCl3) δ 4.86 (tt, *J* = 47.4, 28.7 Hz); HRMS (ESI) for C_10_H_19_FO_2_: calculated *m*/*z* = 191.1447 (MH^+^); found *m*/*z* = 191.1446 (MH^+^).

*Isopropyl octanoate* (**2c**) [76]*.* Synthesized according to the general procedure. Reaction conditions octanoic acid (1 mmol, 158.5 μL), isopropanol (0.5 mL), NBS (0.07 mmol, 12.5 mg), 70 °C, 20 h. Purification: Not necessary. Yield: 149 mg of colourless oil (80%); ^1^H-NMR (300 MHz, CDCl_3_) δ 5.00 (hept, *J* = 6.3 Hz, 1H), 2.25 (t, *J* = 7.5 Hz, 2H), 1.67–1.55 (m, 2H), 1.35–1.25 (m, 8H), 1.23 (d, *J* = 6.3 Hz, 6H), 0.88 (t, *J* = 7.0 Hz, 3H); ^13^C-NMR (76 MHz, CDCl_3_) δ 173.4, 67.3, 34.8, 31.7, 29.2, 29.0, 25.1, 22.7, 21.9, 14.1; HRMS (ESI) for C_11_H_22_O_2_: calculated *m*/*z* = 187.1698 (MH^+^); found *m*/*z* = 187.1703 (MH^+^).

*n-Butyl benzoate* (**1d**) [77]*.* Synthesized according to the general procedure. Reaction conditions: benzoic acid (1 mmol, 122.1 mg), *n*-butanol (0.5 mL), NBS (0.07 mmol, 12.5 mg), 70 °C, 20 h. Purification: Preparative TLC (CH_2_Cl_2_/MeOH = 200:1). Yield: 134 mg of colourless oil (75%); ^1^H-NMR (300 MHz, CDCl_3_) δ 8.10–7.99 (m, 2H), 7.58–7.49 (m, 1H), 7.42 (t, *J* = 7.5 Hz, 2H), 4.32 (t, *J* = 6.6 Hz, 2H), 1.84–1.66 (m, 2H), 1.56–1.39 (m, 2H), 0.98 (t, *J* = 7.4 Hz, 3H); ^13^C-NMR (76 MHz, CDCl3) δ 166.7, 132.8, 130.6, 129.6, 128.4, 64.9, 30.9, 19.4, 13.8; HRMS (ESI) for C_11_H_14_O_2_: calculated *m*/*z* = 179.1072 (MH^+^); found *m*/*z* = 179.1070 (MH^+^).

*n-Butyl octanoate* (**2d**) [4]*.* Synthesized according to the general procedure. Reaction conditions octanoic acid (1 mmol, 158.5 μL), *n*-butanol (1 mmol, 92 μL), NBS (0.07 mmol, 12.5 mg), 70 °C, 15 h. Purification: Not necessary. Yield: 190 mg of colourless oil (95%); ^1^H-NMR (300 MHz, CDCl_3_) δ 4.07 (t, *J* = 6.6 Hz, 2H), 2.29 (t, *J* = 7.5 Hz, 2H), 1.70–1.52 (m, 4H), 1.47–1.21 (m, 10H), 0.94 (t, *J* = 7.3 Hz, 3H), 0.87 (t, *J* = 6.9 Hz, 3H); ^13^C-NMR (76 MHz, CDCl_3_) δ 174.0, 64.1, 34.4, 31.8, 30.8, 29.2, 29.0, 25.1, 22.7, 19.2, 14.1, 13.7; HRMS (ESI) for C_12_H_24_O_2_: calculated *m*/*z* = 201.1855 (MH^+^); found *m*/*z* = 201.1857 (MH^+^).

*n-Octyl benzoate (**1f**)* [58]*.* Synthesized according to the general procedure. Reaction conditions: benzoic acid (1 mmol, 122.1 mg), *n*-octanol (1 mmol, 158 μL), NBS (0.07 mmol, 12.5 mg), 70 °C, 20 h. Purification: Preparative TLC (CH_2_Cl_2_/Hexane = 1:1). Yield: 134 mg of white solid (57%); ^1^H-NMR (300 MHz, CDCl3) δ 8.10–8.00 (m, 2H), 7.59–7.51 (m, 1H), 7.47–7.39 (m, 2H), 4.31 (t, *J* = 6.7 Hz, 2H), 1.83–1.70 (m, 2H), 1.51–1.21 (m, 10H), 0.88 (t, *J* = 6.6 Hz, 3H); ^13^C-NMR (76 MHz, CDCl_3_) δ 166.8, 132.9, 130.7, 129.7, 128.4, 65.3, 31.9, 29.4, 29.3, 28.9, 26.2, 22.8, 14.2; HRMS (ESI) for C_15_H_22_O_2_: calculated *m*/*z* = 235.1698 (MH^+^); found *m*/*z* = 235.1697 (MH^+^).

*n-Octyl octanoate* (**2f**) [78]*.* Synthesized according to the general procedure. Reaction conditions: octanoic acid (1 mmol, 158.5 μL), *n*-octanol (1 mmol, 158 μL), NBS (0.07 mmol, 12.5 mg), 70 °C, 20 h. Purification: Preparative TLC (CH_2_Cl_2_/MeOH = 200:1). Yield: 223 mg of colourless oil (87%); ^1^H-NMR (300 MHz, CDCl_3_) δ 4.06 (t, *J* = 6.7 Hz, 2H), 2.28 (t, *J* = 7.5 Hz, 2H), 1.68–1.54 (m, 4H), 1.39–1.19 (m, 18H), 0.88 (t, *J* = 6.3 Hz, 6H); ^13^C-NMR (76 MHz, CDCl_3_) δ 173.9, 64.4, 34.4, 31.9, 31.8, 29.3, 29.3, 29.2, 29.0, 28.8, 26.0, 25.1, 22.7, 22.7, 14.1, 14.1; HRMS (ESI) for C_16_H_32_O_2_: calculated *m*/*z* = 257.2481 (MH^+^); found *m*/*z* = 257.2486 (MH^+^).

*Cyclopentyl octanoate* (**2g**) [79]*.* Synthesized according to the general procedure. Reaction conditions: octanoic acid (1 mmol, 158.5 μL), cyclopentanol (1 mmol, 91 μL), NBS (0.07 mmol, 12.5 mg), 70 °C, 15 h. Purification: Not necessary. Yield: 200 mg of colourless oil (94%); ^1^H-NMR (300 MHz, CDCl_3_) δ 5.16 (tt, *J* = 5.9, 2.6 Hz, 1H), 2.25 (t, *J* = 7.5 Hz, 2H), 1.95–1.47 (m, 10H), 1.41–1.20 (m, 8H), 0.88 (d, *J* = 7.0 Hz, 3H); ^13^C-NMR (76 MHz, CDCl_3_) δ 173.8, 76.8, 34.8, 32.8, 31.8, 29.2, 29.0, 25.2, 23.8, 22.7, 14.1; HRMS (ESI) for C_13_H_24_O_2_: calculated *m*/*z* = 213.1855 (MH^+^); found *m*/*z* = 213.1860 (MH^+^).

## 4. Conclusions

In conclusion, a convenient and selective metal-free method for direct dehydrative esterification of free aromatic and aliphatic acids with different alcohols has been developed, using inexpensive and easy-to-handle *N-*bromosuccinimide as a moisture- and air-tolerant recyclable catalyst. The synthesis has been performed under neat reaction conditions without the need for simultaneous removal of water and excessive reagents. In spite of some scope limitations, the method provides good to excellent product yields and in the majority of cases, enables simple isolation procedure only by extraction. Even in the case of di- and tri-carboxy aliphatic acids, esterification has been successfully accomplished with the same amount of NBS catalyst as in the case of mono-carboxy alkyl acids. The applicability of the method has been successfully demonstrated also on steroidal carboxylic acids. The large-scale synthesis of methyl benzoate, methyl stearate and trimethyl citrate as examples of commercially significant esters has been performed with high to excellent yields (85–100%).

## Figures and Tables

**Table 1:**
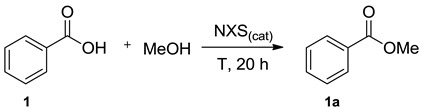
Catalyst loading and temperature optimization studies ^1^.

Entry	NXS	Loading [mol%]	Temperature [°C]	Conversion of Benzoic Acid to Methyl Benzoate [%] ^2^
1	/	/	70	0
2	NCS	15	70	17
3	NBS	15	70	94
4	NIS	15	70	30
5	NBS	10	70	94
6	NBS	7	70	94
7	NBS	3	70	76
8 ^3^	NBS	7	70	<10
9	NBS	7	30	0
10	NBS	7	50	75
11	NBS	7	100	94
12	HCl	7	70	93
13	HBr	7	70	84
14	HI	7	70	68
15	Br_2_	7	70	94

^1^ Reaction conditions: **1** (1.0 mmol), MeOH (0.5 mL), NXS, HCl (37%), HBr (48%), HI (57%), Br_2_, T, 20 h. ^2^ Conversions were determined by ^1^H-NMR analysis of the crude reaction mixtures. ^3^ Freshly dried MeOH was used and the reaction performed over anhydrous Na_2_SO_4_.

**Table 2:**
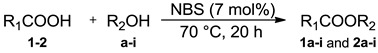
The effect of alcohol structure on esterification of benzoic (**1**) and octanoic acid (**2**) ^1,2,3^.

R_2_OH	R_1_ = Ph	R_1_ = Heptyl
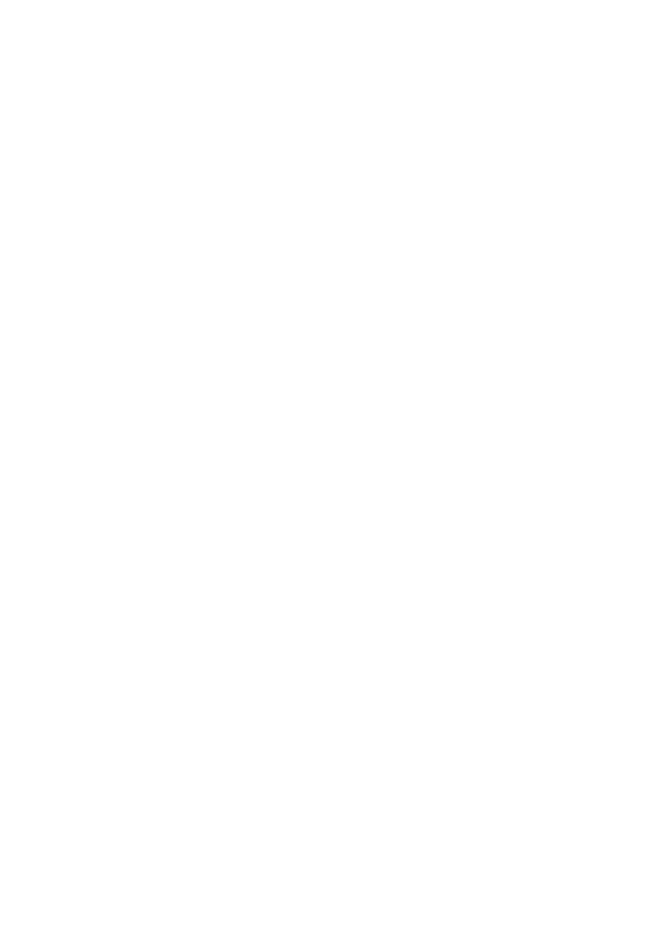		

^1^ Reaction conditions: carboxylic acid (1 mmol), alcohol (1 mmol, 2 mmol or 0.5 mL), NBS (0.07 mmol), 70 °C, 20 h. ^2^ Conversions were determined by ^1^H NMR spectroscopy. ^3^ The values in brackets stand for yields of isolated products. ^4^ Conversion (yield of isolated product) after 40 h. ^5^ Conversion (yield of isolated product) after 2 h. ^6^ Conversion (yield of isolated product) after 15 h.

**Table 3:**
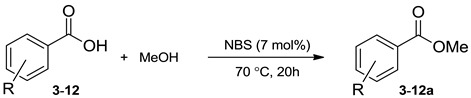
Esterification of substituted benzoic and different alkyl carboxylic acids with MeOH in the presence of NBS ^1,2,3^.

**Methyl Benzoates**
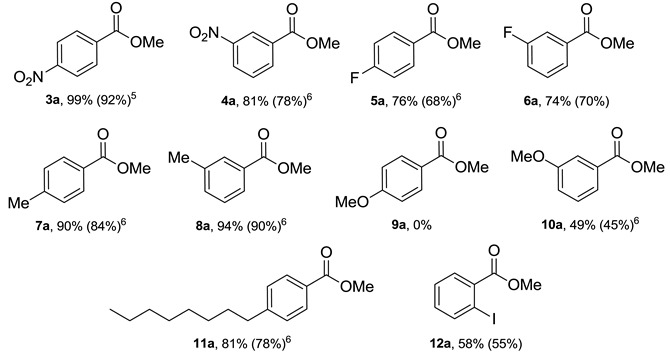
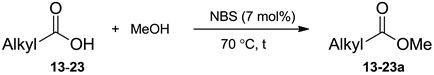
**Methyl Alkyl Esters and Methyl Esters of Cholic Acid Derivatives**
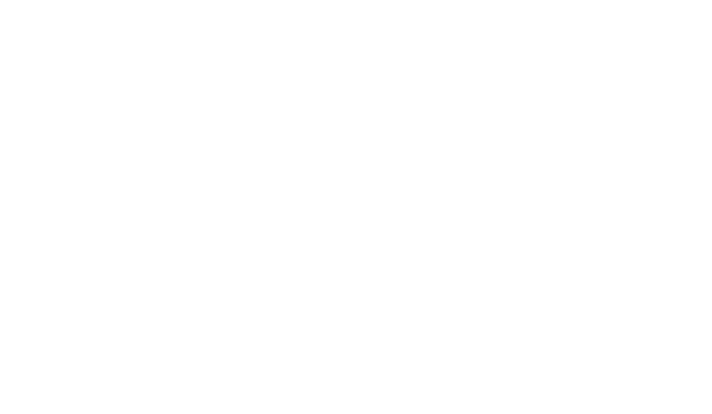

^1^ Reaction conditions: carboxylic acid (1 mmol), MeOH (0.5 mL), NBS (0.07 mmol), 70 °C, 1–20 h. ^2^ Conversions were determined by ^1^H-NMR spectroscopy. ^3^ The values in brackets stand for yields of isolated products. ^4^ Reaction conditions: carboxylic acid (0.25 mmol), MeOH (2 mL), NBS (0.018 mmol), 70 °C, 1 h; conversion (yield of isolated product) after 1 h. ^5^ 1.5 mL of MeOH was used. ^6^ 2 mL of MeOH was used.

## Supplementary Materials

The following are available online, ^1^H-NMR, ^13^C-NMR and ^19^F-NMR spectra of isolated final products.

## References

[1] Otera J. Esterification (2004). Esterification.

[2] Baskar G., Aiswarya R. (2016). Trends in catalytic production of biodiesel from various feedstocks. Renew. Sustain. Energy Rev..

[3] Talebian-Kiakalaieh A., Amin N.A.S., Mazaheri H. (2013). A review on novel processes of biodiesel production from waste cooking oil. Appl. Energy.

[4] Hosseini-Sarvari M., Sodagar E. (2013). Esterification of free fatty acids (Biodiesel) using nano sulfated-titania as catalyst in solvent-free conditions. C R. Chim..

[5] Mallesham B., Govinda Rao B., Reddy B.M. (2016). Production of biofuel additives by esterification and acetalization of bioglycerol. C R. Chim..

[6] Tsakos M., Schaffert E.S., Clement L.L., Villadsen N.L., Poulsen T.B. (2015). Ester coupling reactions-An enduring challenge in the chemical synthesis of bioactive natural products. Nat. Prod. Rep..

[7] Larock R.C., Dubrovskiy A.V., Markina N.A., Pletnev A.A., Kesharwani T., Raminelli C., Yao T., Zeni G., Zhang L., Rozhkov R., Larock R.C. (2018). Comprehensive Organic Transformations, 4 Volume Set: A Guide to Functional Group Preparations.

[8] Ishihara K. (2009). Dehydrative condensation catalyses. Tetrahedron.

[9] Sakakura A., Koshikari Y., Ishihara K. (2008). Open-air and solvent-free ester condensation catalyzed by sulfonic acids. Tetrahedron Lett..

[10] Huang Y.-B., Yang T., Cai B., Chang X., Pan H. (2016). Highly efficient metal salt catalyst for the esterification of biomass derived levulinic acid under microwave irradiation. RSC Adv..

[11] Jeschke J., Korb M., Rüffer T., Gäbler C., Lang H. (2015). Atom economic ruthenium-catalyzed synthesis of bulky β-Oxo esters. Adv. Synth. Catal..

[12] Da Silva M.J., Liberto N.A., De Andrade Leles L.C., Pereira U.A. (2016). Fe_4_(SiW_12_O_40_)_3_-catalyzed glycerol acetylation: Synthesis of bioadditives by using highly active Lewis acid catalyst. J. Mol. Catal. A Chem..

[13] Kato C.N., Ogasawara T., Kondo A., Kato D. (2017). Heterogeneous esterification of fatty acids with methanol catalyzed by Lewis acidic organozirconium complexes with Keggin-type mono-aluminum-substituted polyoxotungstates. Catal. Commun..

[14] Minakawa M., Baek H., Yamada Y.M.A., Han J.W., Uozumi Y. (2013). Direct dehydrative esterification of alcohols and carboxylic acids with a macroporous polymeric acid catalyst. Org. Lett..

[15] Dell’Anna M.M., Capodiferro V.F., Mali M., Mastrorilli P. (2016). Esterification, transesterification and hydrogenation reactions of polyunsaturated compounds catalyzed by a recyclable polymer supported palladium catalyst. J. Organomet. Chem..

[16] Furuta A., Fukuyama T., Ryu I. (2017). Efficient flow fischer esterification of carboxylic acids with alcohols using sulfonic acid-functionalized silica as supported catalyst. Bull. Chem. Soc. Jpn..

[17] Chen Z., Wen Y., Fu Y., Chen H., Ye M., Luo G. (2017). Graphene oxide: An efficient acid catalyst for the construction of esters from acids and alcohols. Synlett.

[18] Han X.-X., Du H., Hung C.-T., Liu L.-L., Wu P.-H., Ren D.-H., Huang S.-J., Liu S.-B. (2015). Syntheses of novel halogen-free Bronsted-Lewis acidic ionic liquid catalysts and their applications for synthesis of methyl caprylate. Green Chem..

[19] Dong B., Song H., Zhang W., He A., Yao S. (2016). Ionic liquids as heterogeneous and homogeneous catalysts for condensation and esterification reactions. Curr. Org. Chem..

[20] Phakhodee W., Duangkamol C., Pattarawarapan M. (2016). Ph_3_P-I_2_ mediated aryl esterification with a mechanistic insight. Tetrahedron Lett..

[21] Yeh W.K., Yang H.C., McCarthy J.R. (2011). Enzyme Technologies: Metagenomics, Evolution, Biocatalysis and Biosynthesis.

[22] Bezbradica D., Crovic M., Tanaskovic S.J., Lukovic N., Carevic M., Milivojevic A., Knezevic-Jugovic Z. (2017). Enzymatic Syntheses of Esters-Green Chemistry for Valuable Food, Fuel and Fine Chemicals. Curr. Org. Chem..

[23] Gokulakrishnan N., Pandurangan A., Sinha P.K. (2007). Esterification of acetic acid with propanol isomers under autogeneous pressure: A catalytic activity study of Al-MCM-41 molecular sieves. J. Mol. Catal. A Chem..

[24] Chung K.-H., Park B.-G. (2009). Esterification of oleic acid in soybean oil on zeolite catalysts with different acidity. J. Ind. Eng. Chem..

[25] Kolvari E., Ghorbani-Choghamarani A., Salehi P., Shirini F., Zolfigol M.A. (2007). Application of *N*-halo reagents in organic synthesis. J. Iran. Chem. Soc..

[26] Koval I.V. (2002). *N*-Halo Reagents. *N*-Halosuccinimides in organic synthesis and in chemistry of natural compounds. Russ. J. Org. Chem..

[27] Barton D., Ollis W.D. (1979). Comprehensive Organic Chemistry: The Synthesis and Reactions of Organic Compounds.

[28] Kadam S.T., Kim S.S. (2008). *N*-Iodosuccinimide (NIS) a novel and effective catalyst for the cyanosilylation of aldehydes under mild reaction conditions. Catal. Commun..

[29] Nagarajappa Giridhar B., Pandey Krishna K., Shinde Aniket S., Vagdevi Hosadu M. (2016). *N*-Bromosuccinimide (NBS)–An efficient catalyst for acetylation of wood. Holzforschung.

[30] Maleki B., Sedigh Ashrafi S. (2014). *N*-Bromosuccinimide catalyzed three component one-pot efficient synthesis of 2,4,5-Triaryl-1*H*-imidazoles from aldehyde, ammonium acetate, and 1,2-Diketone or ±-Hydroxyketone. J. Mex. Chem. Soc..

[31] Karimi B., Zamani A., Zareyee D. (2004). *N*-Iodosuccinimide (NIS) as a mild and highly chemoselective catalyst for deprotection of tert-butyldimethylsilyl ethers. Tetrahedron Lett..

[32] Saikia I., Borah A.J., Phukan P. (2016). Use of bromine and bromo-organic compounds in organic synthesis. Chem. Rev..

[33] Stavber G., Iskra J., Zupan M., Stavber S. (2008). Aerobic oxidative iodination of organic compounds with iodide catalyzed by sodium nitrite. Adv. Synth. Catal..

[34] Stavber G., Iskra J., Zupan M., Stavber S. (2009). Aerobic oxidative iodination of ketones catalysed by sodium nitrite "on water" or in a micelle-based aqueous system. Green Chem..

[35] Stavber G., Stavber S. (2010). Towards Greener Fluorine Organic Chemistry: Direct Electrophilic Fluorination of Carbonyl Compounds in Water and Under Solvent-Free Reaction Conditions. Adv. Synth. Catal..

[36] Prebil R., Stavber G., Stavber S. (2014). Aerobic oxidation of alcohols by using a completely metal-free catalytic system. Eur. J. Org. Chem..

[37] Ajvazi N., Stavber S. (2016). Direct halogenation of alcohols with halosilanes under catalyst- and organic solvent-free reaction conditions. Tetrahedron Lett..

[38] Ajvazi N., Stavber S. (2016). Transformation of tertiary benzyl alcohols into the vicinal halo-substituted derivatives using *N*-Halosuccinimides. Molecules.

[39] Vražič D., Jereb M., Laali K., Stavber S. (2013). Brønsted acidic ionic liquid accelerated halogenation of organic compounds with *N*-Halosuccinimides (NXS). Molecules.

[40] Podgoršek A., Stavber S., Zupan M., Iskra J. (2009). Environmentally benign electrophilic and radical bromination ‘on water’: H_2_O_2_–HBr system versus *N*-bromosuccinimide. Tetrahedron.

[41] Jereb M., Zupan M., Stavber S. (2009). Visible-light-promoted wohl–ziegler functionalization of organic molecules with *N*-Bromosuccinimide under solvent-free reaction conditions. Helv. Chim. Acta.

[42] Pravst I., Zupan M., Stavber S. (2008). Halogenation of ketones with *N*-halosuccinimides under solvent-free reaction conditions. Tetrahedron.

[43] Pravst I., Zupan M., Stavber S. (2006). Directed regioselectivity of bromination of ketones with NBS: Solvent-free conditions versus water. Tetrahedron Lett..

[44] Podgoršek A., Stavber S., Zupan M., Iskra J. (2006). Visible light induced ‘on water’ benzylic bromination with *N*-bromosuccinimide. Tetrahedron Lett..

[45] Pravst I., Zupan M., Stavber S. (2006). Solvent-free bromination of 1,3-diketones and *β*-keto esters with NBS. Green Chem..

[46] Bandgar B.P., Uppalla L.S., Sadavarte V.S. (2001). Chemoselective transesterification of *β*-Keto esters under neutral conditions using NBS as a catalyst. Synlett.

[47] Karimi B., Seradj H. (2001). *N*-Bromosuccinimide (NBS), a novel and highly effective catalyst for acetylation of alcohols under mild reaction conditions. Synlett.

[48] Sucheta K., Reddy G.S.R., Ravi D., Rama Rao N. (1994). A novel general route to the synthesis of carboxylic acid esters and thiolesters. Tetrahedron Lett..

[49] Ramalinga K., Vijayalakshmi P., Kaimal T.N.B. (2002). A mild and efficient method for esterification and transesterification catalyzed by iodine. Tetrahedron Lett..

[50] Jereb M., Vražič D., Zupan M. (2009). Dual behavior of alcohols in iodine-catalyzed esterification under solvent-free reaction conditions. Tetrahedron Lett..

[51] Jereb M., Vražič D., Zupan M. (2011). Iodine-catalyzed transformation of molecules containing oxygen functional groups. Tetrahedron.

[52] Vairamani M., Rao G.K.V. (1985). Use of bromine in methanol - preparation of methyl-esters. Indian J. Chem. Sect B.

[53] Bowman P.T., Ko E.I., Sides P.J. (1990). A potential hazard in preparing bromine-methanol solutions. J. Electrochem. Soc..

[54] Virtanen E., Kolehmainen E. (2004). Use of bile acids in pharmacological and supramolecular applications. Eur. J. Org. Chem..

[55] Cravotto G., Binello A., Boffa L., Rosati O., Boccalini M., Chimichi S. (2006). Regio- and stereoselective reductions of dehydrocholic acid. Steroids.

[56] Bose A., Mal P. (2014). Electrophilic aryl-halogenation using *N*-halosuccinimides under ball-milling. Tetrahedron Lett..

[57] Annese C., D'Accolti L., Fusco C., Licini G., Zonta C. (2017). Heterolytic (2 e) vs Homolytic (1 e) Oxidation reactivity: N−H versus C−H switch in the oxidation of lactams by Dioxirans. Chem. Eur. J..

[58] Nguyen T.V., Lyons D.J.M. (2015). A novel aromatic carbocation-based coupling reagent for esterification and amidation reactions. Chem. Commun..

[59] Shen G., Zhao L., Liu W., Huang X., Song H., Zhang T. (2017). Convenient, metal-free ipso-nitration of arylboronic acids using nitric acid and trifluoroacetic acid. Synth. Commun..

[60] Jia J., Jiang Q., Zhao A., Xu B., Liu Q., Luo W.-P., Guo C.-C. (2016). Copper-catalyzed O-methylation of carboxylic acids using DMSO as a methyl source. Synthesis.

[61] Powell A.B., Stahl S.S. (2013). Aerobic Oxidation of diverse primary alcohols to methyl esters with a readily accessible heterogeneous Pd/Bi/Te catalyst. Org. Lett..

[62] Cheung C.W., Buchwald S.L. (2013). Mild and General palladium-catalyzed synthesis of methyl aryl ethers enabled by the use of a palladacycle precatalyst. Org. Lett..

[63] Stephens M.D., Yodsanit N., Melander C. (2016). Potentiation of the fosmidomycin analogue FR 900098 with substituted 2-oxazolines against Francisella novicida. MedChemComm.

[64] Offermann D.A., McKendrick J.E., Sejberg J.J.P., Mo B., Holdom M.D., Helm B.A., Leatherbarrow R.J., Beavil A.J., Sutton B.J., Spivey A.C. (2012). Synthesis and incorporation into cyclic peptides of tolan amino acids and their hydrogenated congeners: construction of an array of A–B-loop mimetics of the Cε3 domain of human IgE. J. Org. Chem..

[65] Strazzolini P., Gambi A.G., Giumanini A., Vancik H. (1998). The reaction between ethanedioyl (oxalyl) dihalides and Ag_2_C_2_O_4_: A route to Staudinger's elusive ethanedioic (oxalic) acid anhydride. J. Chem. Soc. Perkin Trans..

[66] Sun H.-B., Hua R., Yin Y. (2006). ZrOCl_2_·8H_2_O: An efficient, cheap and reusable catalyst for the esterification of acrylic acid and other carboxylic acids with equimolar amounts of alcohols. Molecules.

[67] Maslov M.A., Morozova N.G., Solomatina T.V., Shaforostova N.G., Serebrennikova G.A. (2011). Synthesis of amino analogues of cholic acid. Russ. J. Bioorg. Chem..

[68] Rohacova J., Marin M.L., Martinez-Romero A., O'Connor J.-E., Gomez-Lechon M.J., Donato M.T., Castell J.V., Miranda M.A. (2009). Synthesis of new, UV-photoactive dansyl derivatives for flow cytometric studies on bile acid uptake. Org. Biomol. Chem..

[69] Hutchby M., Houlden C.E., Haddow M.F., Tyler S.N.G., Lloyd-Jones G.C., Booker-Milburn K.I. (2012). Switching pathways: room-temperature neutral solvolysis and substitution of amides. Angew. Chem. Int. Ed..

[70] Li G., Zatolochnaya O.V., Wang X.-J., Rodríguez S., Qu B., Desrosiers J.-N., Mangunuru H.P.R., Biswas S., Rivalti D., Karyakarte S.D., Sieber J.D., Grinberg N., Wu L., Lee H., Haddad N., Fandrick D.R., Yee N.K., Song J.J., Senanayake C.H. (2018). BABIPhos family of biaryl dihydrobenzooxaphosphole ligands for asymmetric hydrogenation. Org. Lett..

[71] Dethe D.H., Erande R.D., Ranjan A. (2013). Biomimetic total syntheses of borreverine and flinderole alkaloids. J. Org. Chem..

[72] Lai L., Li A.N., Zhou J., Guo Y., Lin L., Chen W., Wang R. (2017). Mg(OMe)_2_ promoted allylic isomerization of γ-hydroxy-α,β-alkenoic esters to synthesize γ-ketone esters. Org. Biomol. Chem..

[73] Suwada M., Fukuhara T., Hara S. (2007). Selective mono-fluorination of diols via a cyclic acetal of *N*,*N*-diethyl-4-methoxybenzamide. J. Fluorine Chem..

[74] Jiang Q., Zhao A., Xu B., Jia J., Liu X., Guo C. (2014). PIFA-Mediated Esterification Reaction of Alkynes with Alcohols via Oxidative Cleavage of Carbon Triple Bonds. J. Org. Chem..

[75] Watanabe S., Fujita T., Sakamoto M., Kuramochi T., Kitazume T. (1987). Reactions of monoesters of ethylene glycol with *N*,*N*-diethyl-1,1,2,3,3,3-hexafluoropropylamine. J. Fluorine Chem..

[76] Umeda R., Nishimura T., Kaiba K., Tanaka T., Takahashi Y., Nishiyama Y. (2011). Rhenium complex-catalyzed acylative cleavage of ethers with acyl chlorides. Tetrahedron.

[77] Whittaker A.M., Dong V.M. (2015). Nickel-catalyzed dehydrogenative cross-coupling: direct transformation of aldehydes into esters and amides. Angew. Chem. Int. Ed..

[78] Gianetti T.L., Annen S.P., Santiso-Quinones G., Reiher M., Driess M., Grützmacher H. (2016). Nitrous Oxide as a Hydrogen Acceptor for the Dehydrogenative Coupling of Alcohols. Angew. Chem. Int. Ed..

[79] Kaul S., Kumar A., Sain B., Gupta A.K. (2002). A simple and convenient one-pot synthesis of fatty acid esters from hindered alcohols using *N*,*N*-dimethylchloro-sulfitemethaniminium chloride as dehydrating agent. Synth. Commun..

