# Supplementary material for: Esterification of Aryl/Alkyl Acids Catalysed by N-bromosuccinimide under Mild Reaction Conditions
Source: Molecules. 2018 Sep 2;23(9):2235. doi: 10.3390/molecules23092235 (PMC6225170; doi:10.3390/molecules23092235)

Supporting Information

Esterification of aryl/alkyl acids catalysed by *N*-bromosuccinimide under mild reaction conditions

Klara Čebular[1,2], Bojan Đ. Božić[3,4], Stojan Stavber[1,2,4*]

[1] Centre of Excellence for Integrated Approaches in Chemistry and Biology of Proteins, Jamova 39, 1000 Ljubljana, Slovenia
[2] Jožef Stefan International Postgraduate School, Jamova 39, 1000 Ljubljana, Slovenia
[3] Institute of Physiology and Biochemistry, Faculty of Biology, University of Belgrade, Studentski trg 3, 11000 Belgrade, Serbia
[4] Department of Physical and Organic Chemistry, Jožef Stefan Institute, Jamova 39, 1000 Ljubljana, Slovenia
[*]*Corresponding author:* e-mail: stojan.stavber@ijs.si; tel.: +38614773660; fax: +38614235400

A. Copies of ^{1}H, ^{13}C and ^{19}F NMR spectra 2

A. Copies of ^{1}H, ^{13}C and ^{19}F NMR spectra

Methyl benzoate (1a)

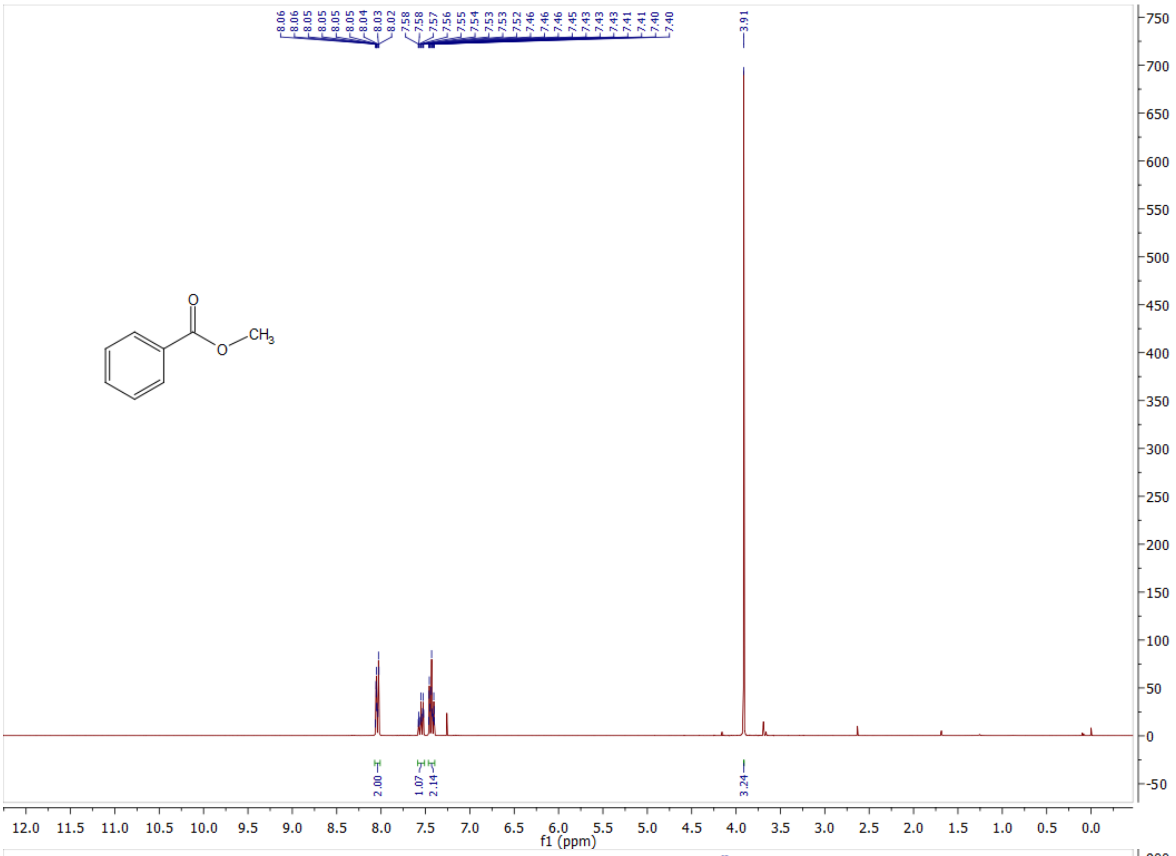

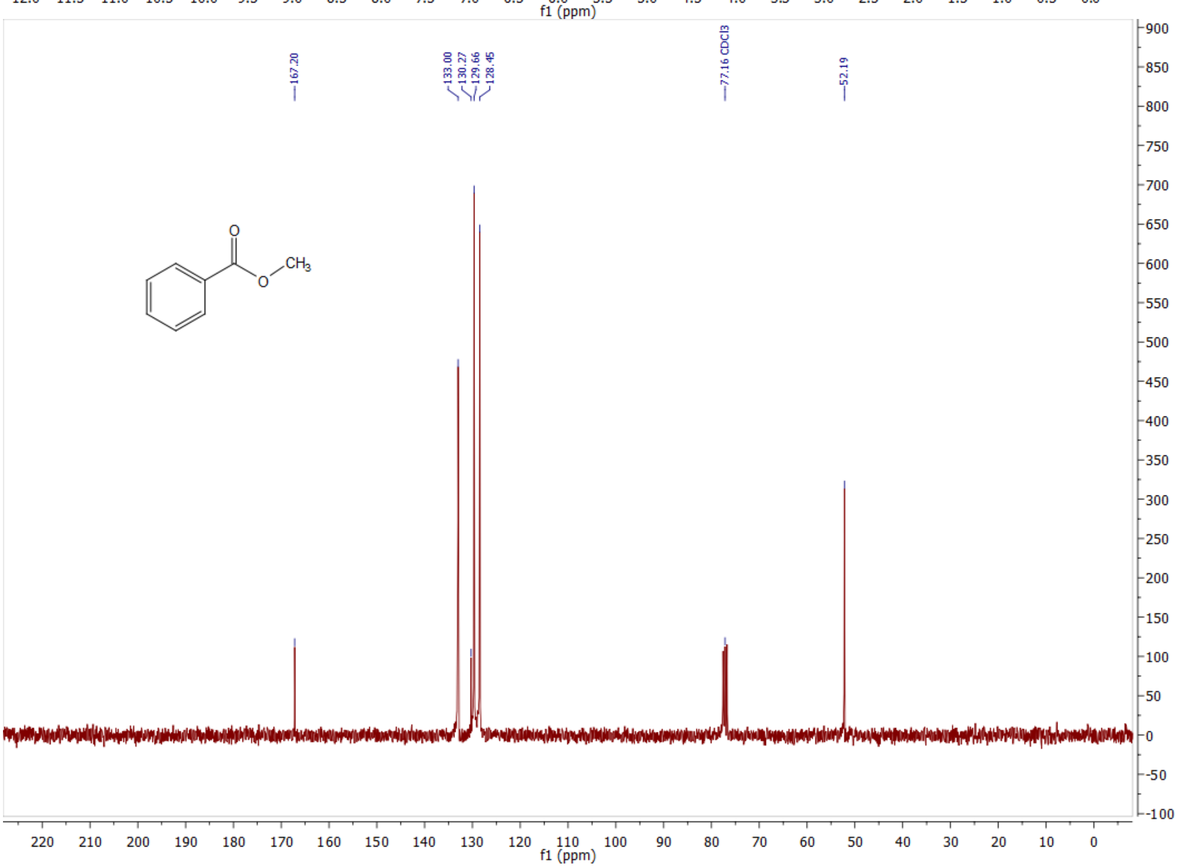

Methyl octanoate (2a)

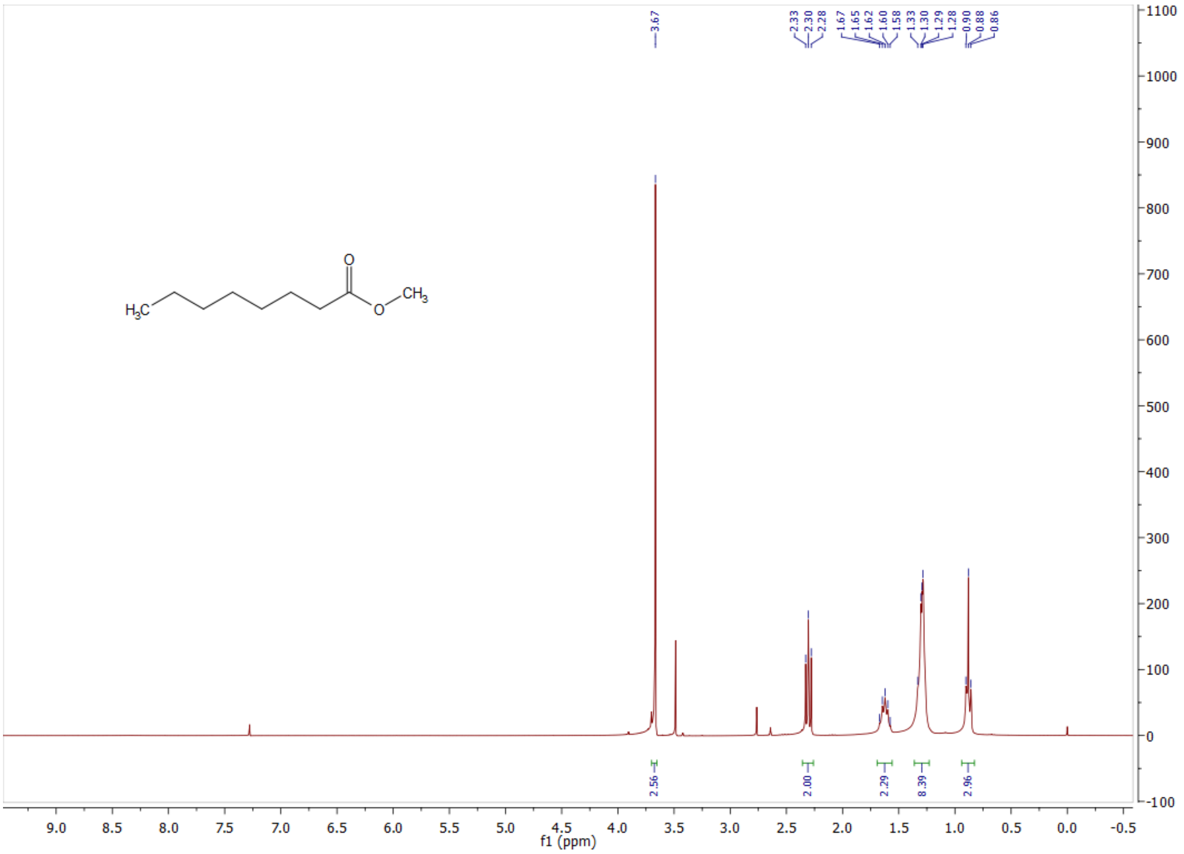

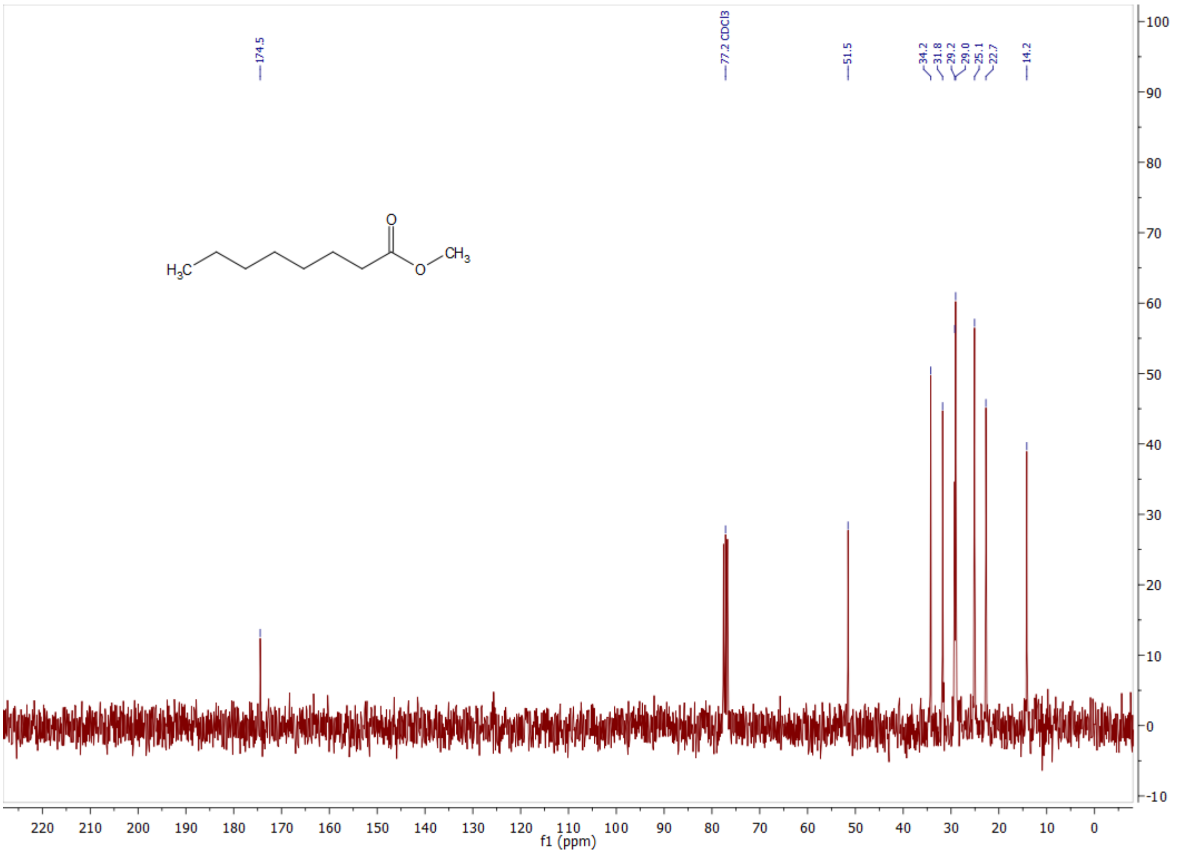

Methyl 4-nitrobenzoate (3a)

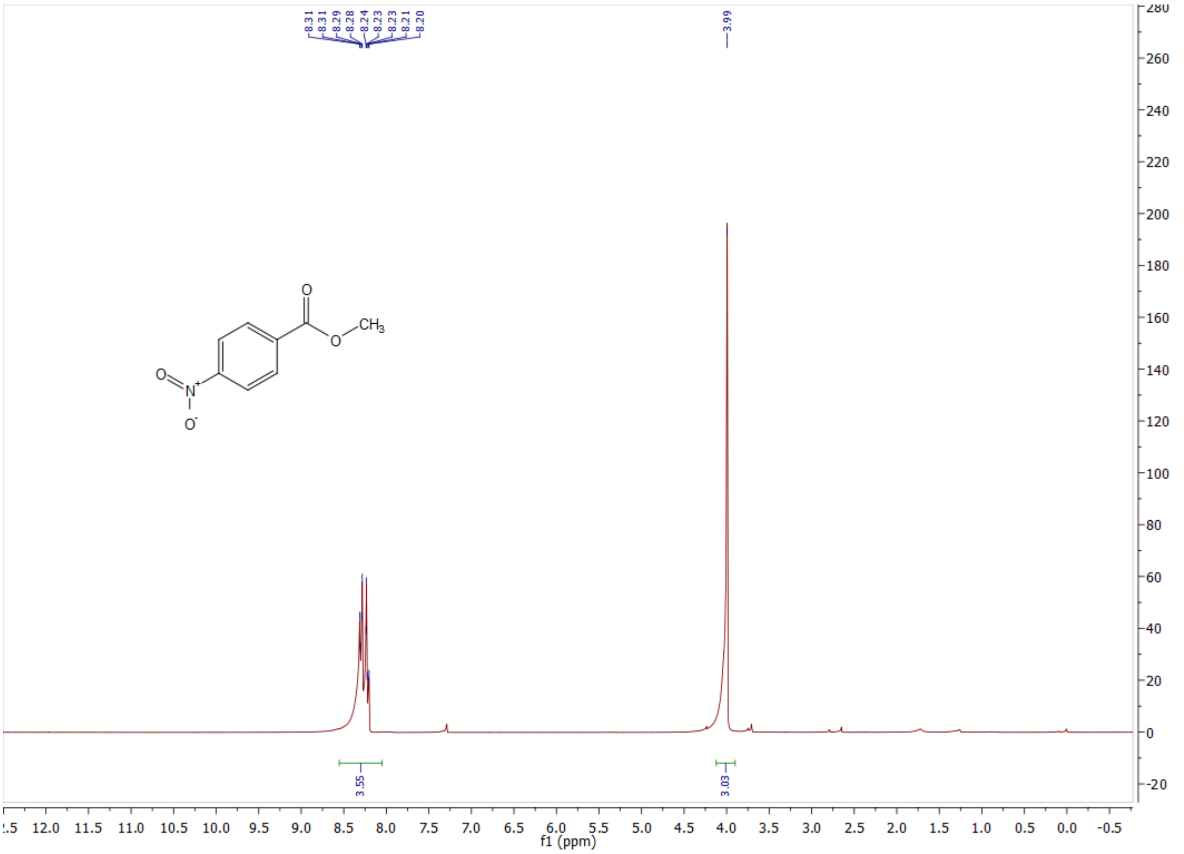

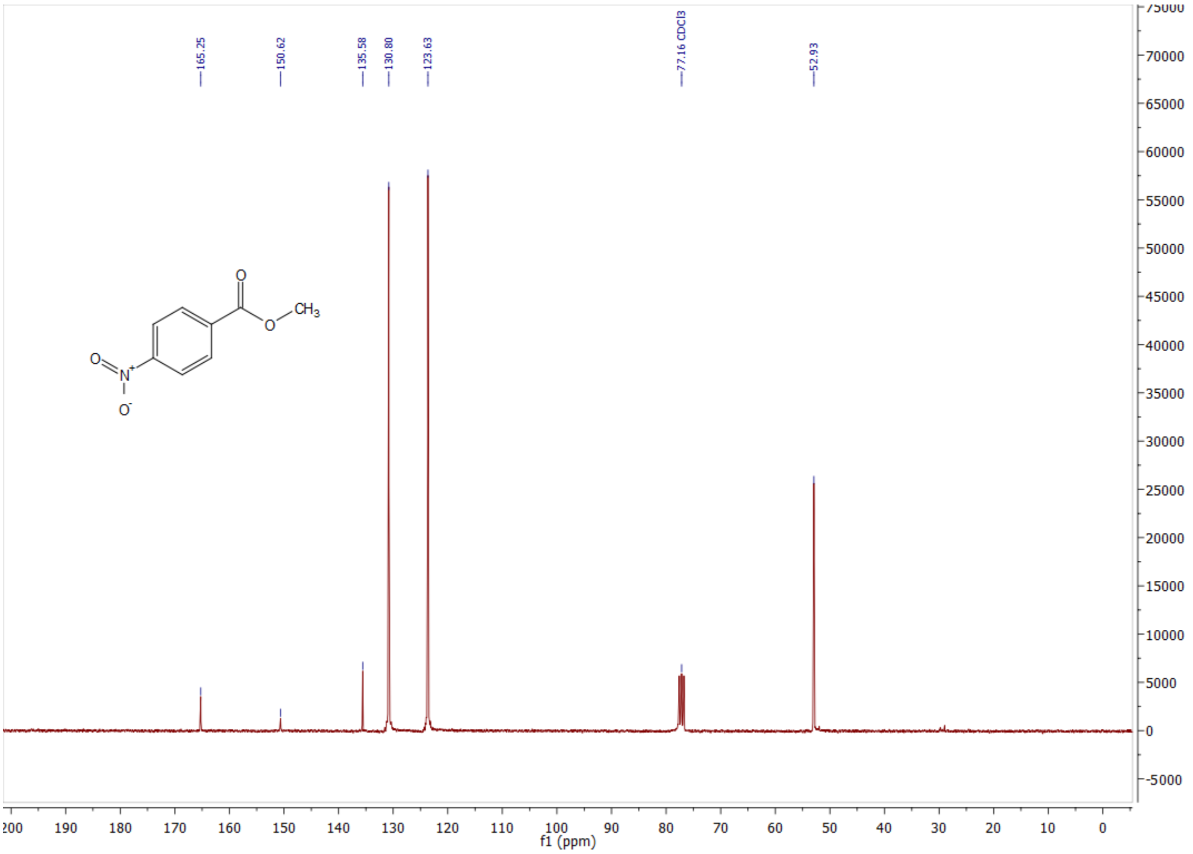

Methyl 3-nitrobenzoate (4a)

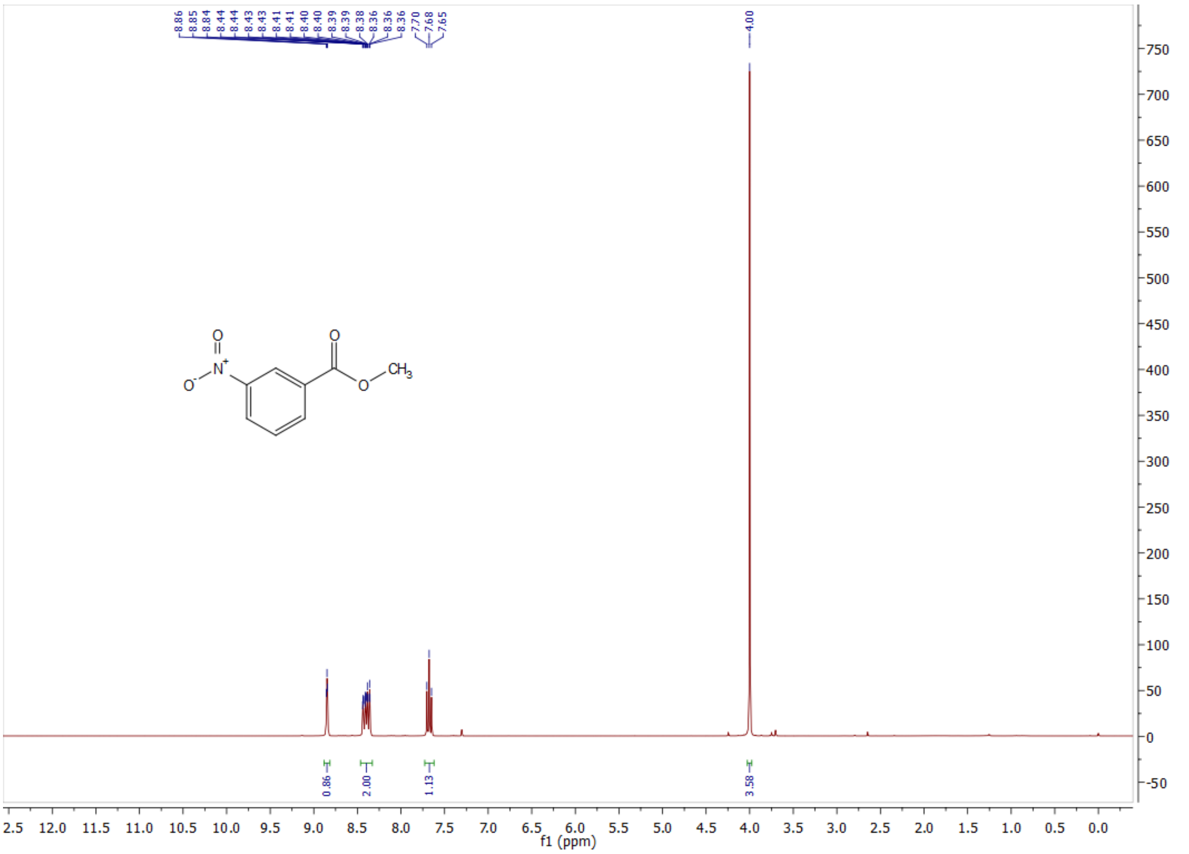

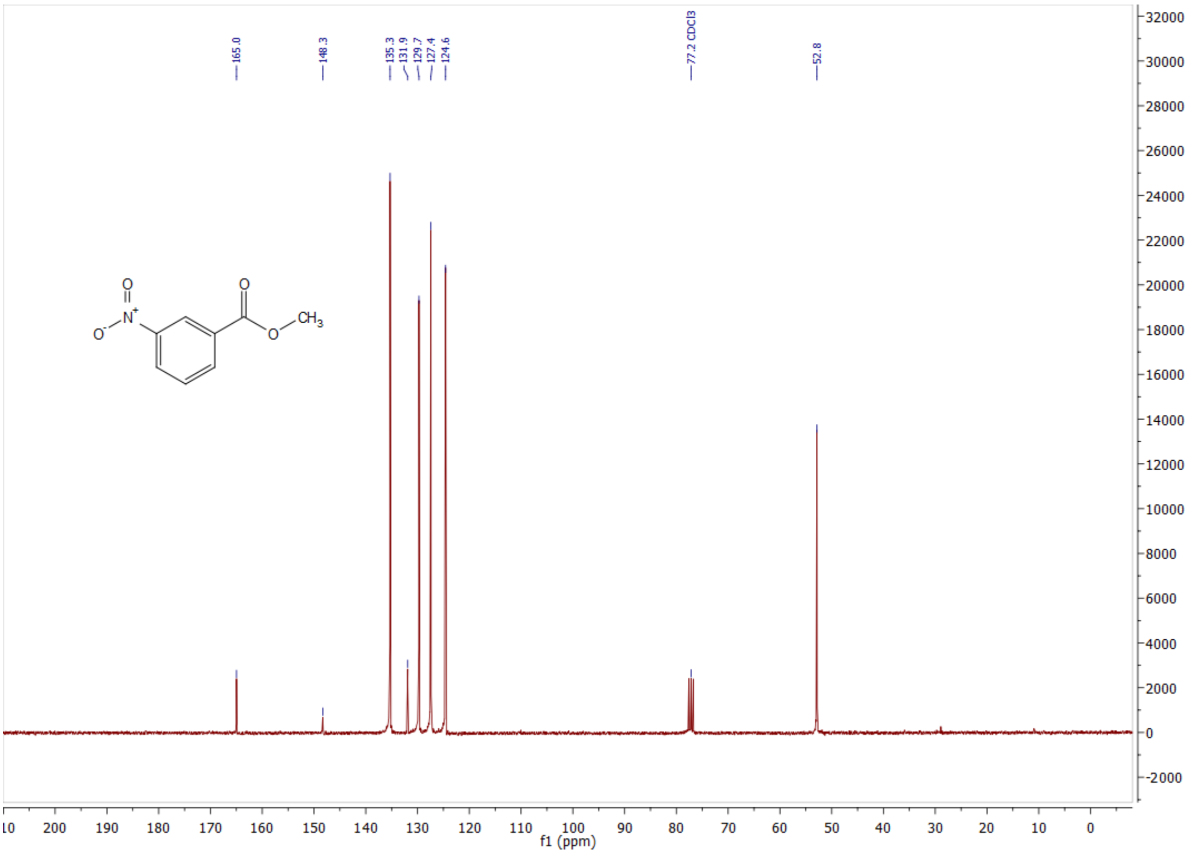

Methyl 4-fluorobenzoate (5a)

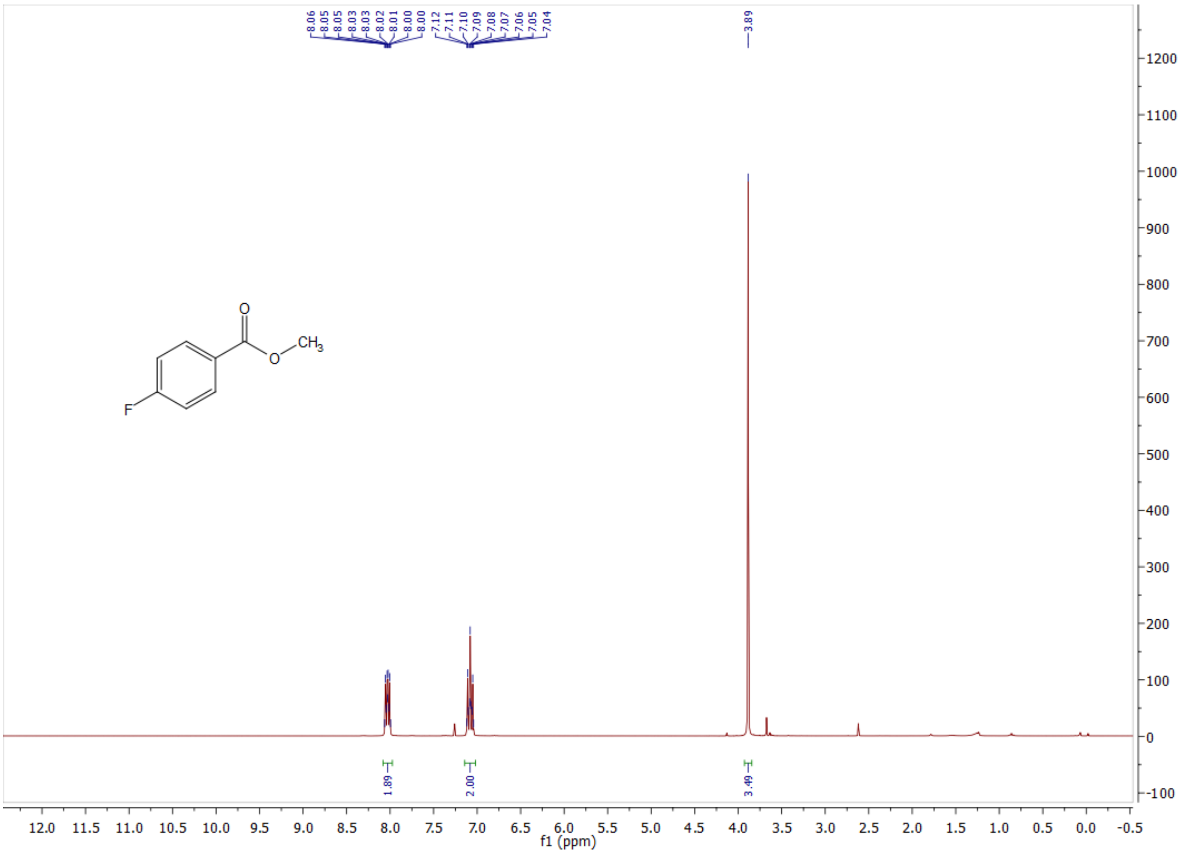

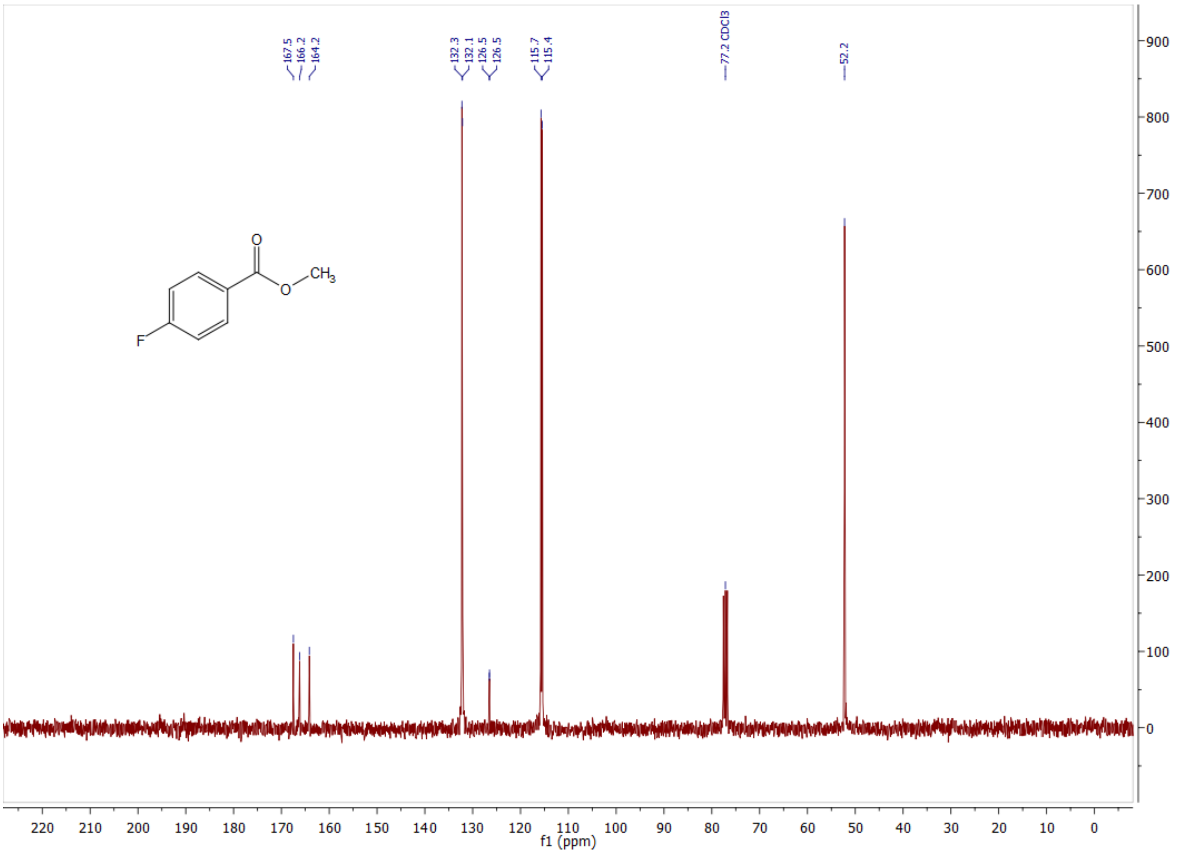

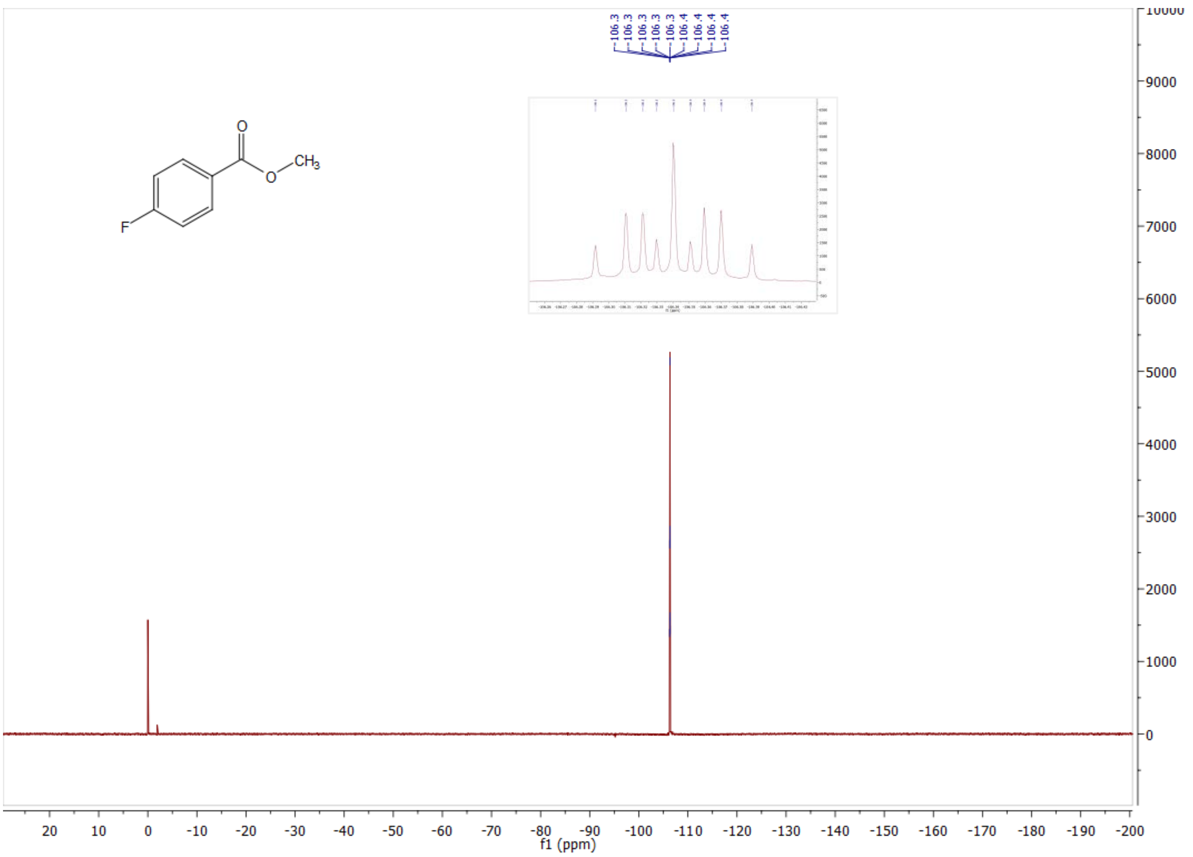

O
O
CH3
F
-106.3
-106.3
-106.3
-106.3
-106.3
-106.4
-106.4
-106.4
-106.4
f1 (ppm)

Methyl 3-fluorobenzoate (6a)

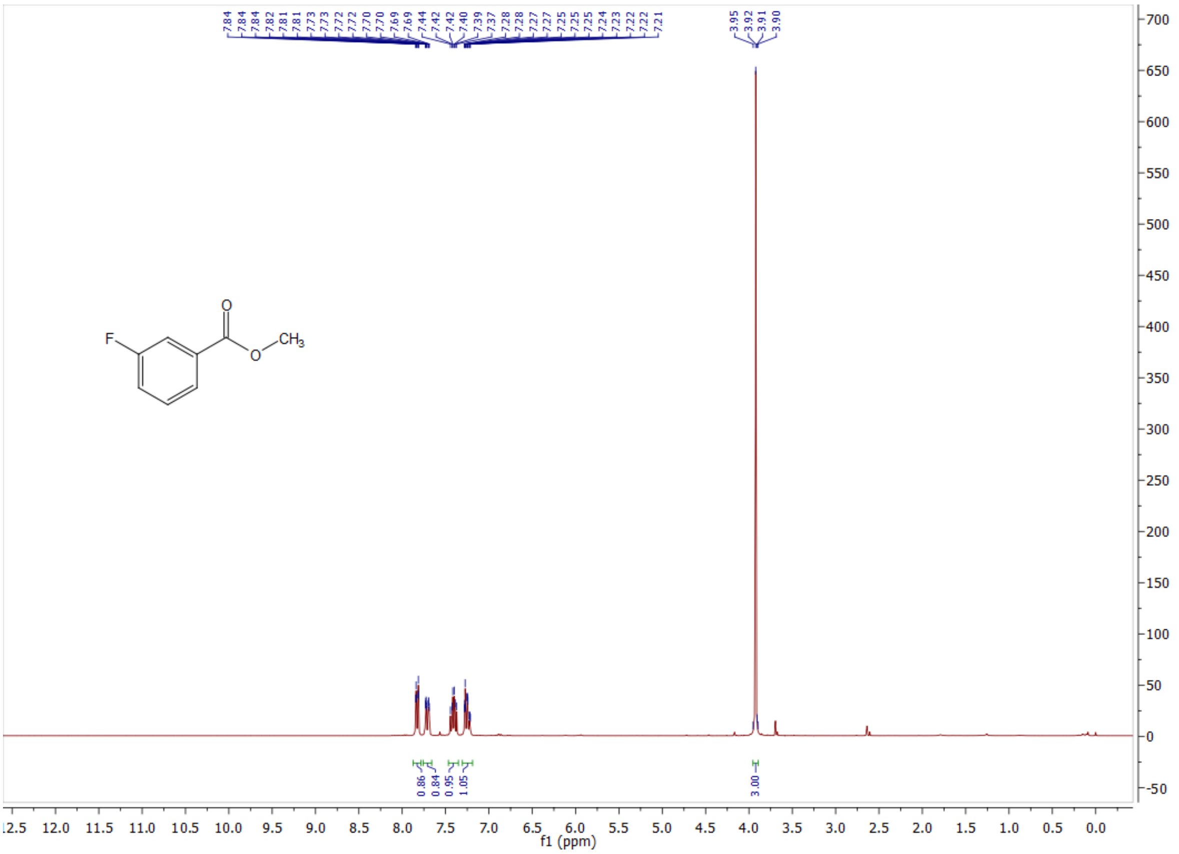

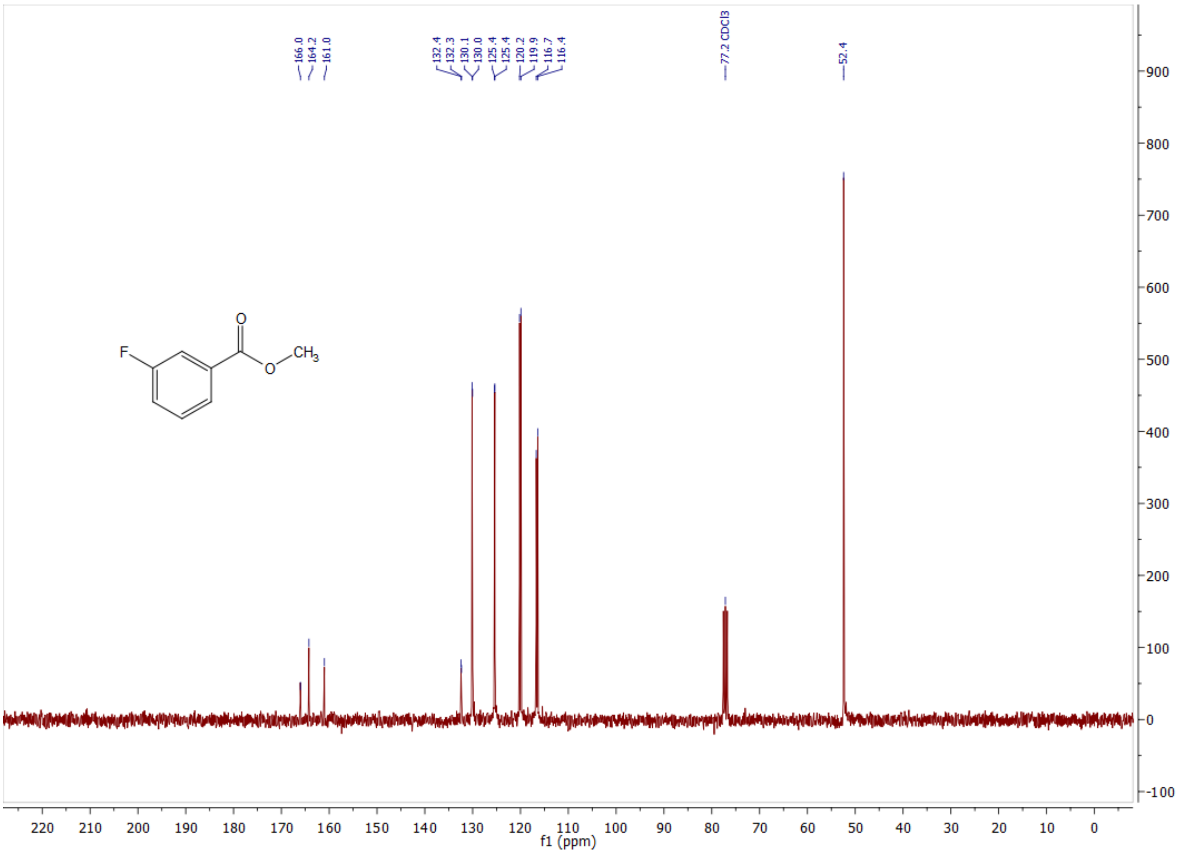

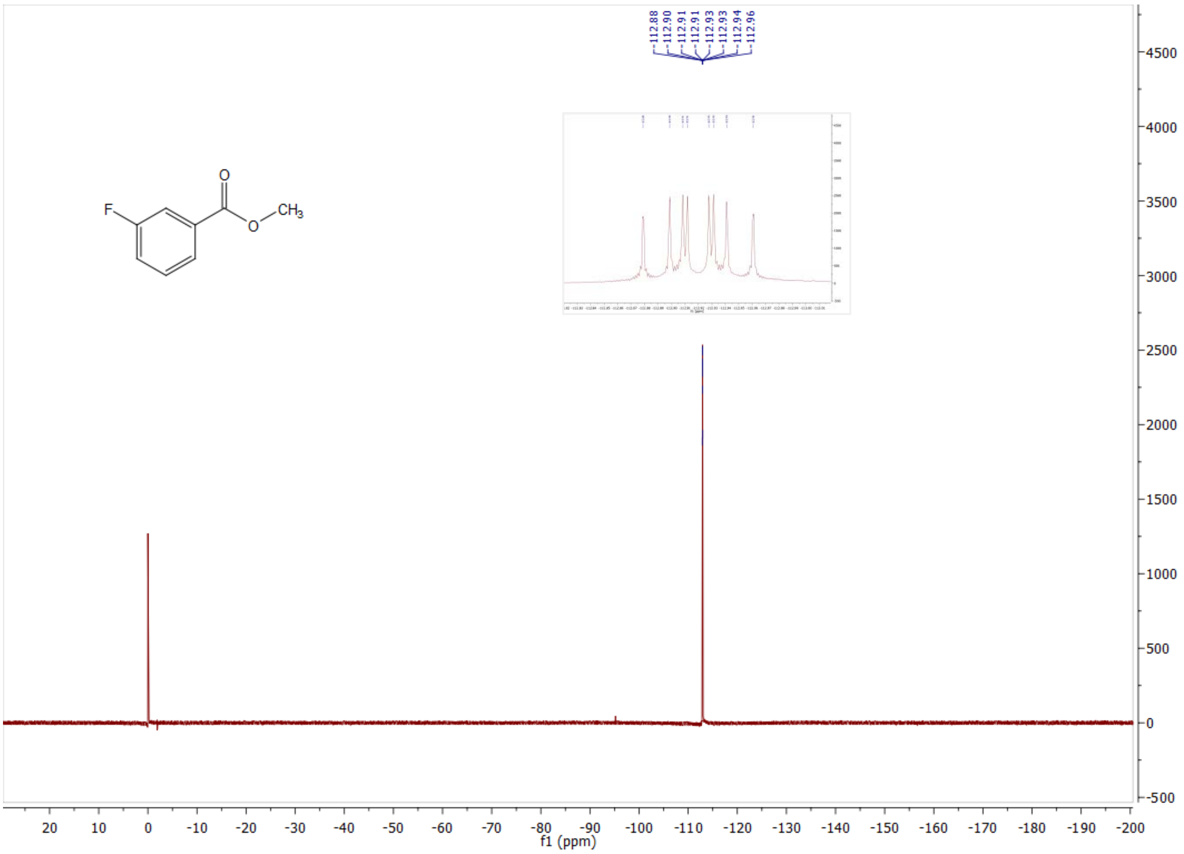

-112.88
-112.90
-112.91
-112.91
-112.93
-112.93
-112.94
-112.96
f1 (ppm)

Methyl 4-methylbenzoate (7a)

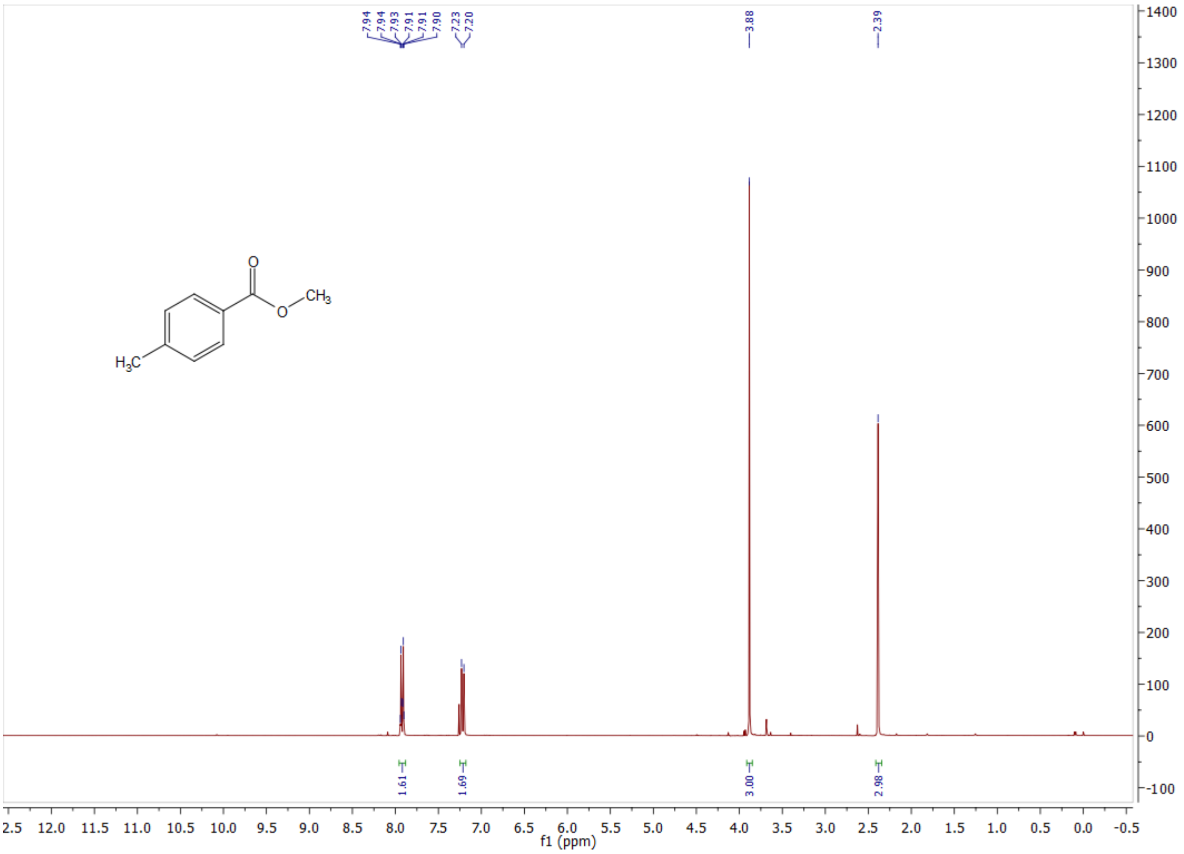

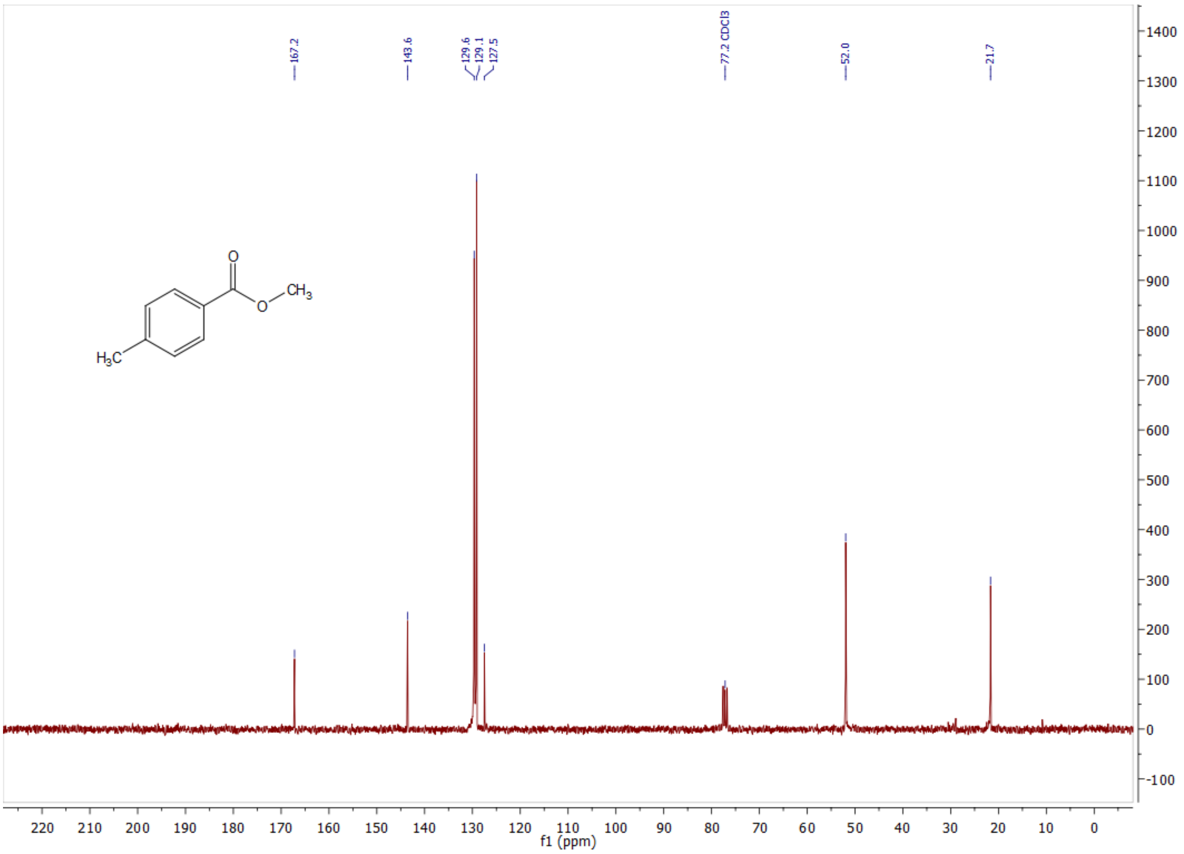

Methyl 3-methylbenzoate (8a)

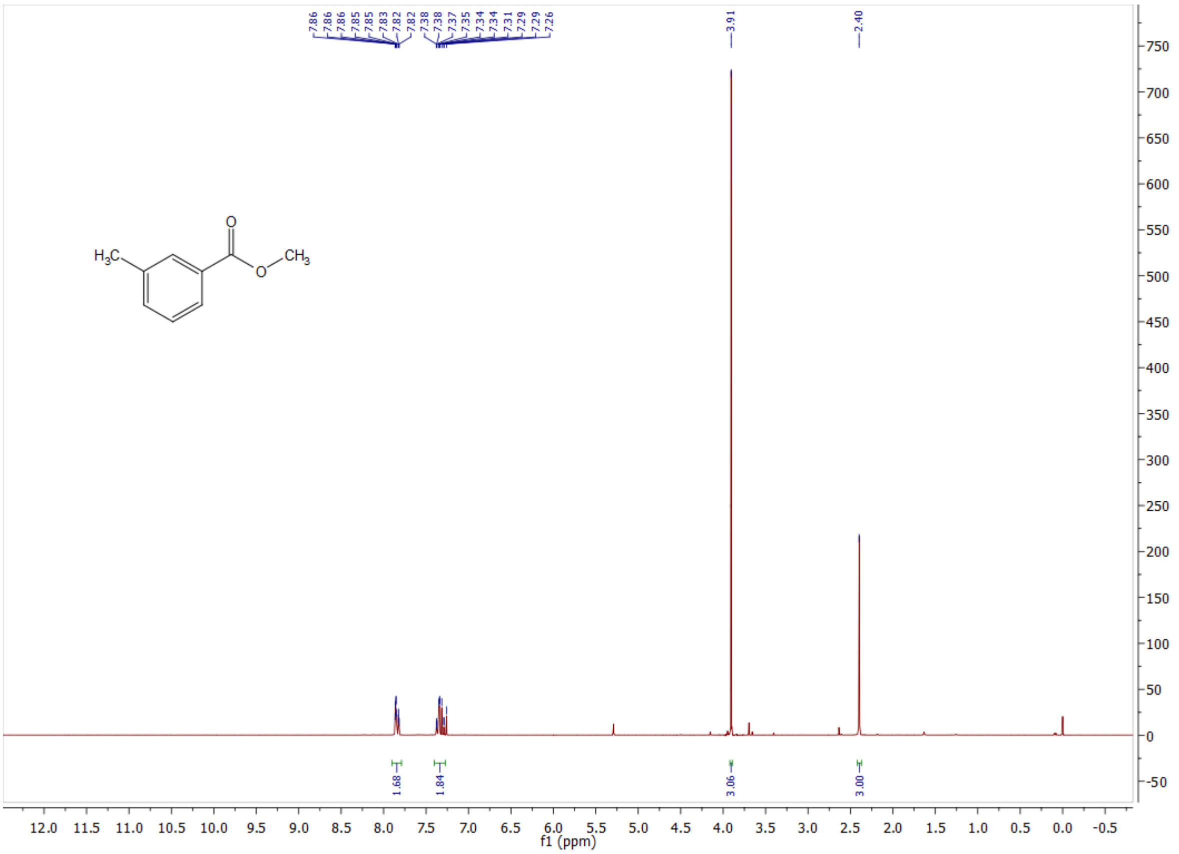

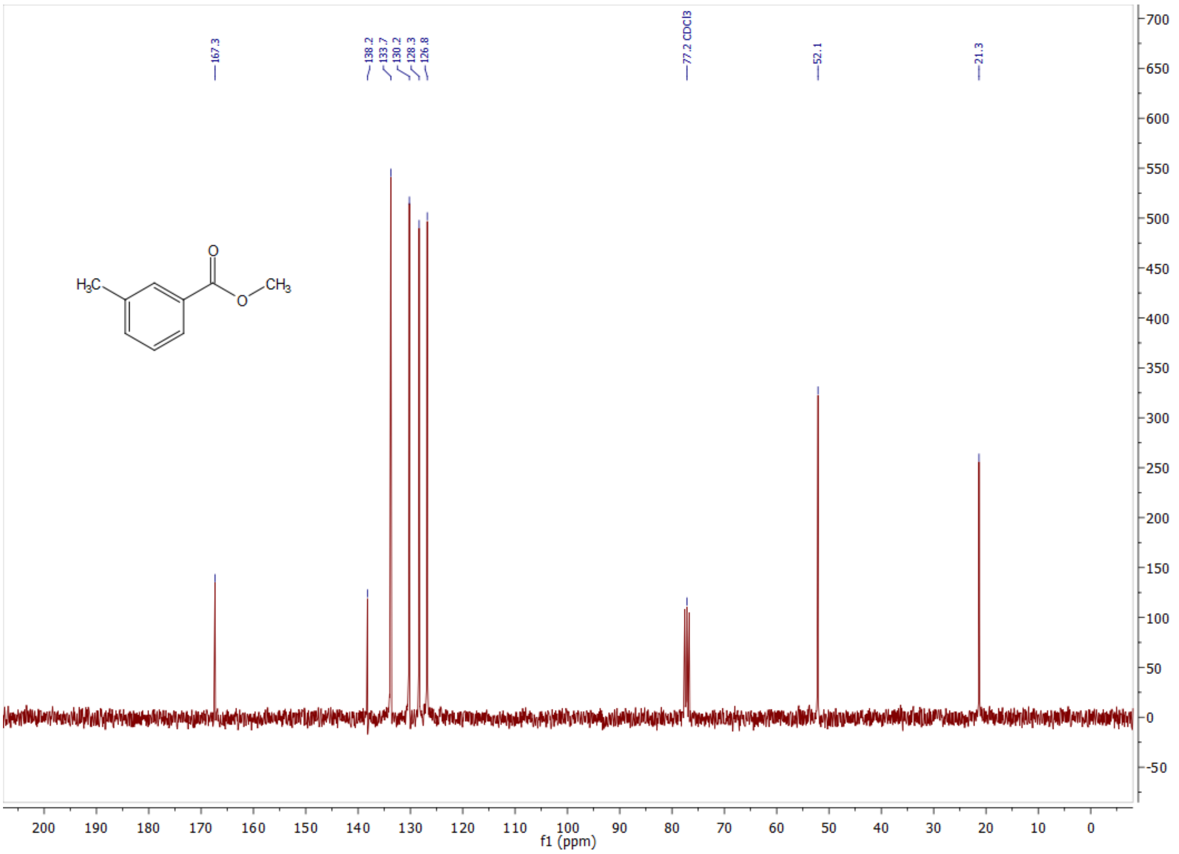

Methyl 3-methoxybenzoate (10a)

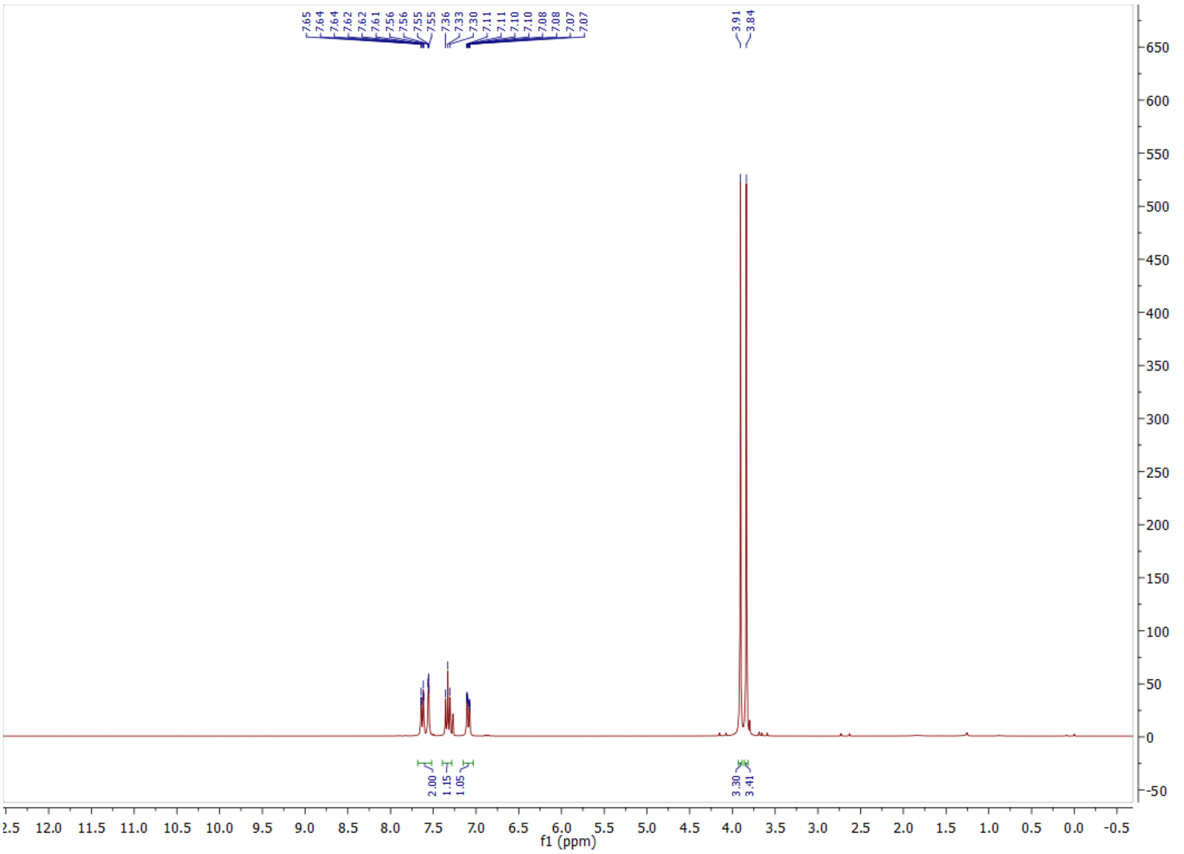

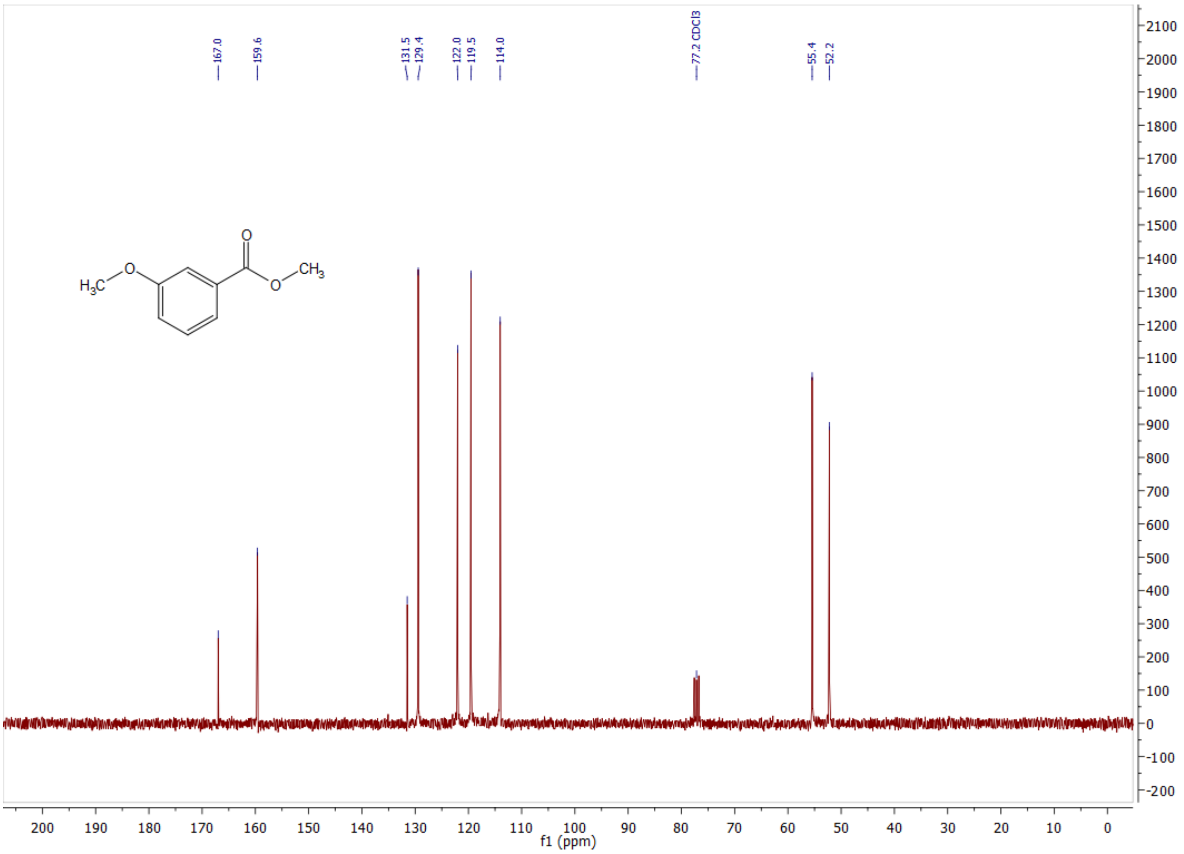

Methyl 4-octylbenzoate (11a)

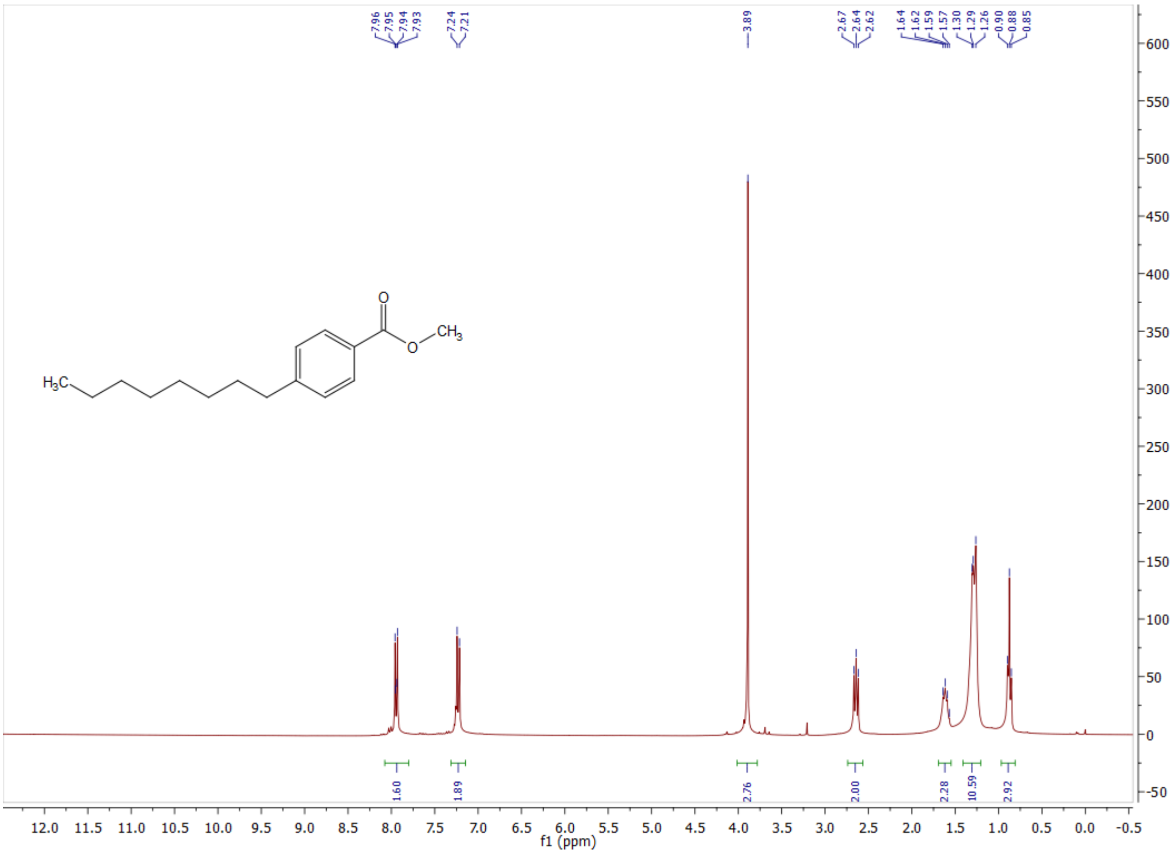

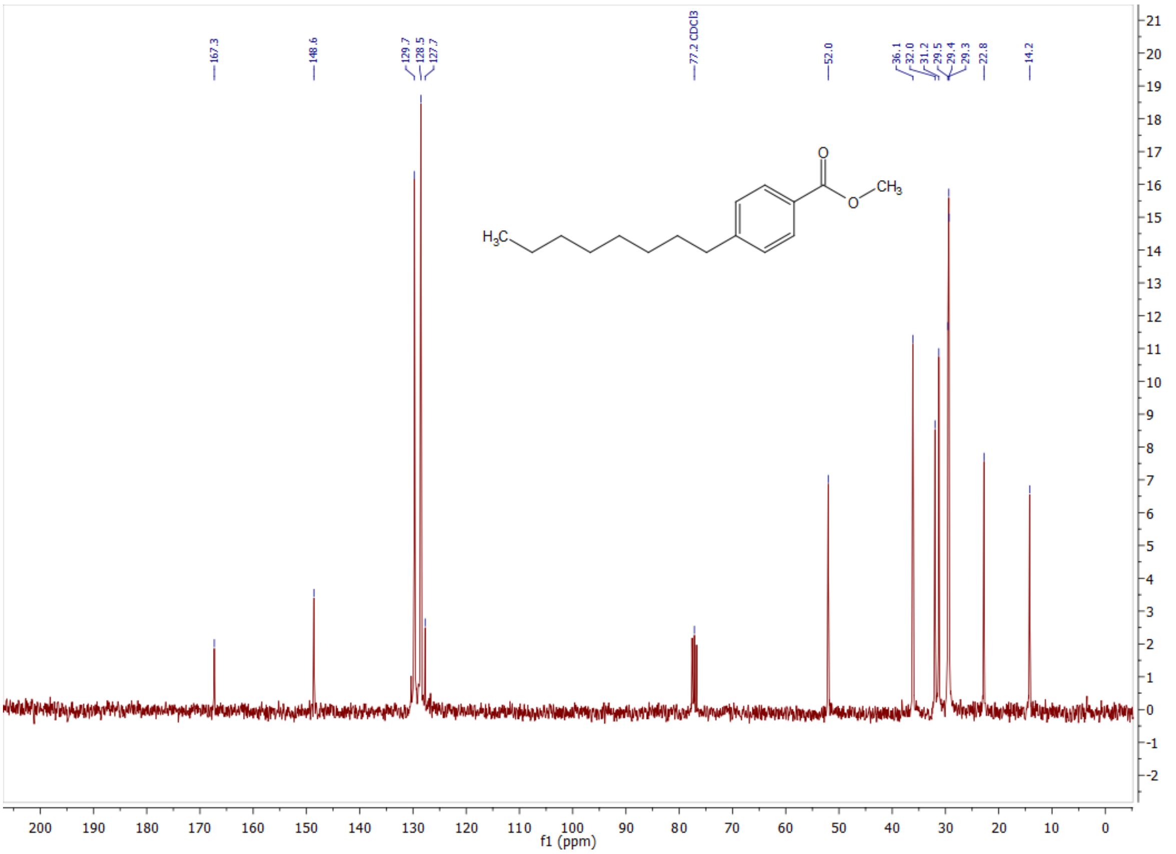

Methyl 2-iodobenzoate (12a)

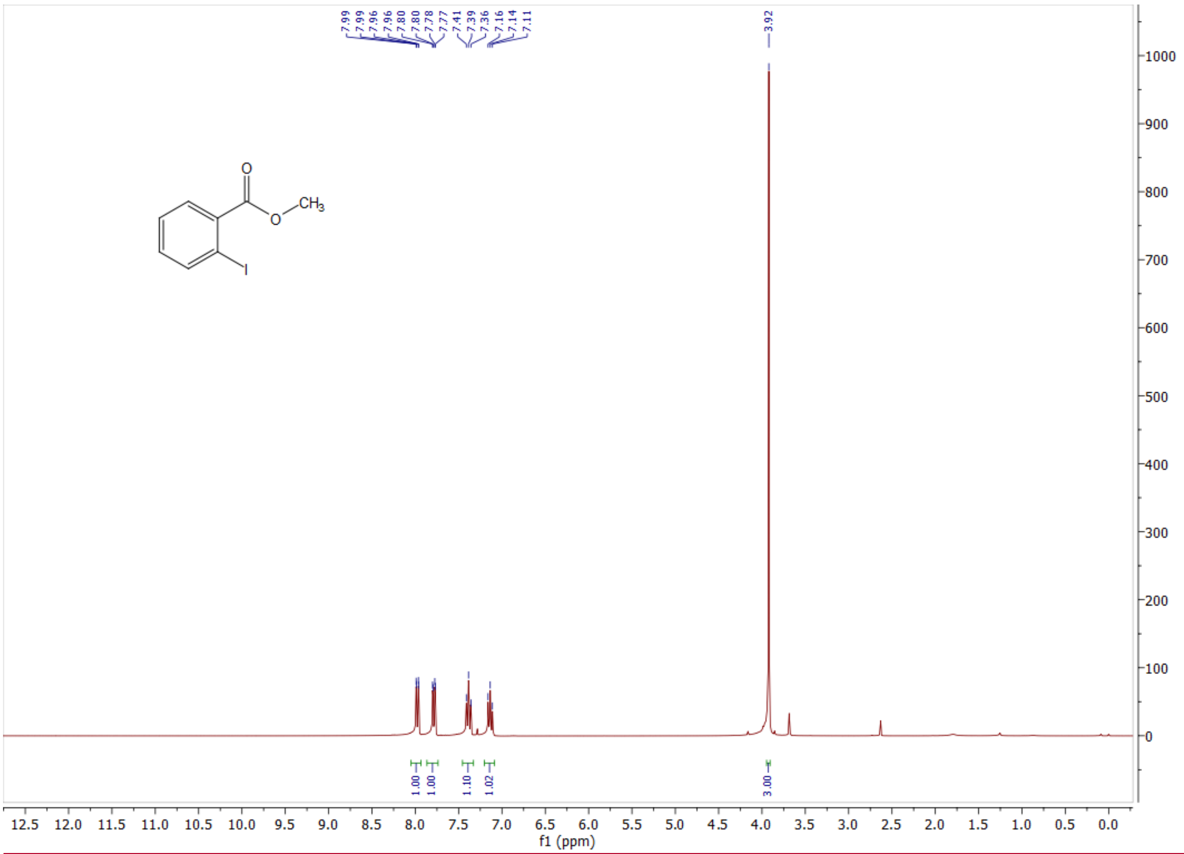

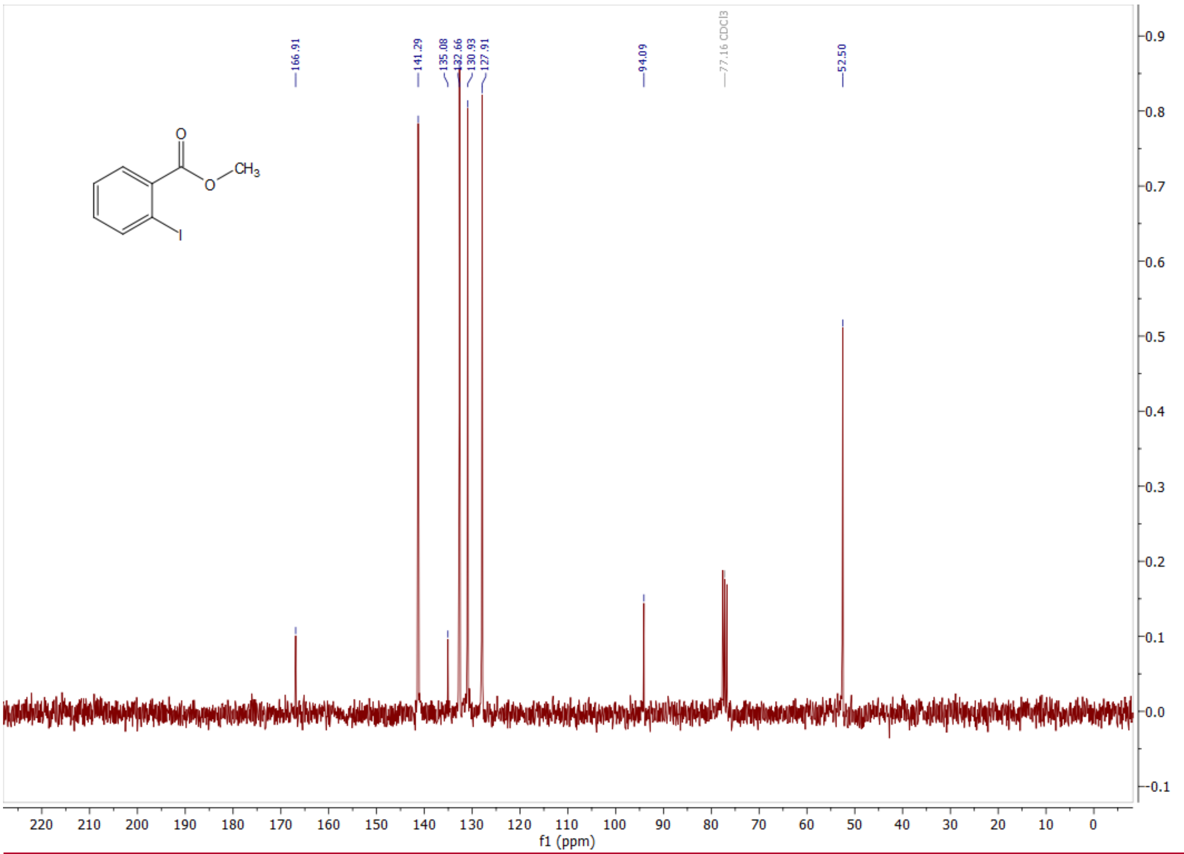

Methyl stearate (~~12a~~13a)

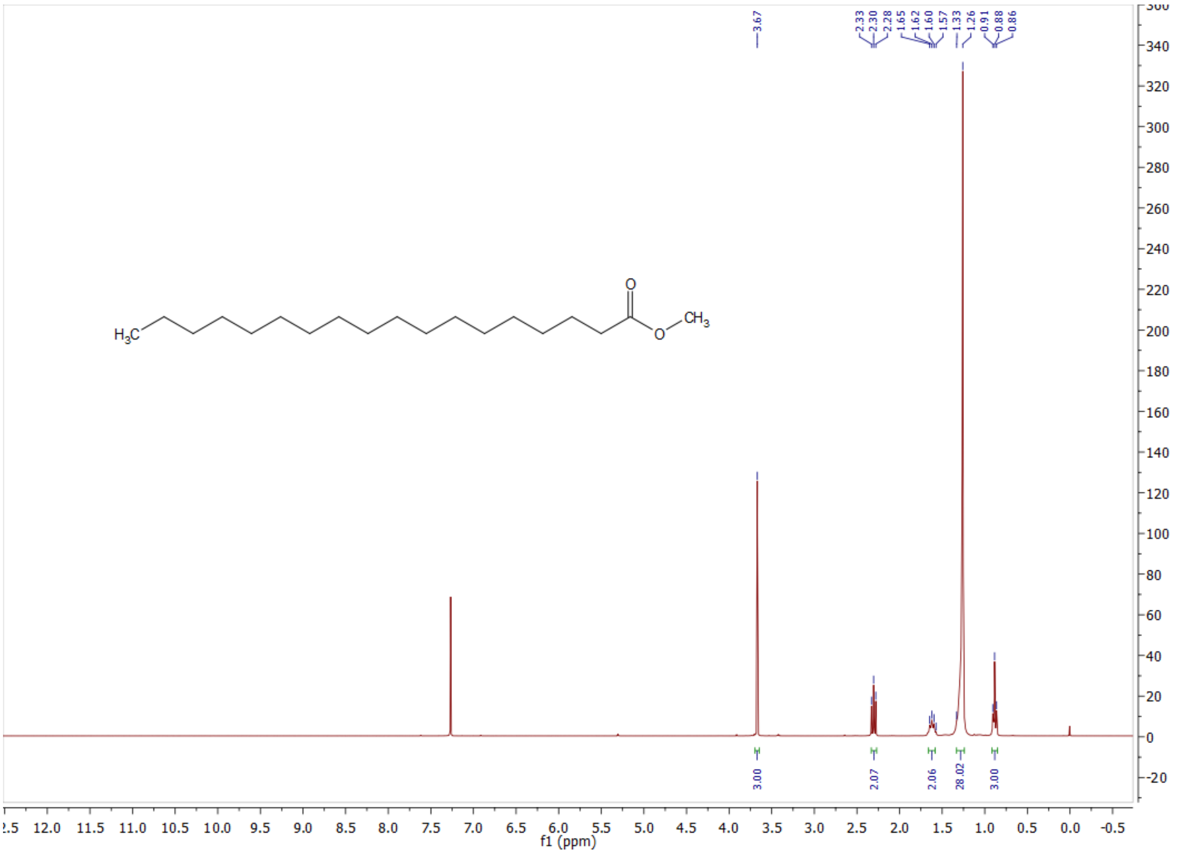

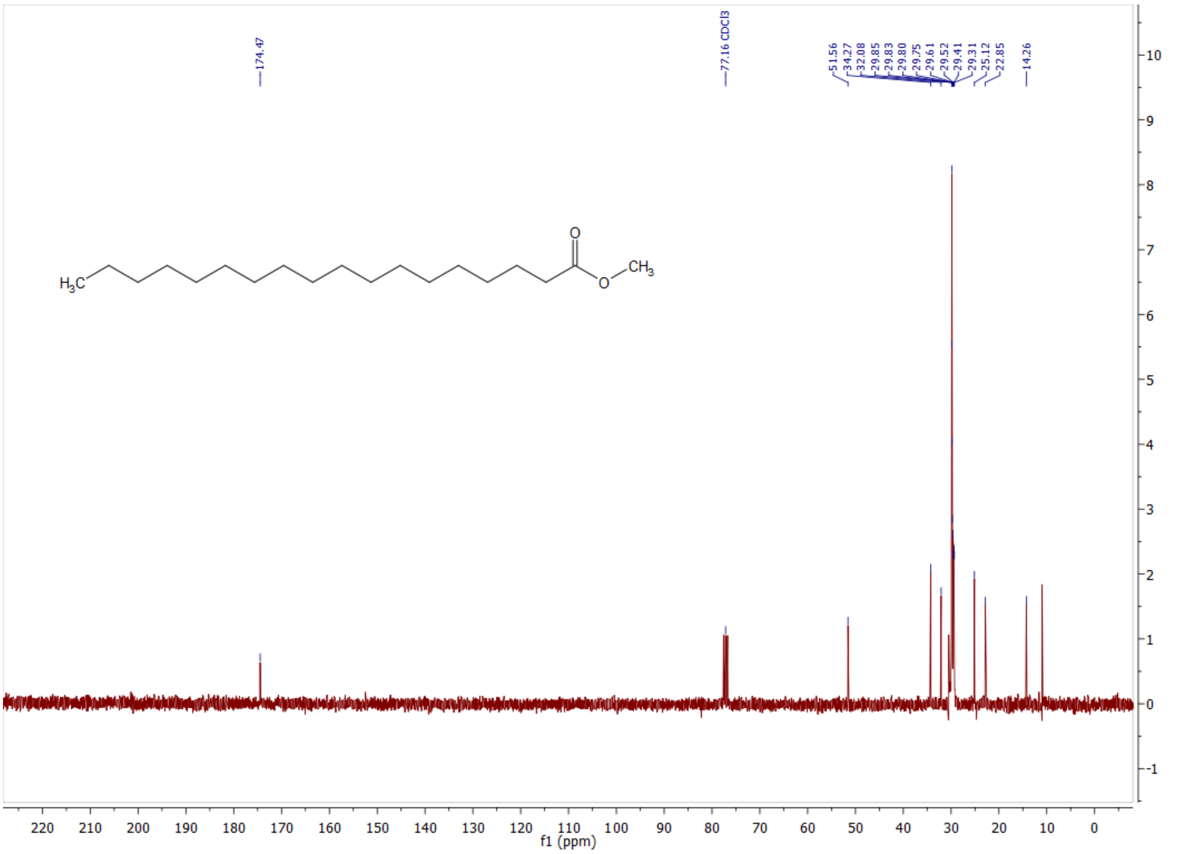

Methyl oleate (~~13a~~14a)

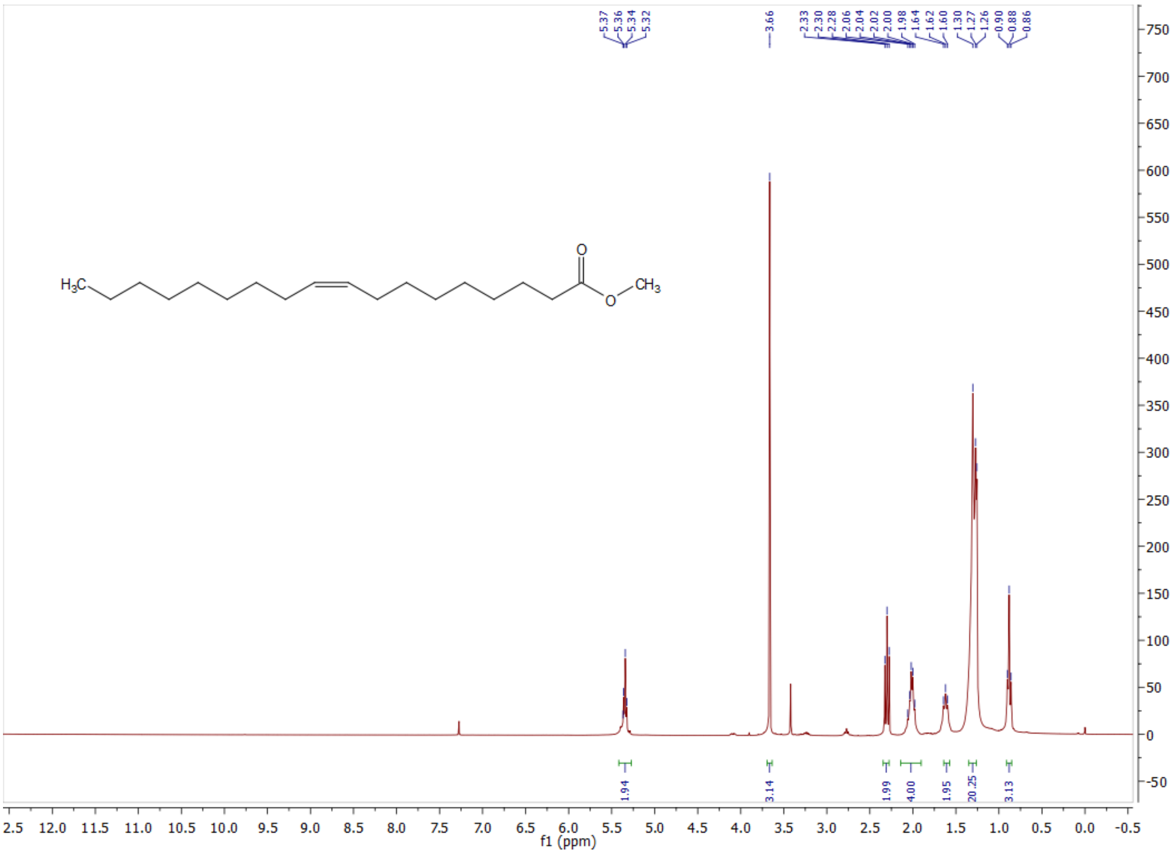

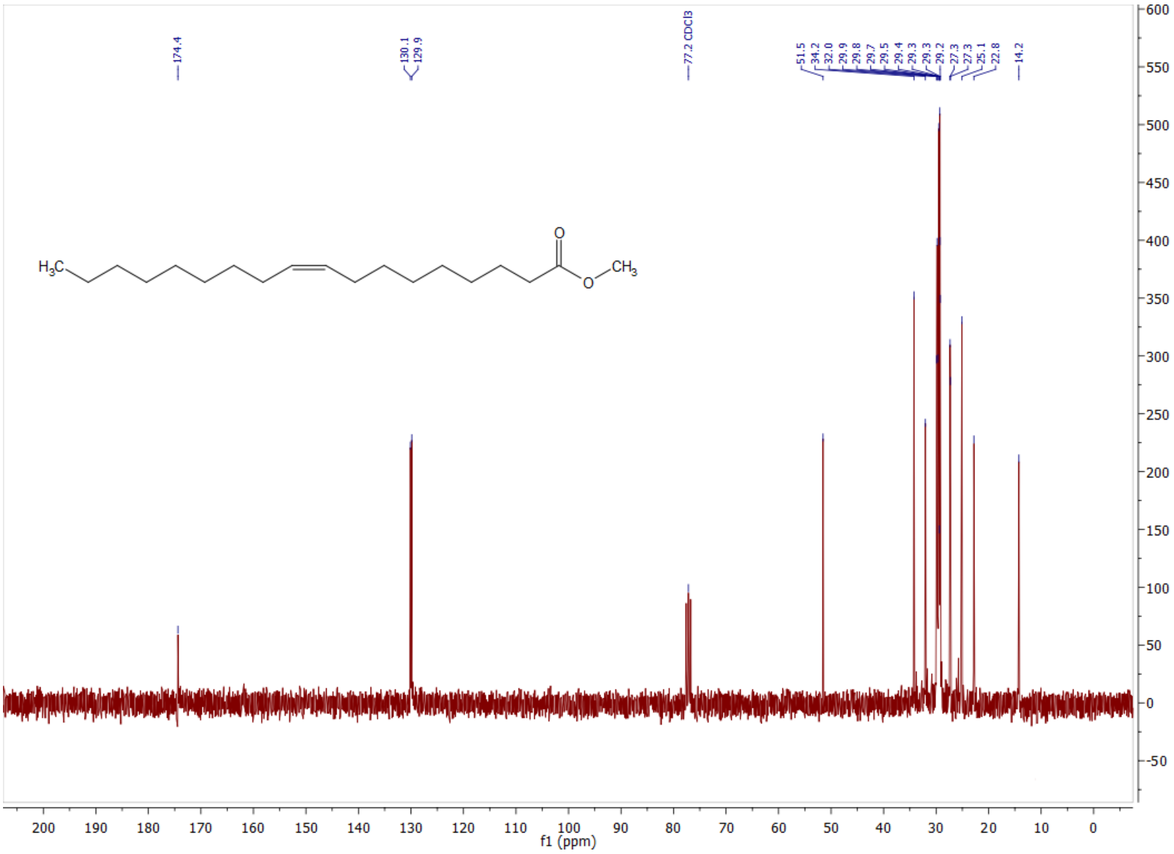

Dimethyl oxalate (~~14a~~15a)

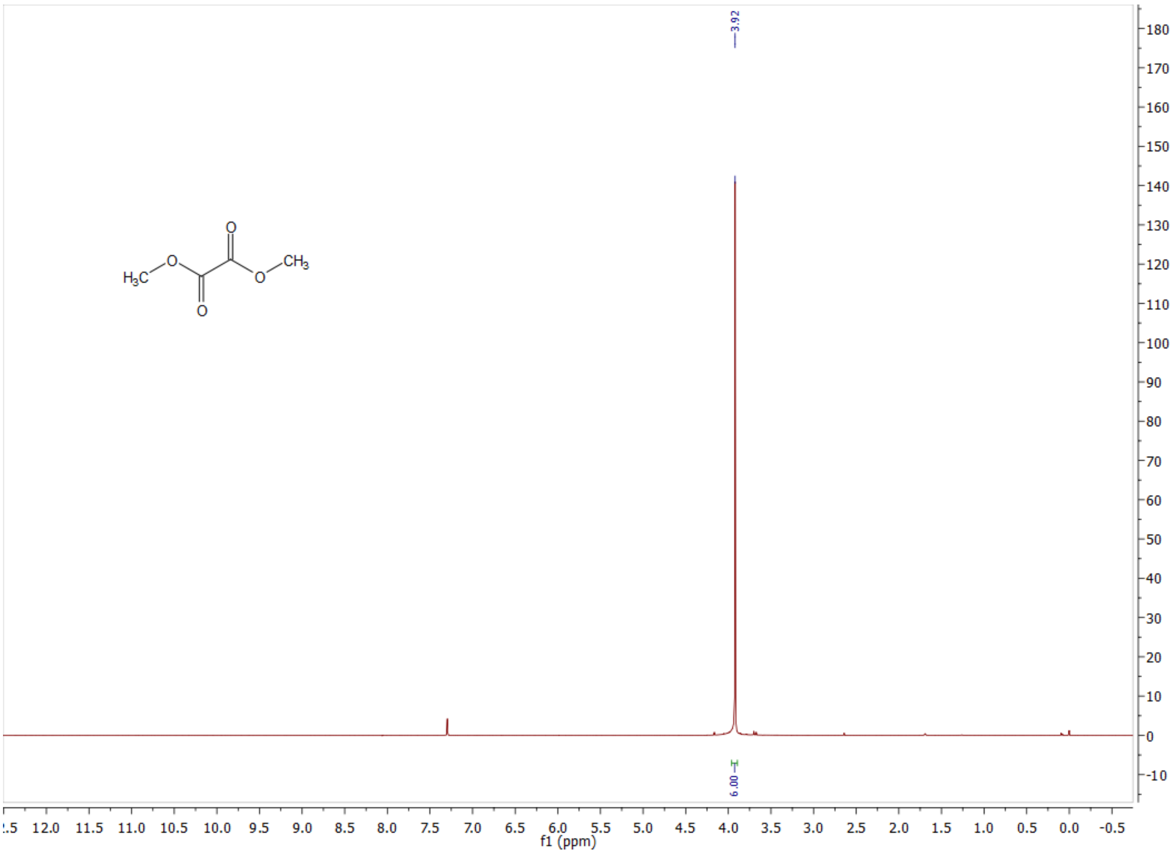

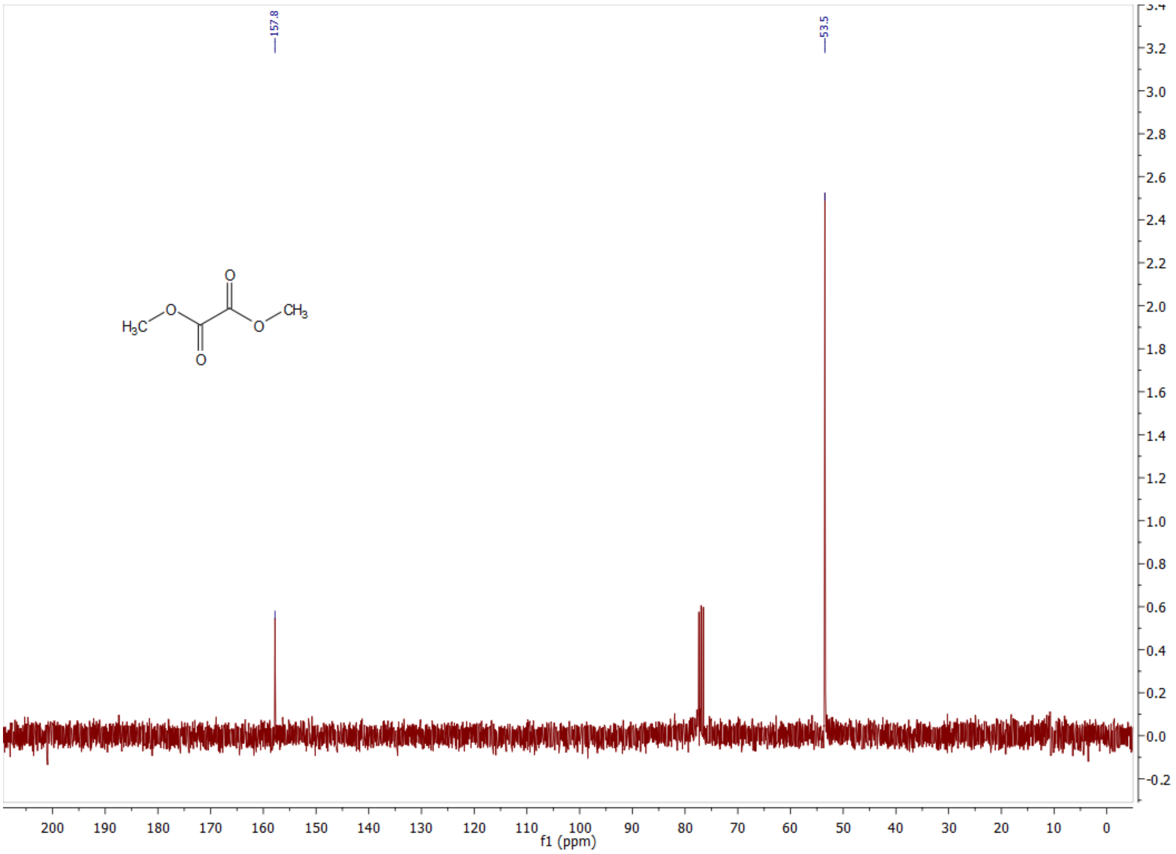

Trimethyl citrate (~~15a~~16a)

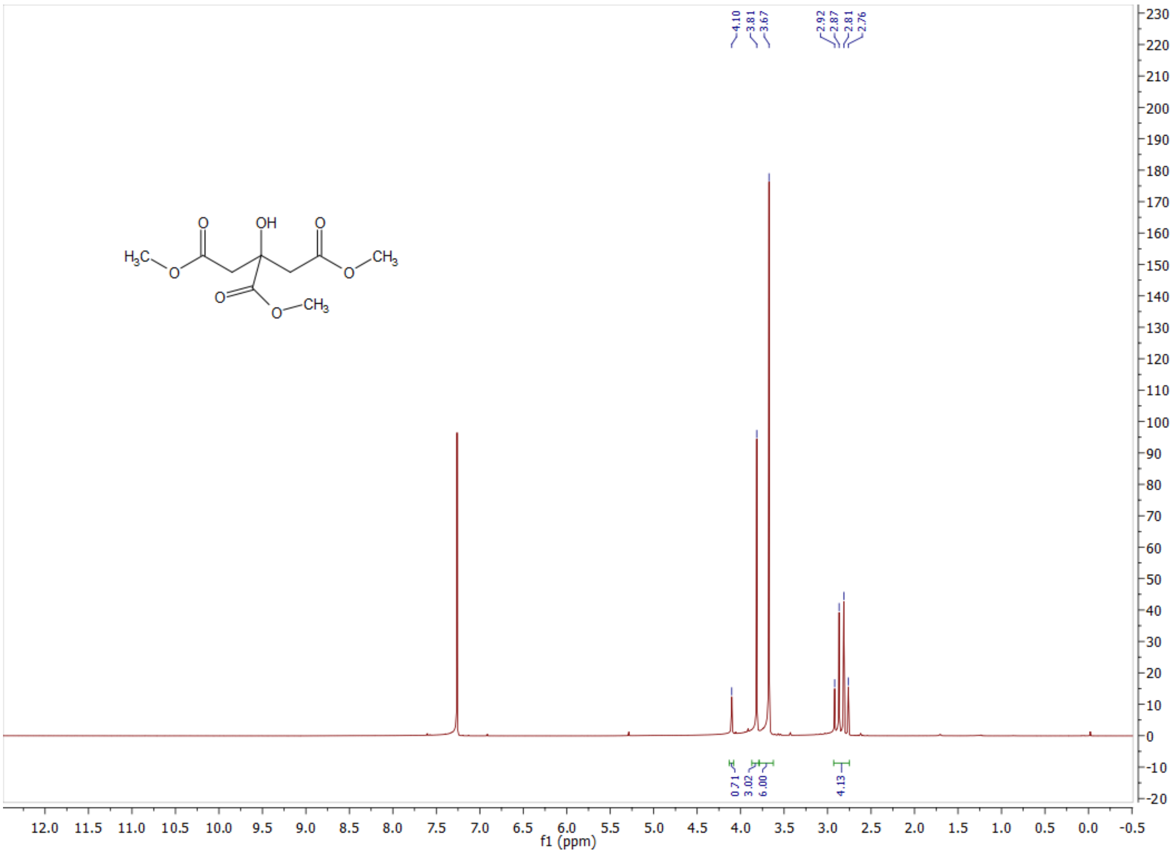

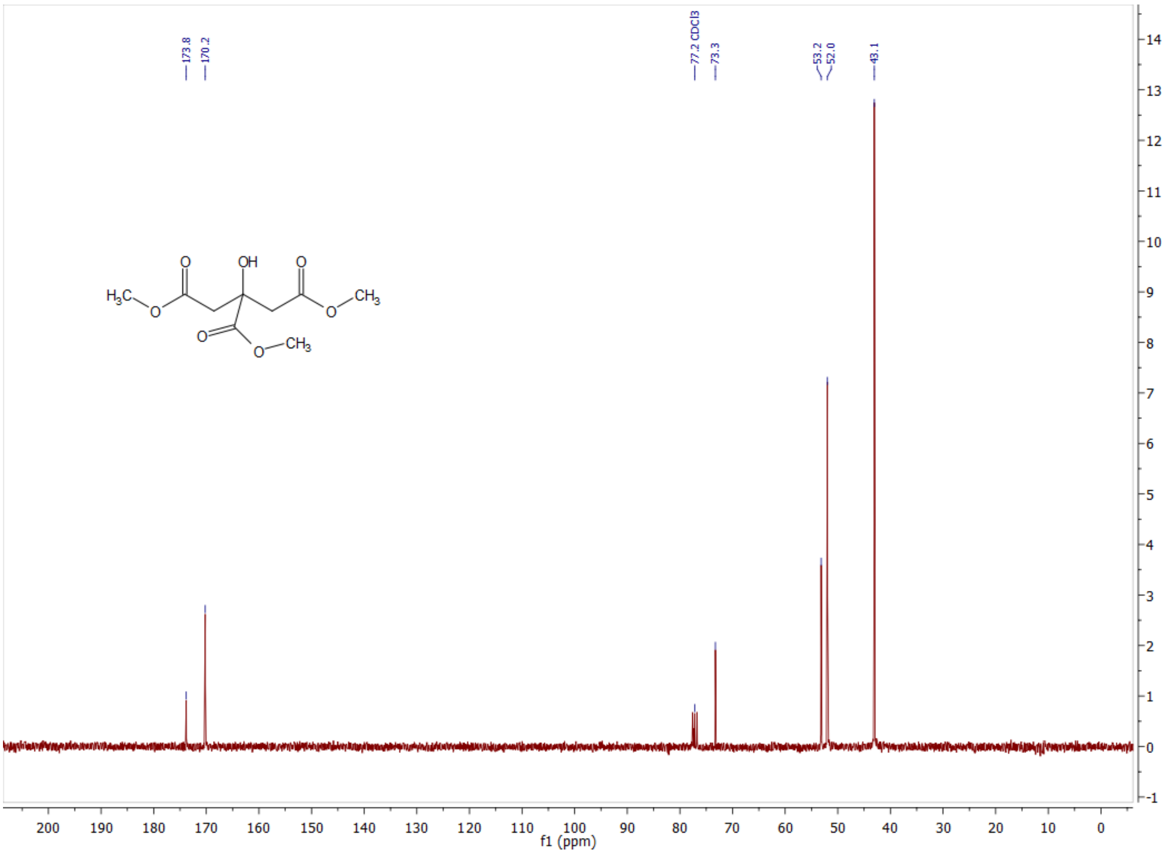

Adamantane-1-carboxylic acid methyl ester (~~16a~~17a)

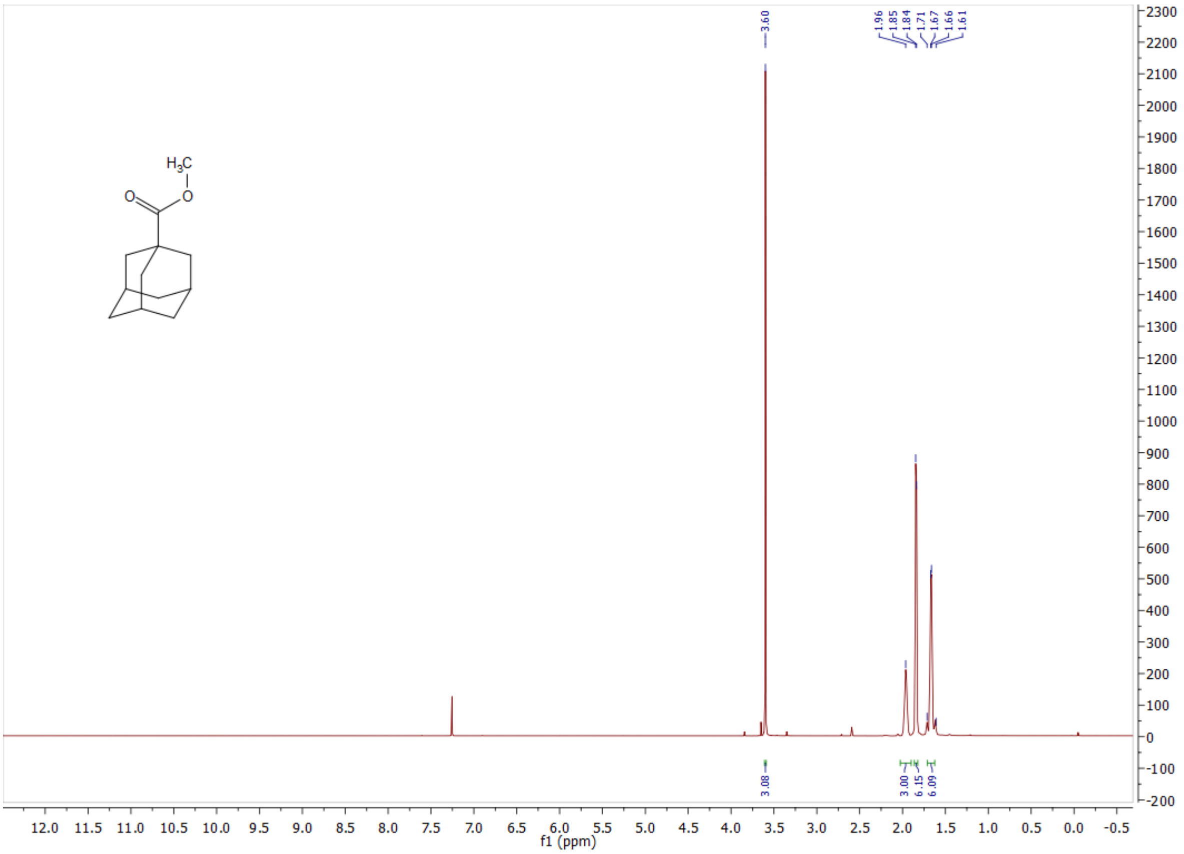

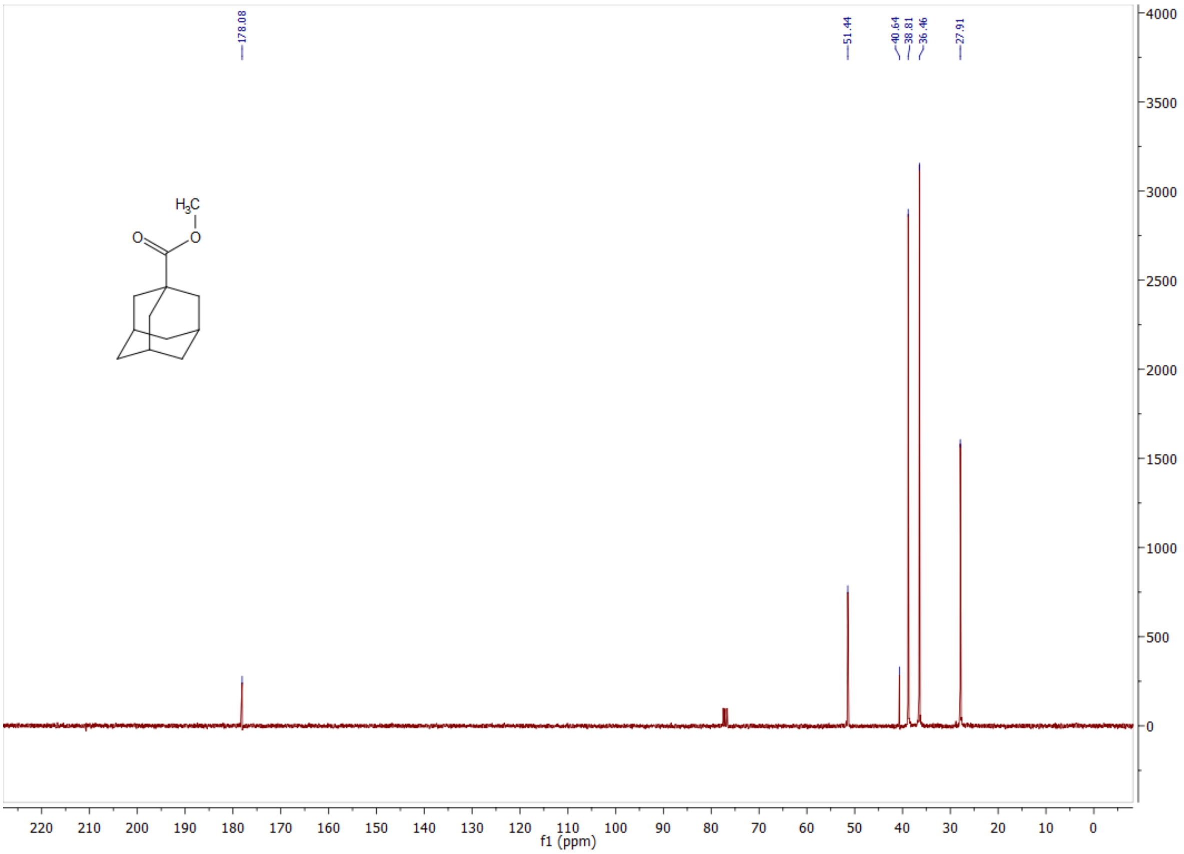

Methyl 3,7,12-trioxo-5*β*-cholan-24-oate (~~17a~~<u>18a</u>)

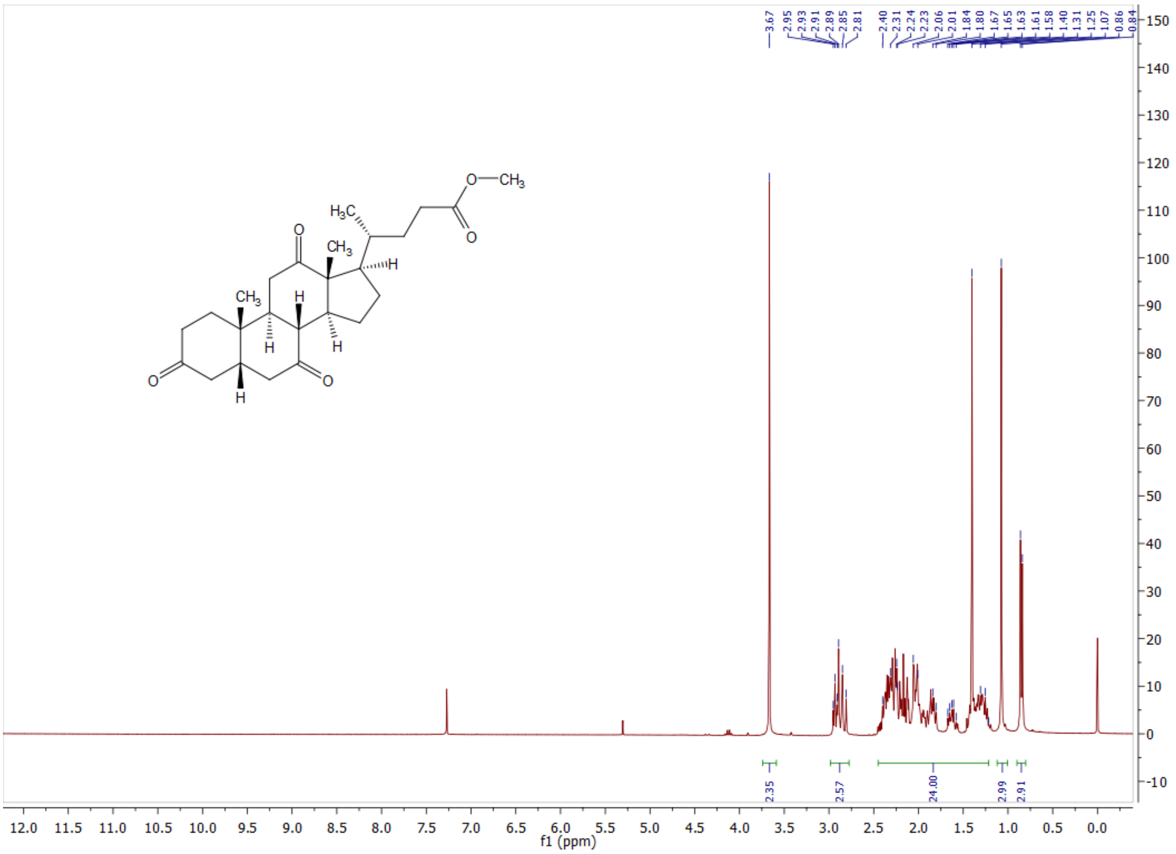

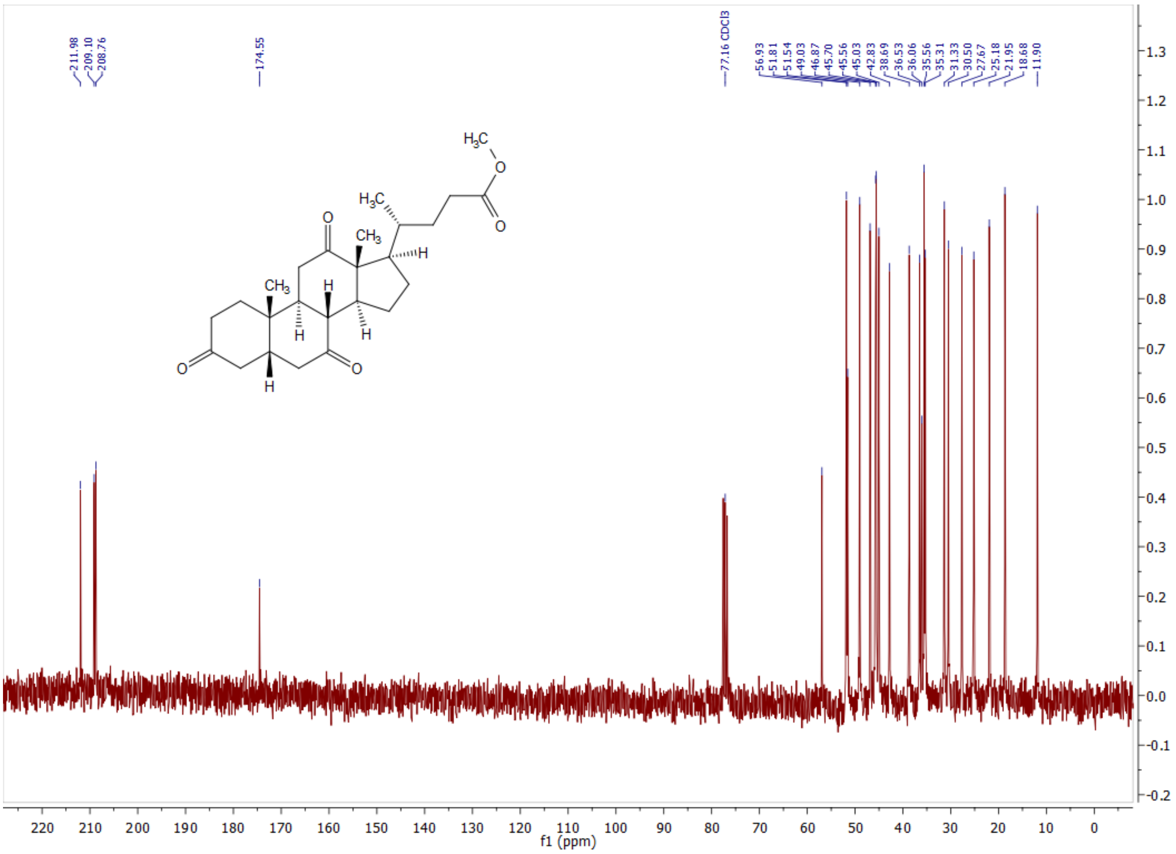

Methyl 3*α*,7*α*,12*α*-trihydroxy-5*β*-cholan-24-oate (~~18a~~19a)

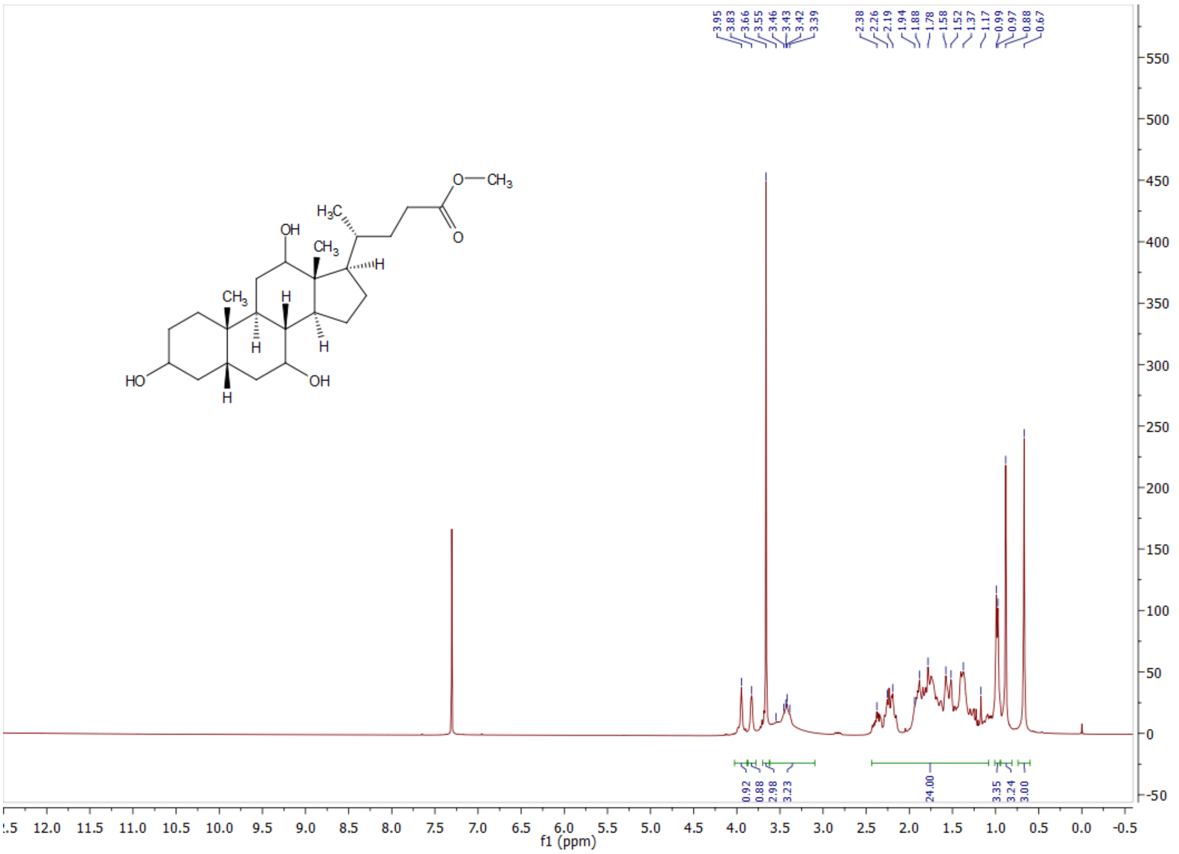

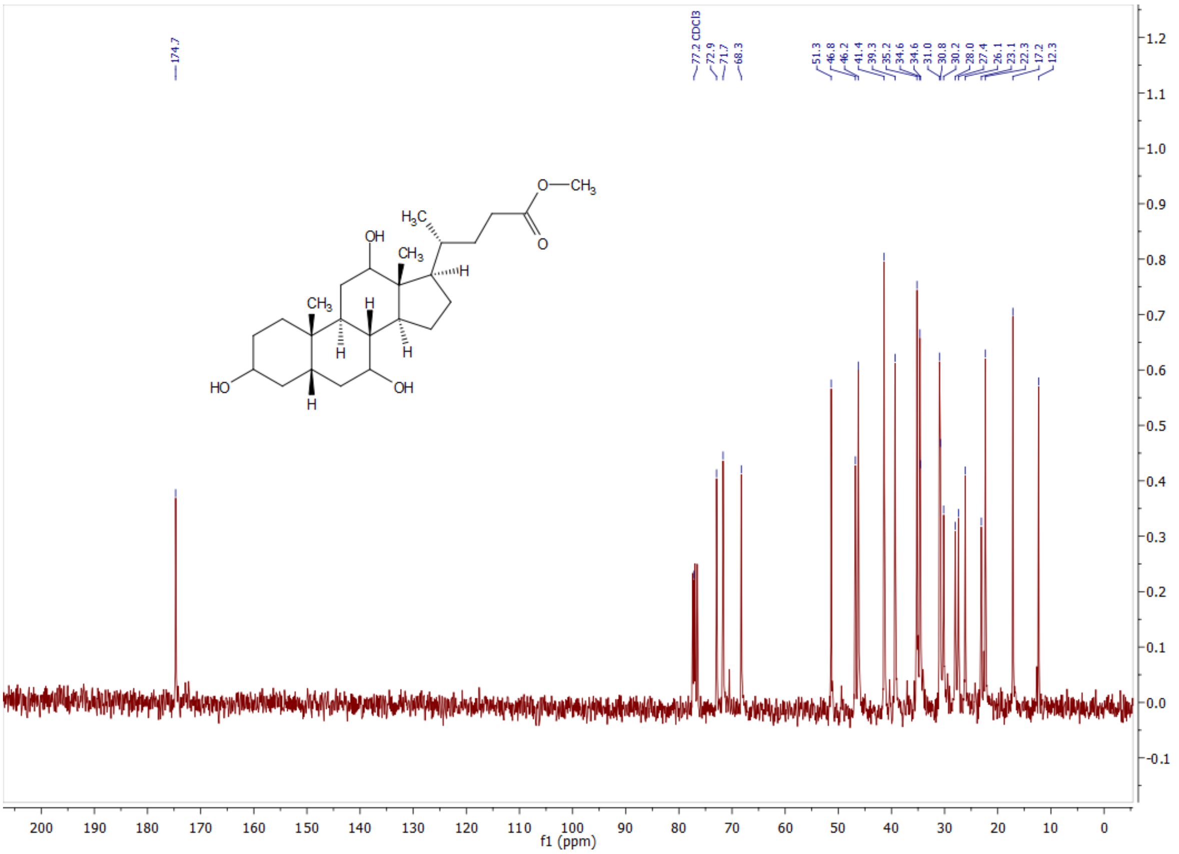

Methyl 2-cyanoacetate (20a)

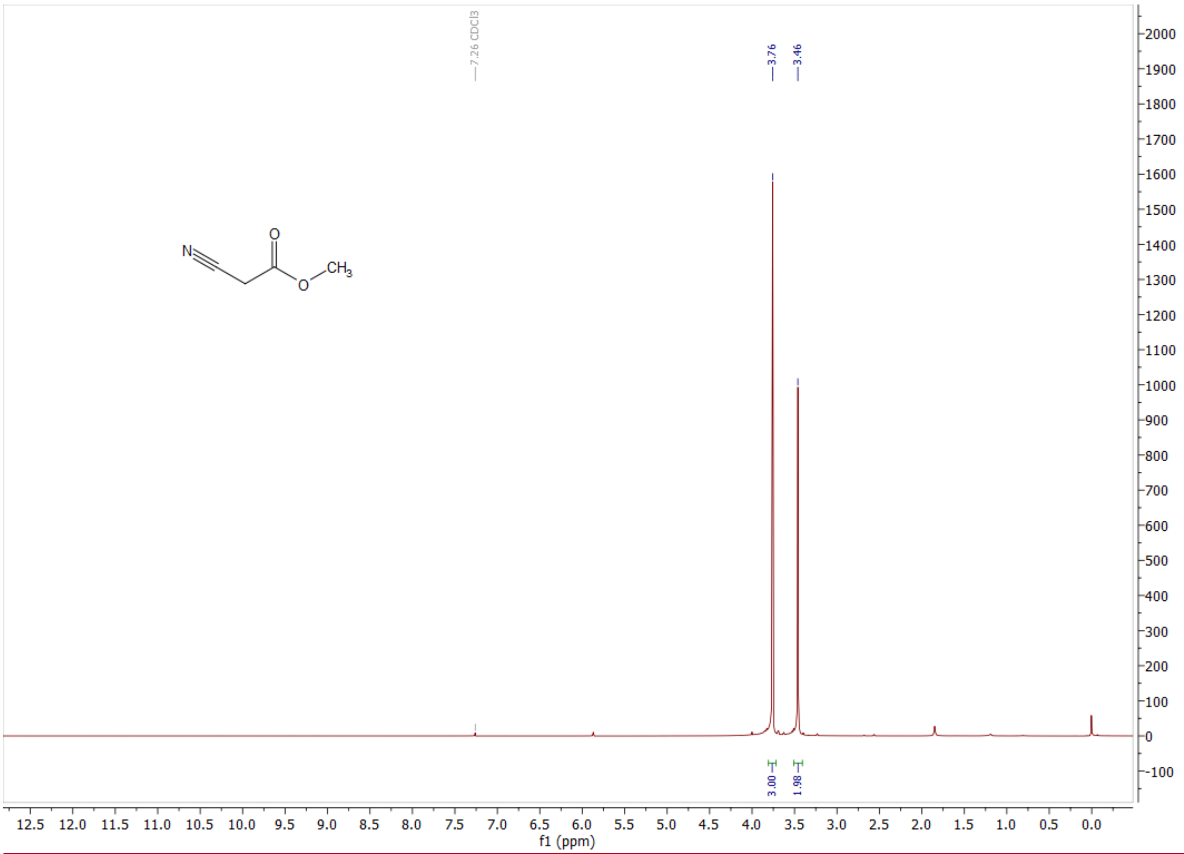

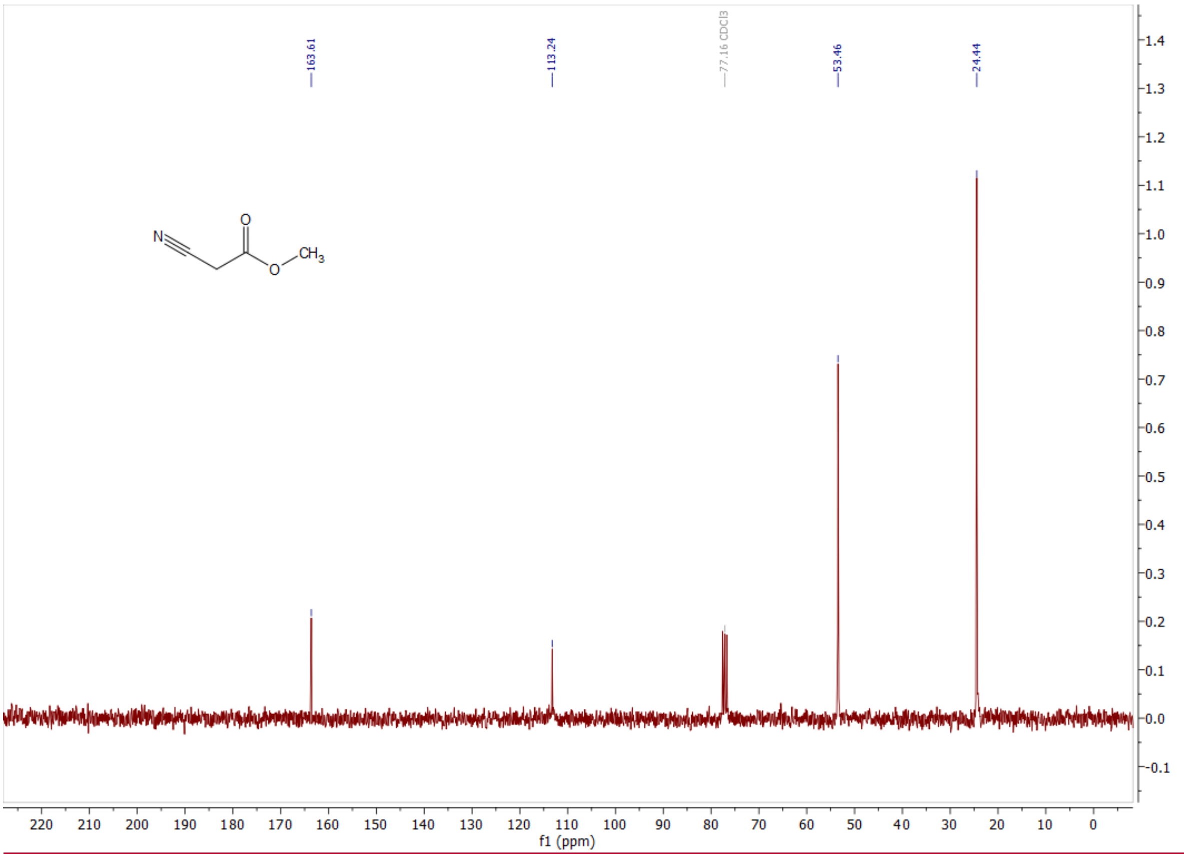

(*S*)-Methyl 2-acetamido-3-phenylpropanoate (21a)

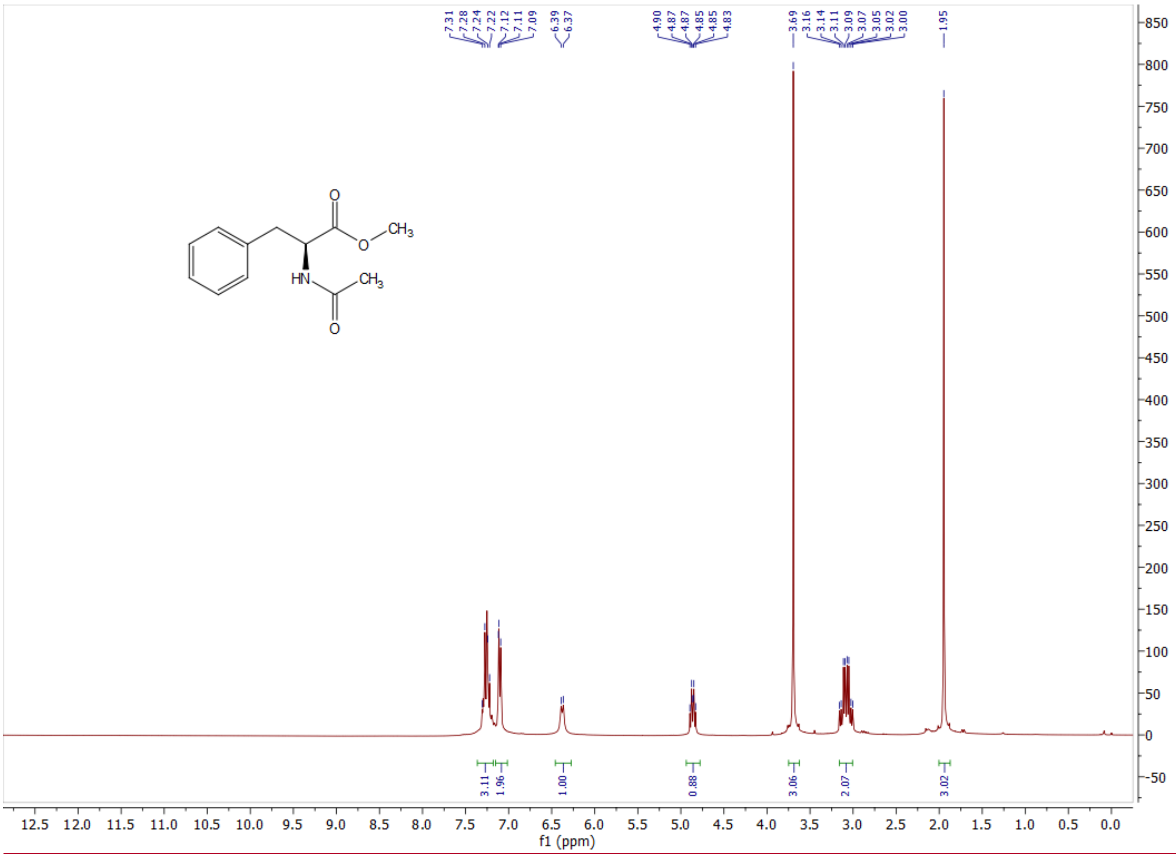

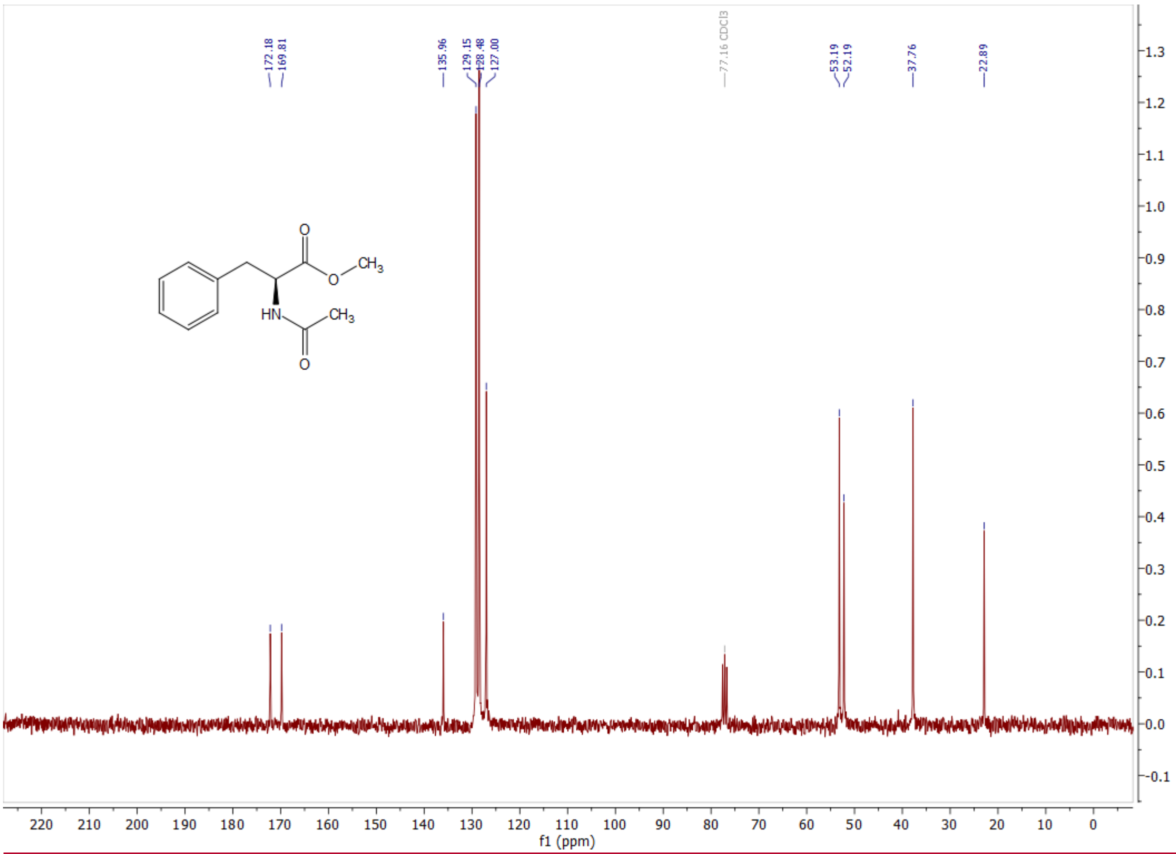

Methyl 2-(1*H*-indol-3-yl)acetate (22a)

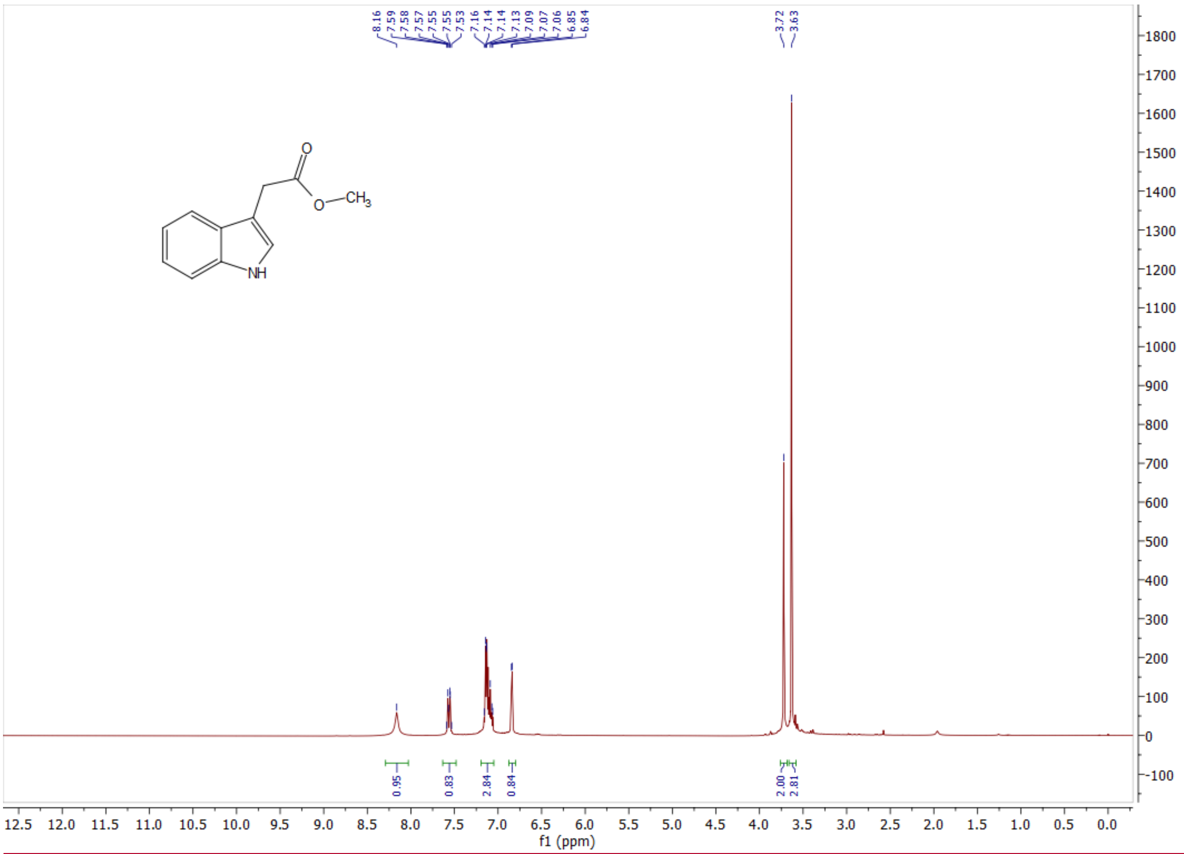

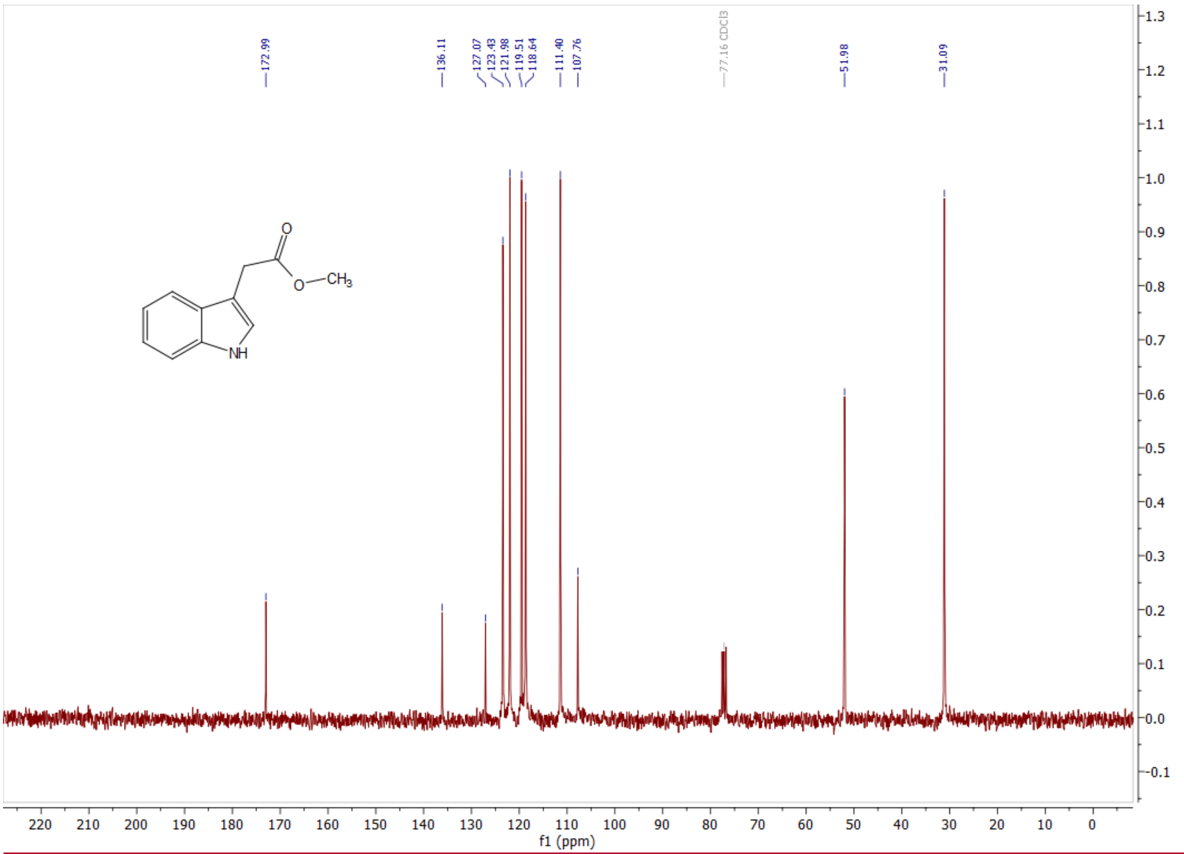

Methyl 4-oxo-4-phenylbutanoate (23a)

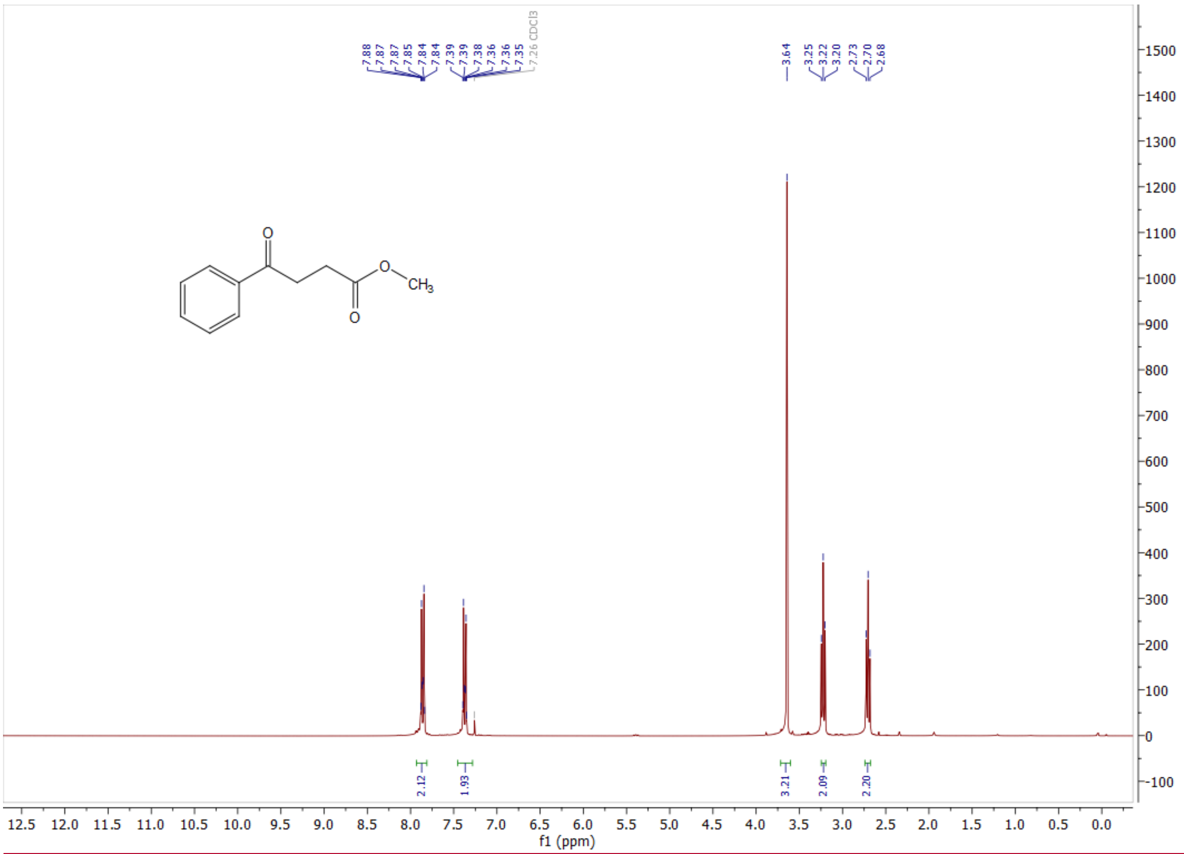

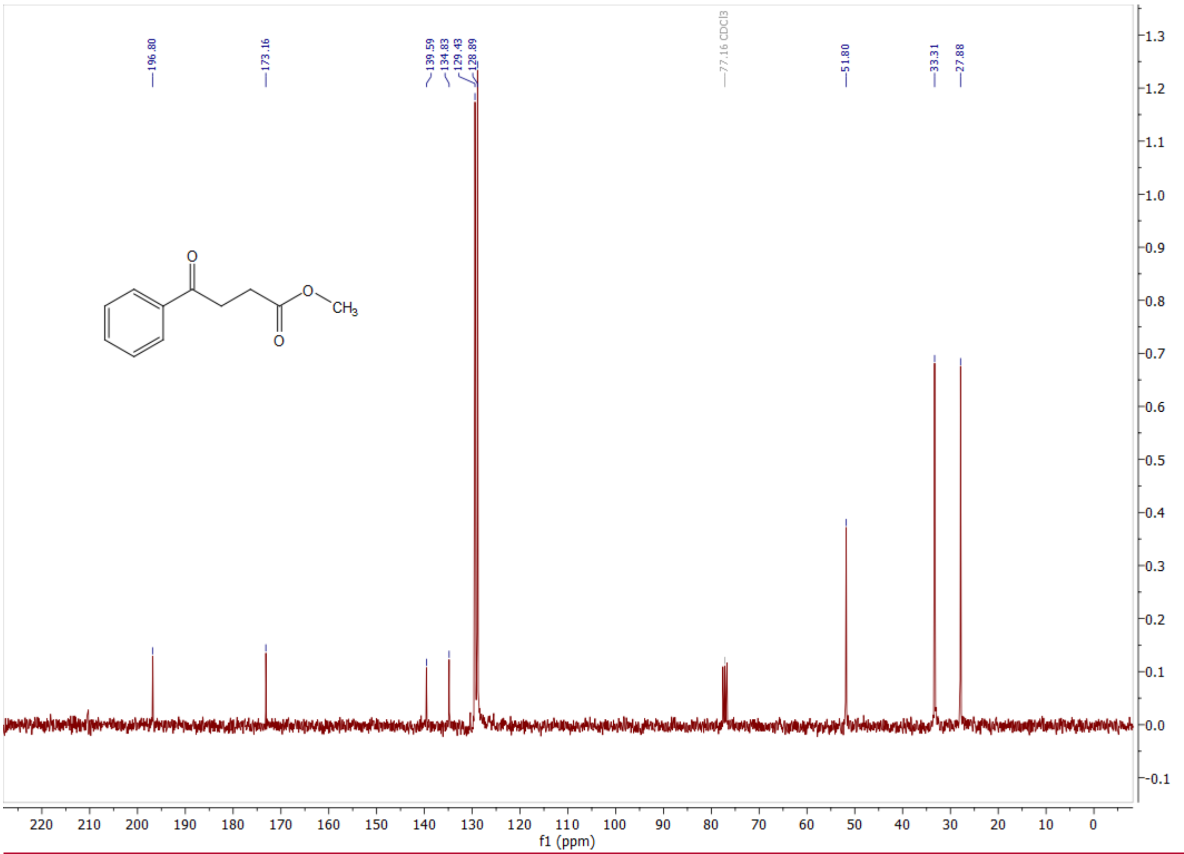

2-Fluoroethyl benzoate (1b)

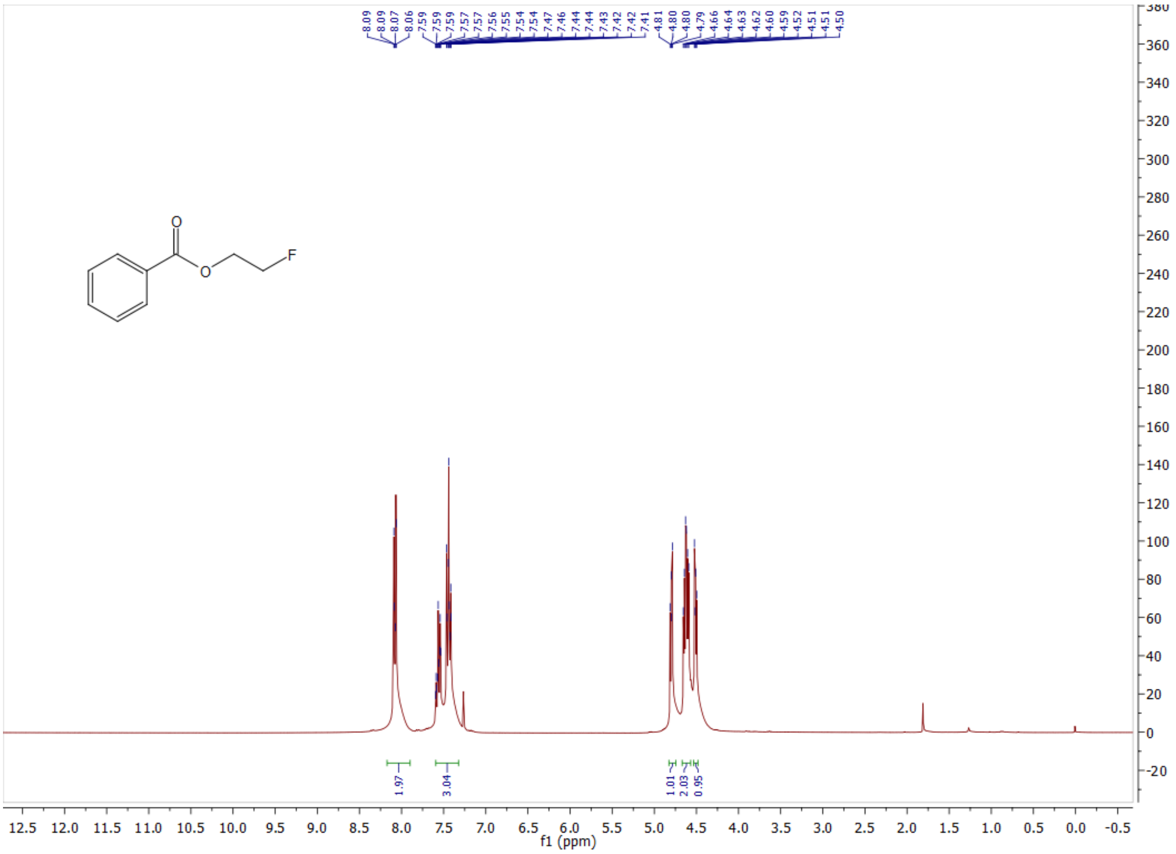

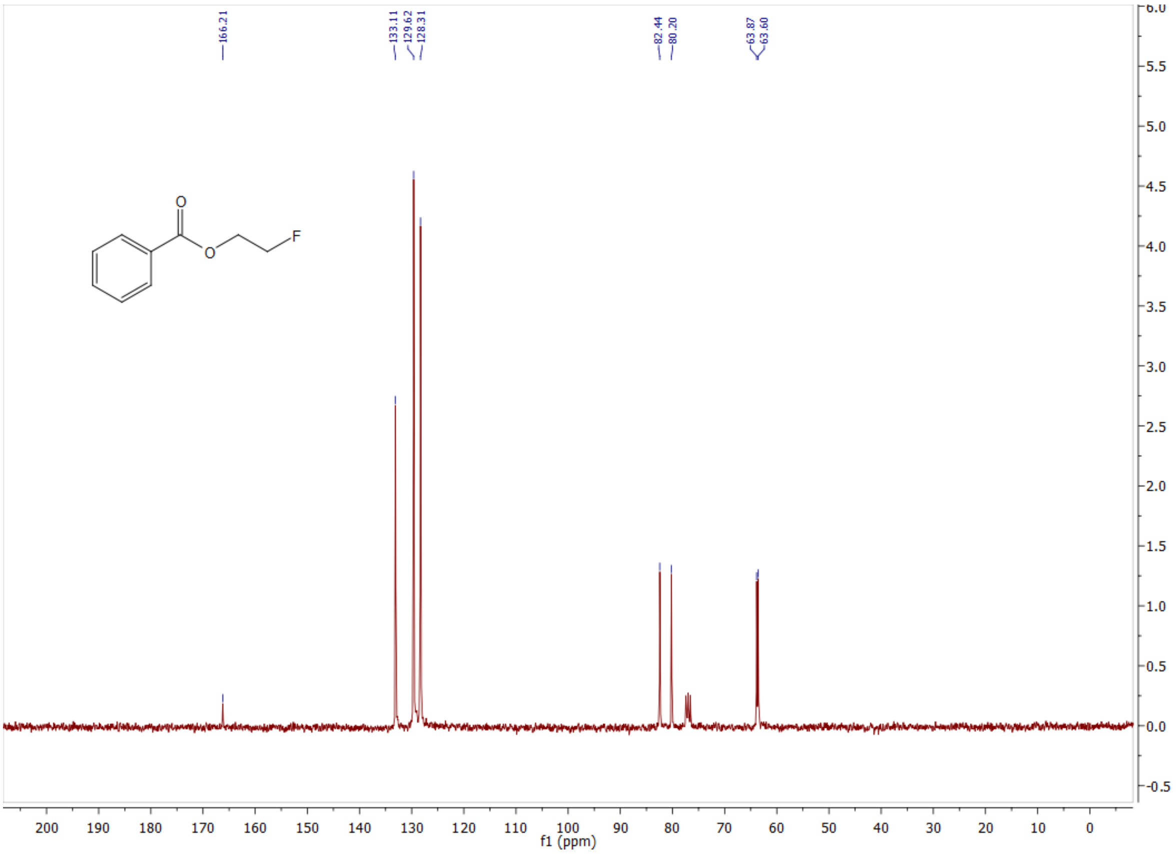

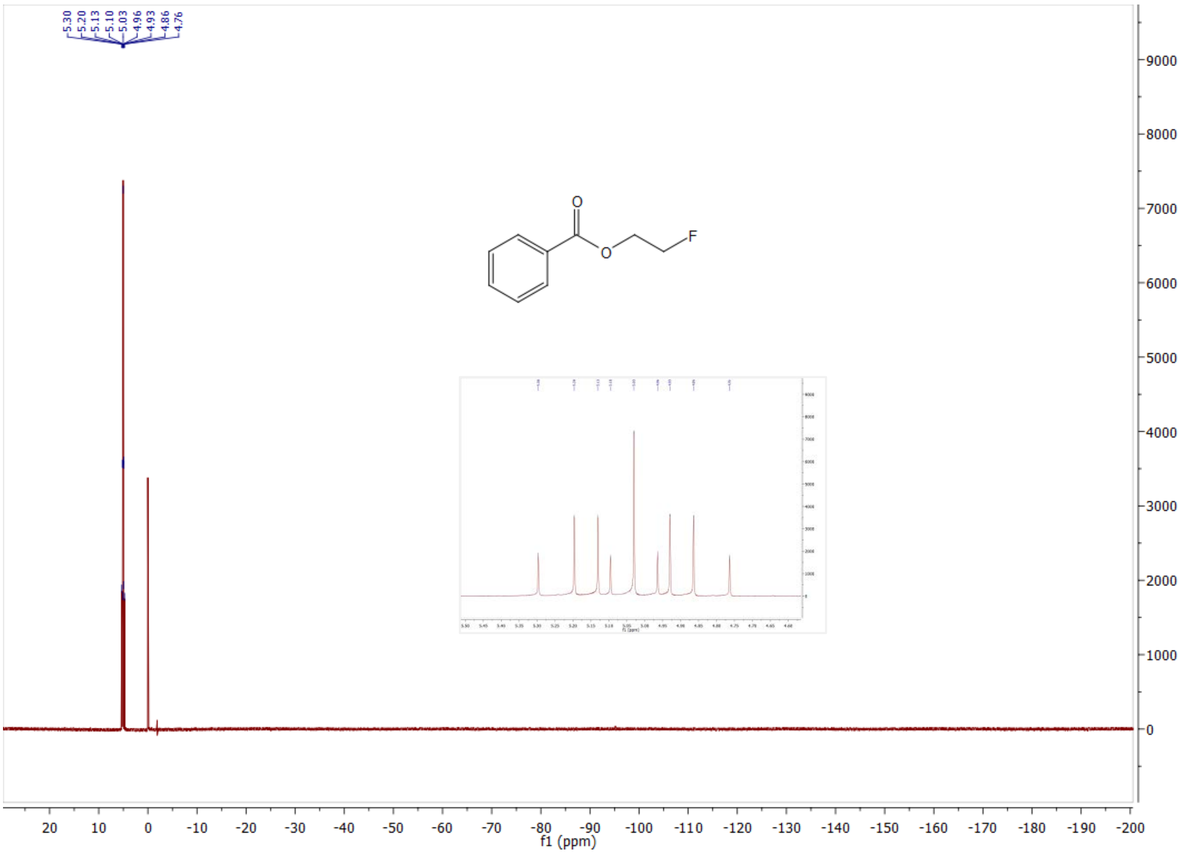
5.30
5.20
5.13
5.10
5.03
4.96
4.93
4.86
4.76
O
O
F
9000
8000
7000
6000
5000
4000
3000
2000
1000
0
20
10
0
-10
-20
-30
-40
-50
-60
-70
-80
-90
-100
-110
-120
-130
-140
-150
-160
-170
-180
-190
-200
f1 (ppm)

Isopropyl benzoate (1c)

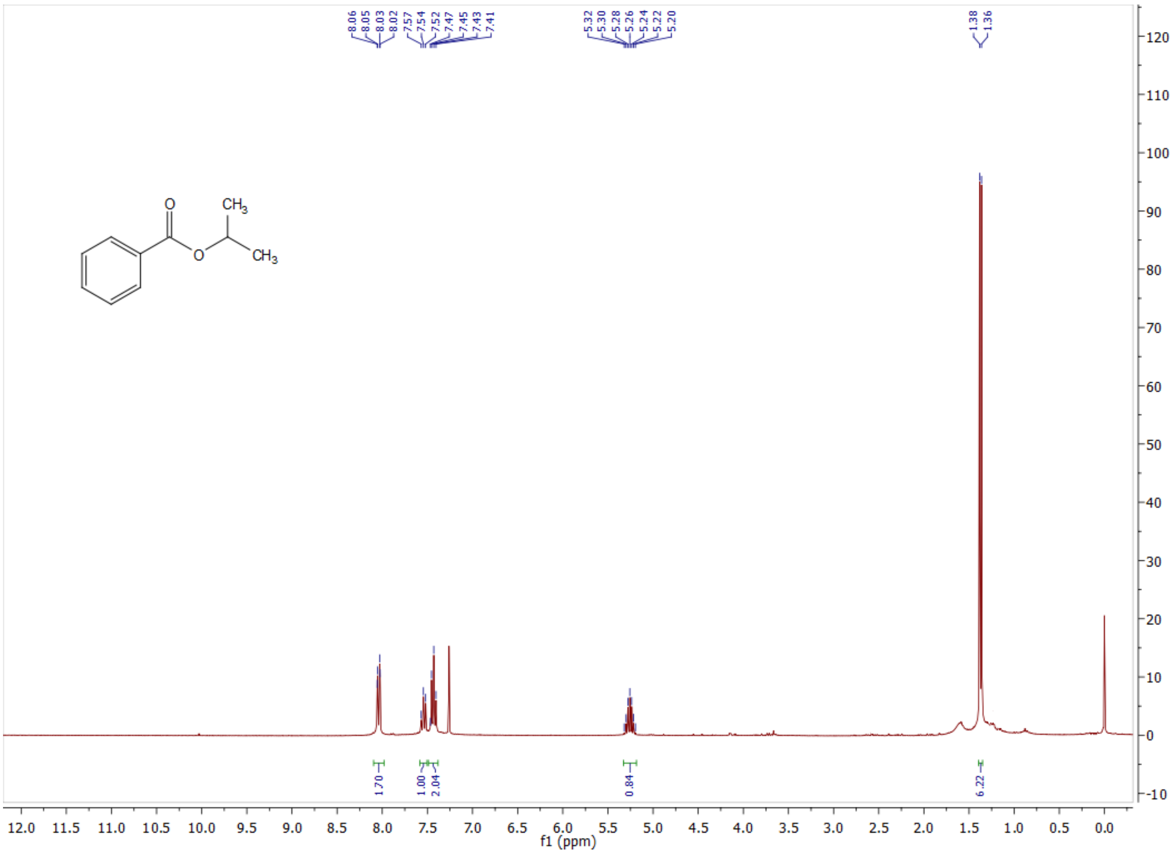

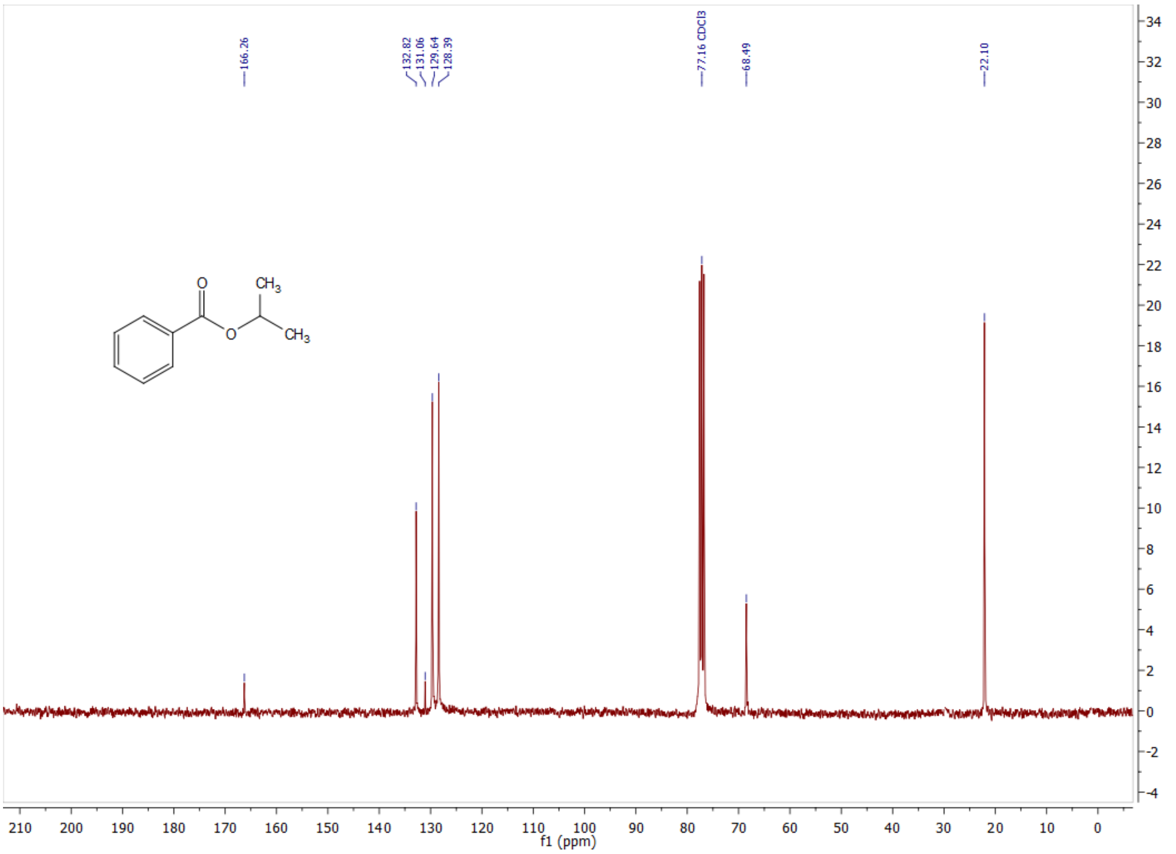

2-Fluoroethyl octanoate (2b)

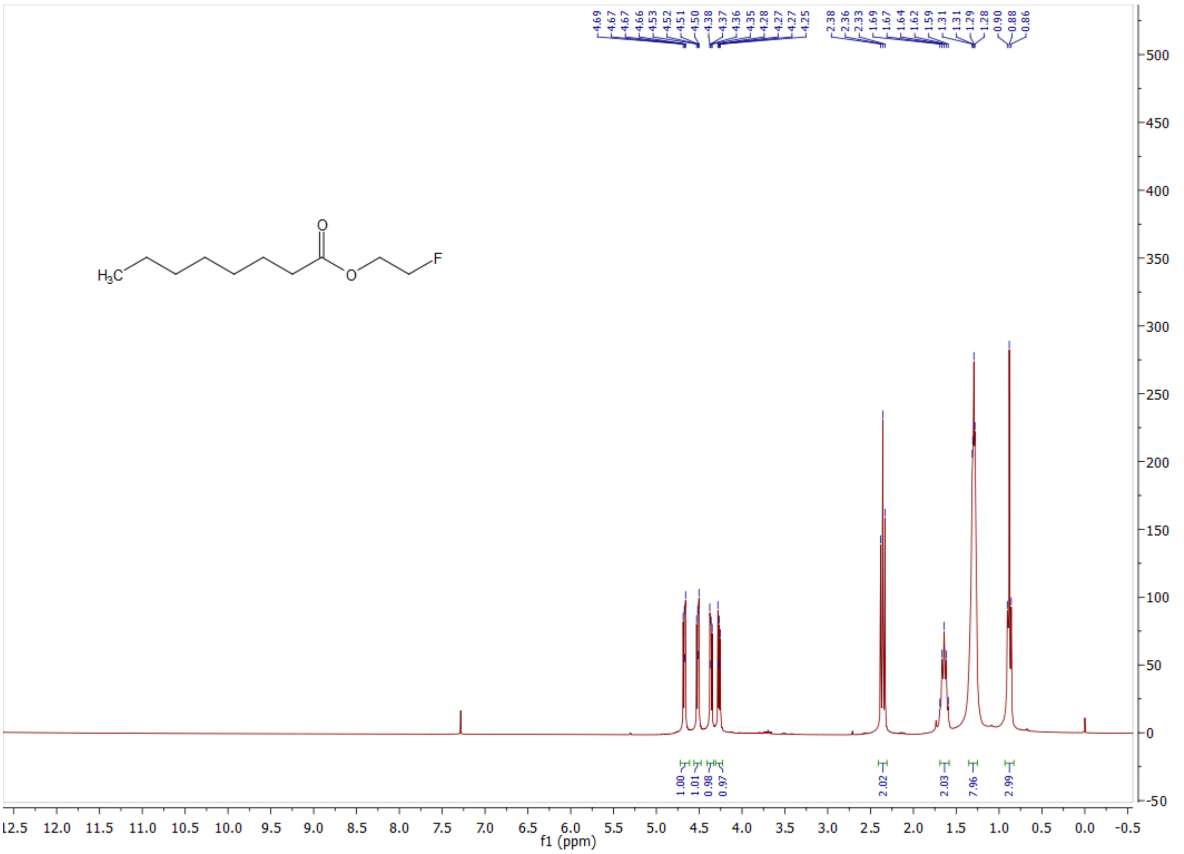

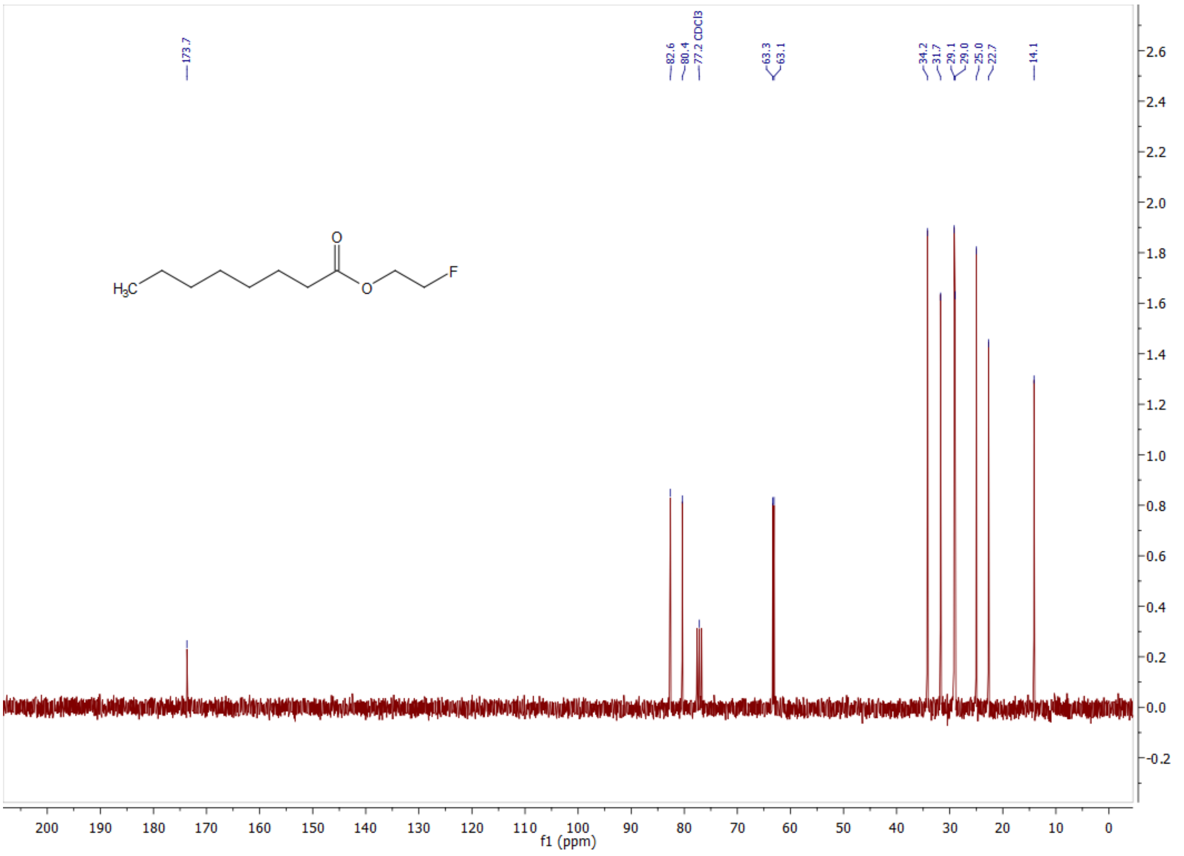

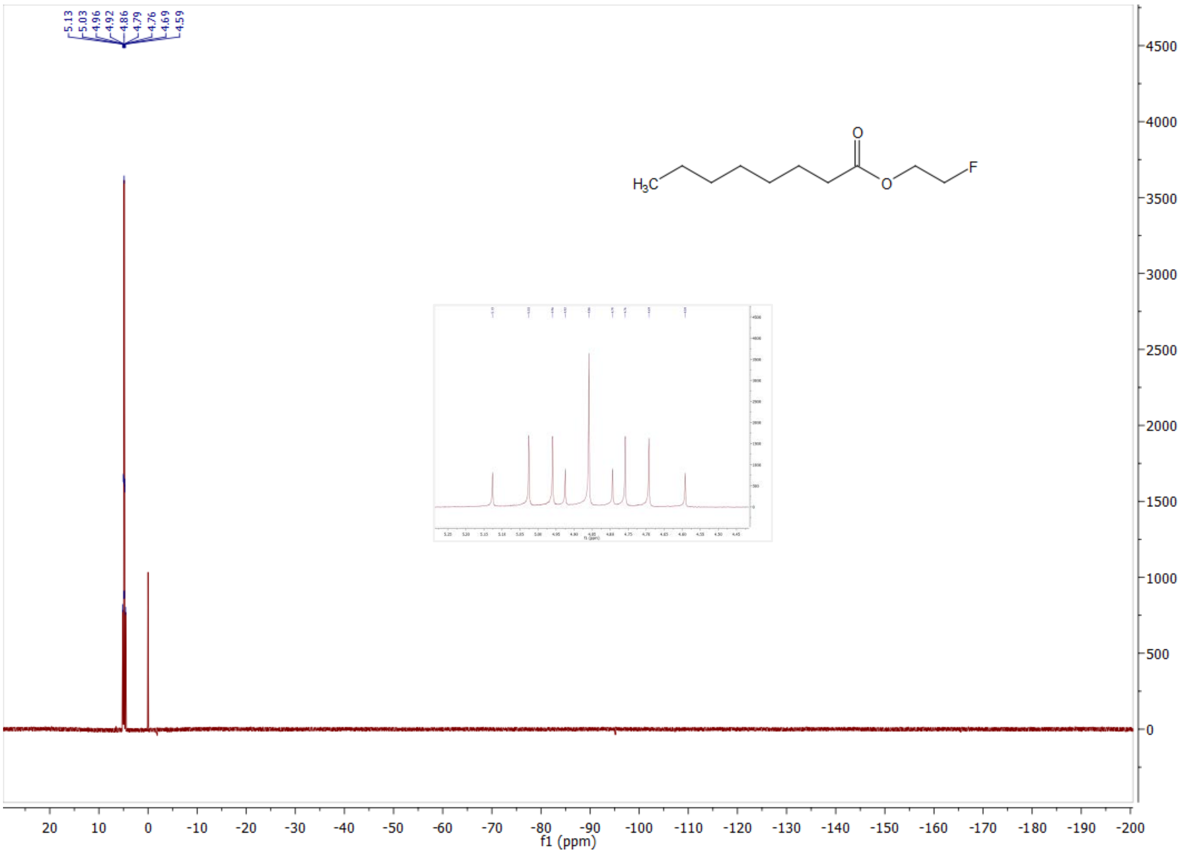

5.13
5.03
4.96
4.92
4.86
4.79
4.76
4.69
4.59
O
H3C
O
F
4500
4000
3500
3000
2500
2000
1500
1000
500
0
20 10 0 -10 -20 -30 -40 -50 -60 -70 -80 -90 -100 -110 -120 -130 -140 -150 -160 -170 -180 -190 -200
f1 (ppm)

Isopropyl octanoate (2c)

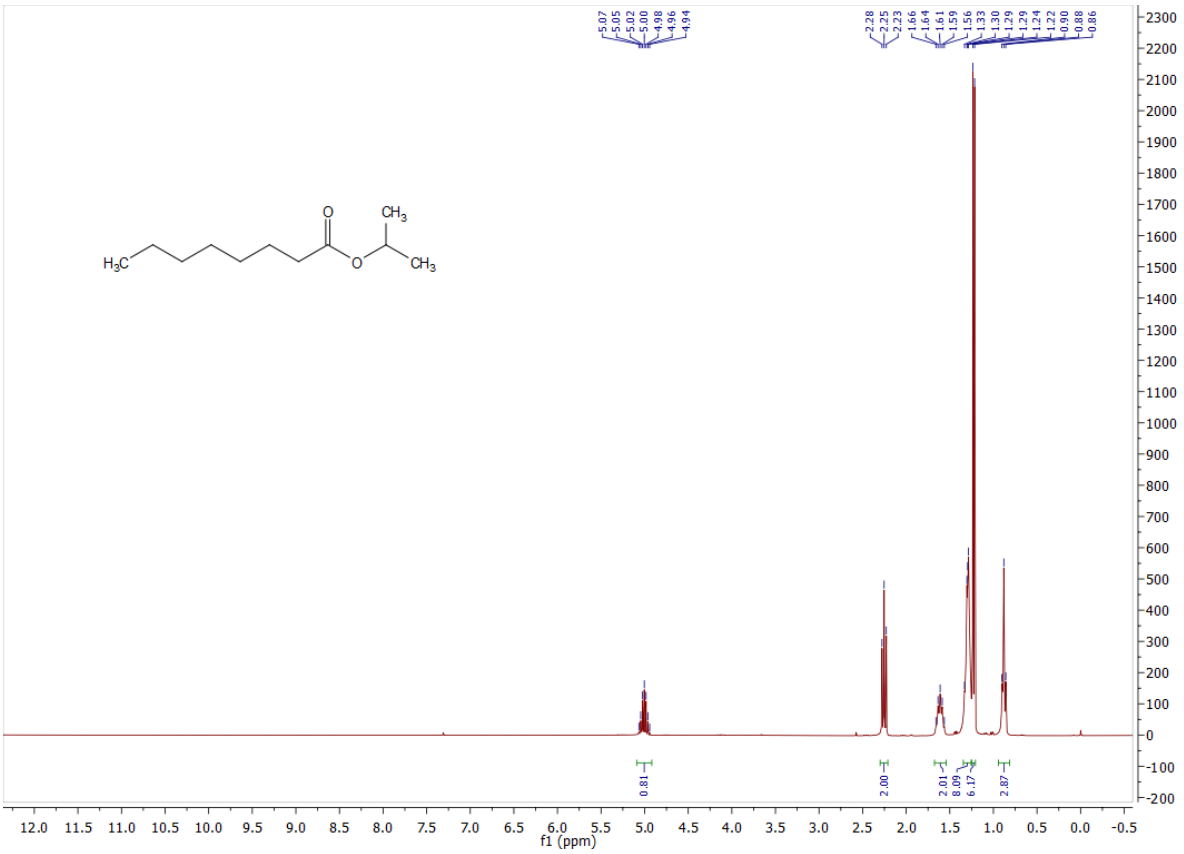

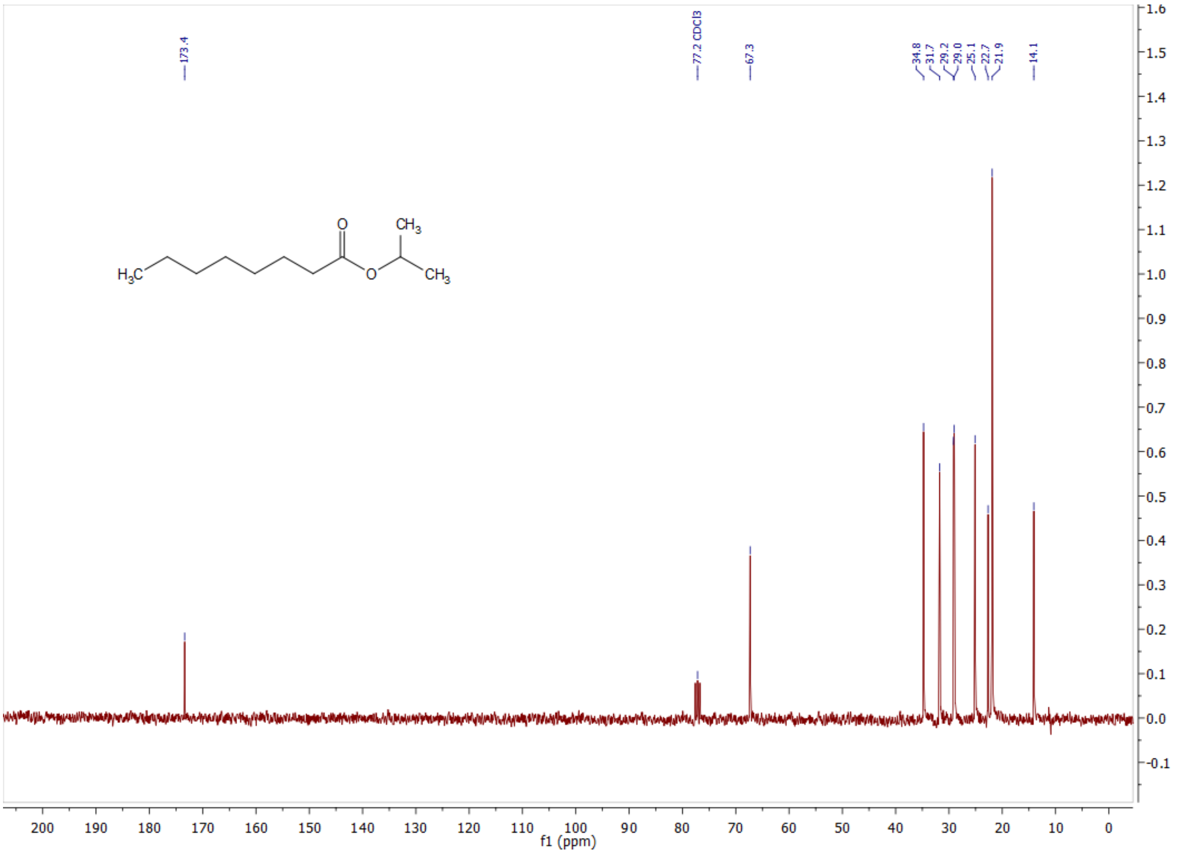

***n*-Butyl benzoate (1d)**

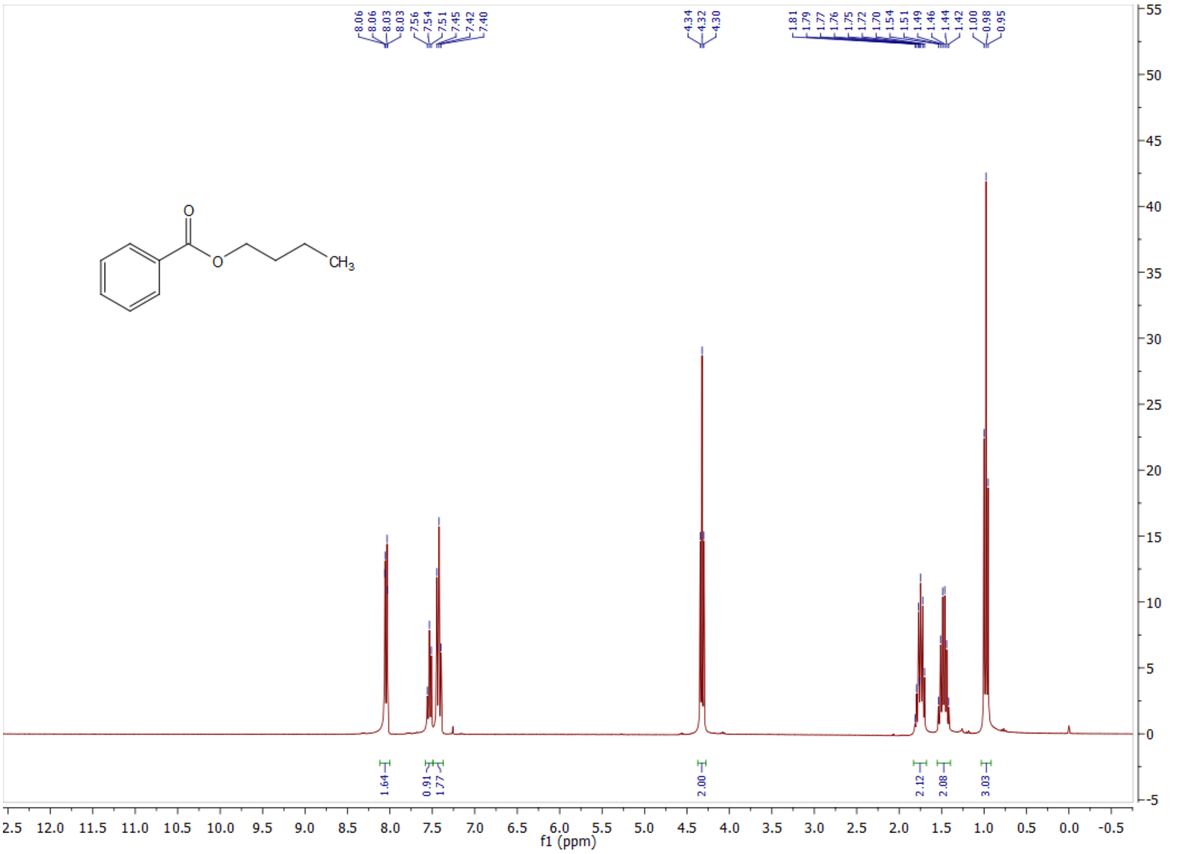

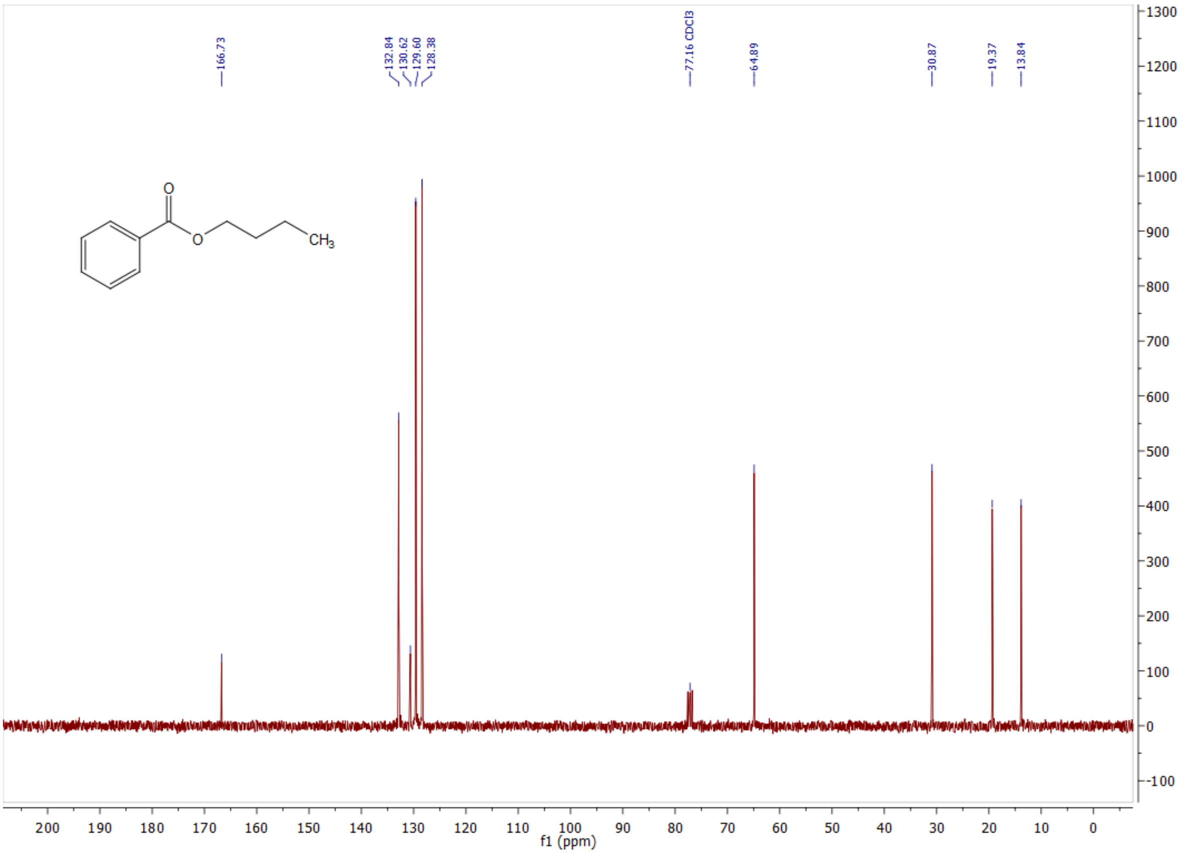

n-Butyl octanoate (2d)

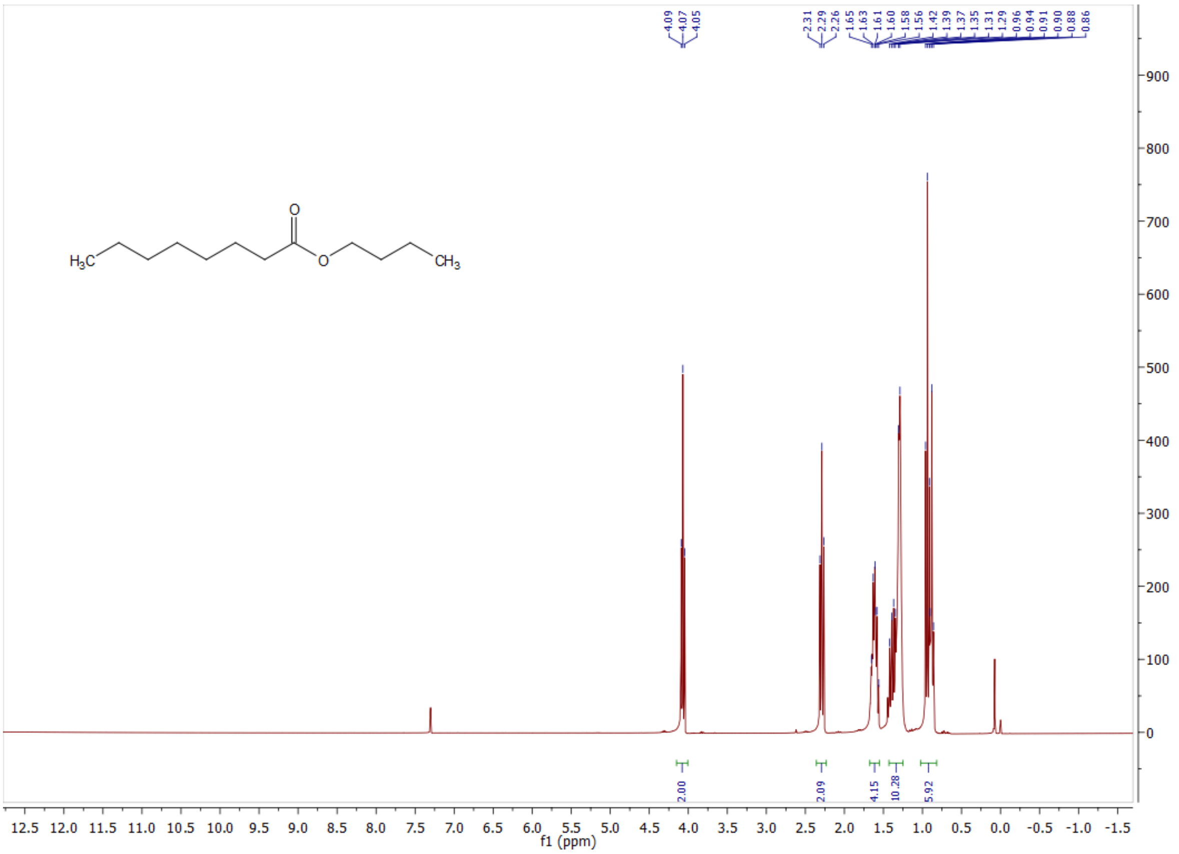

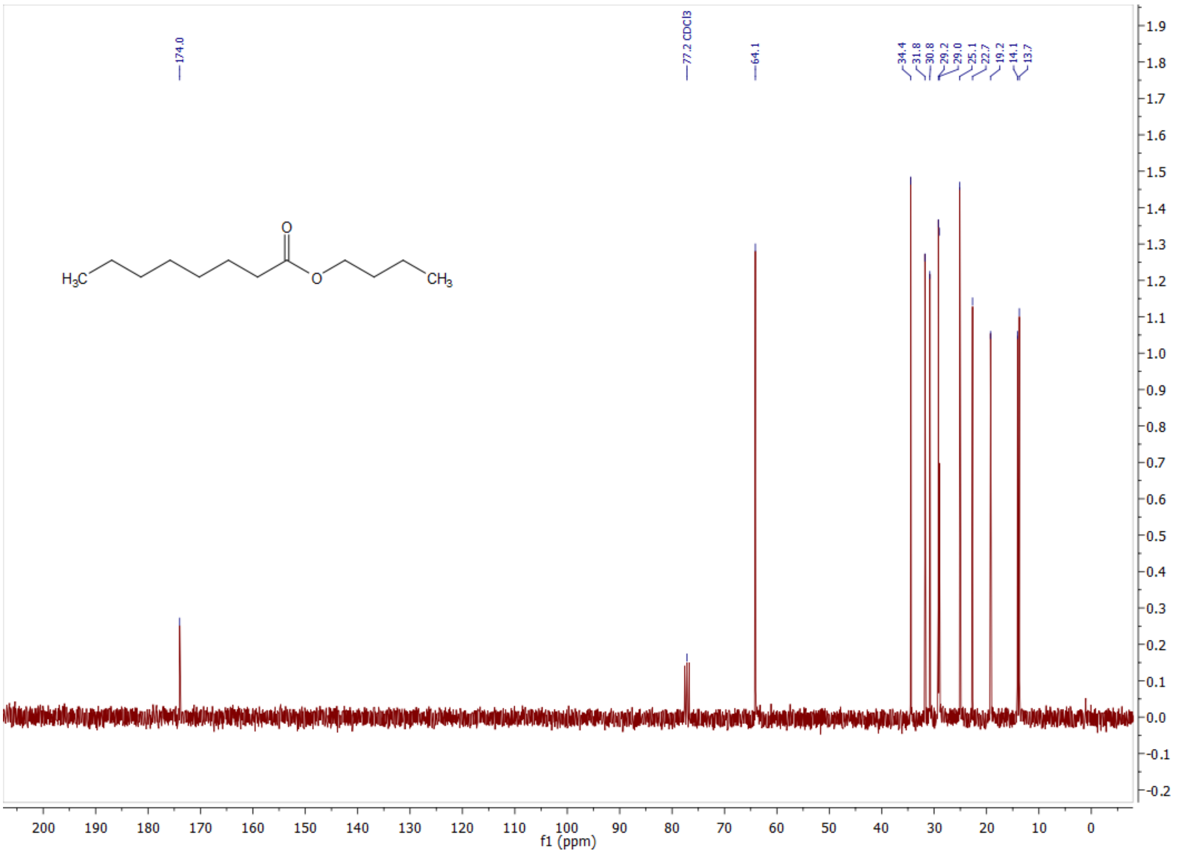

***n*-Octyl benzoate (1f)**

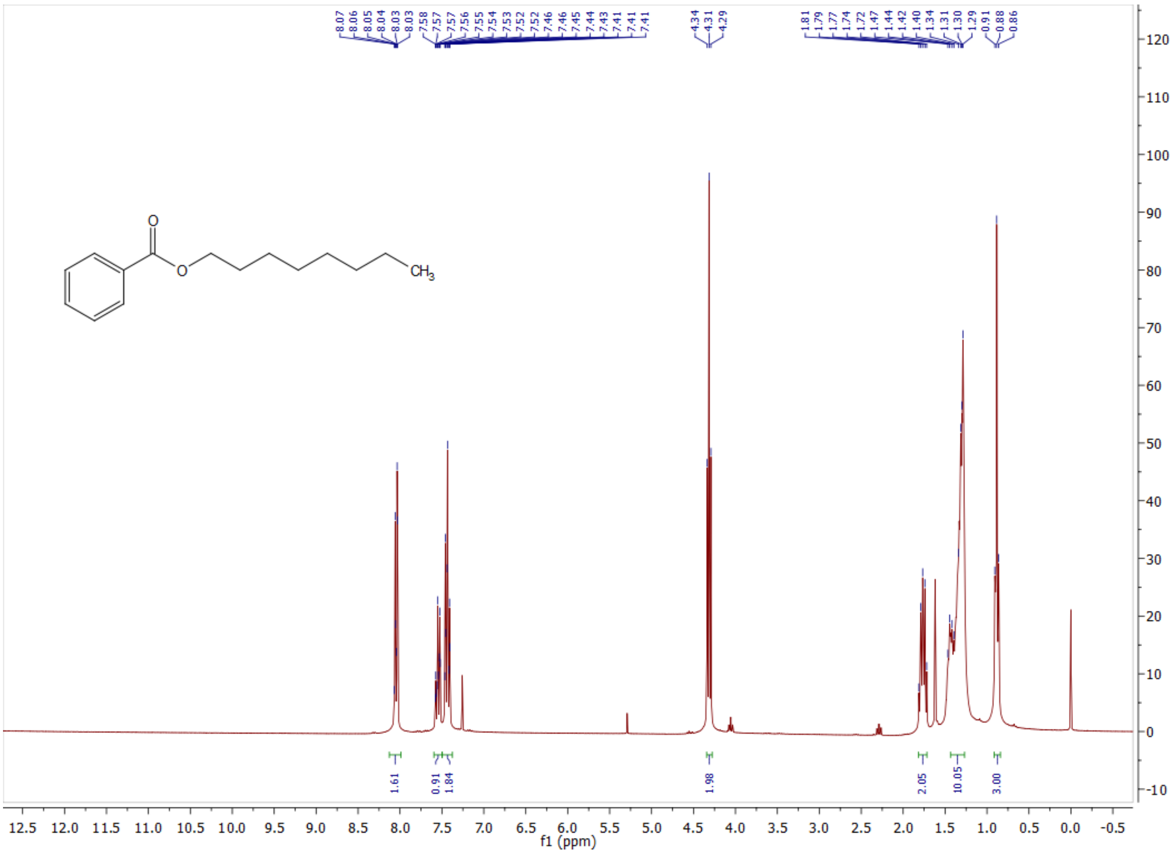

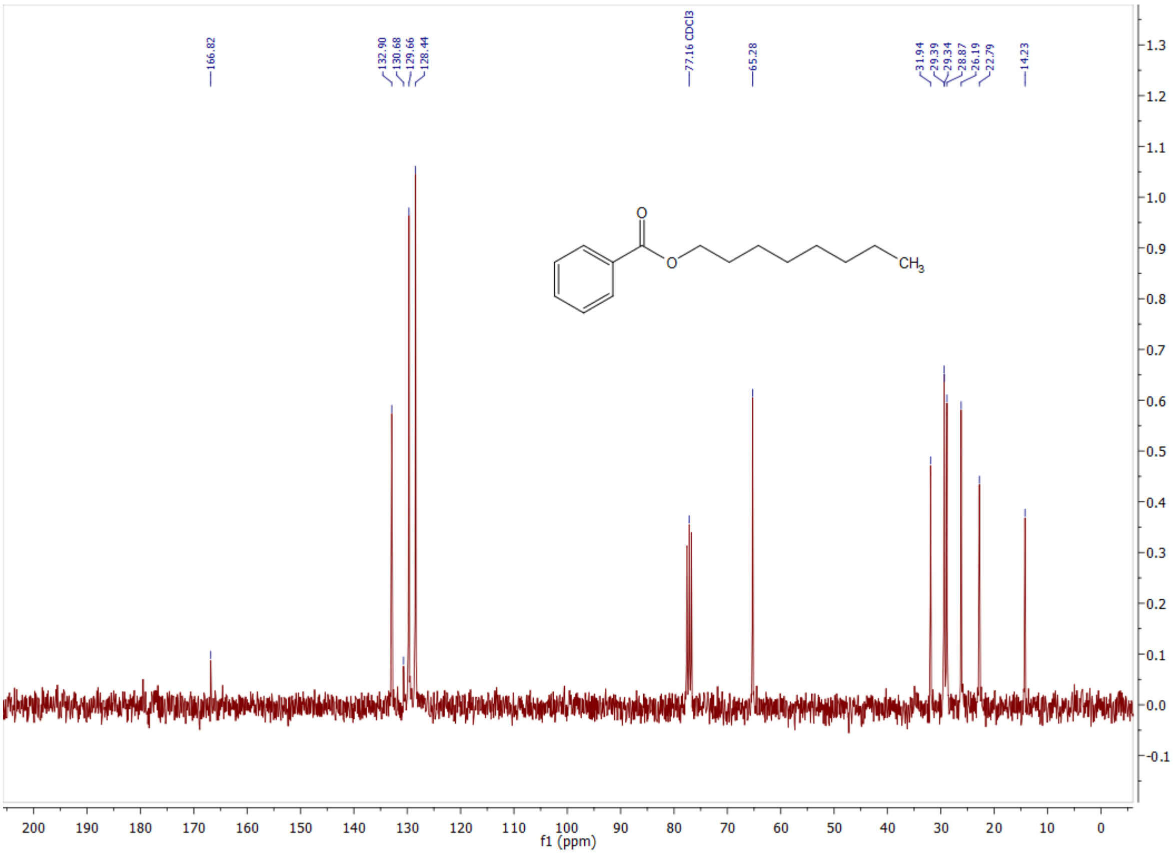

n-Octyl octanoate (2f)

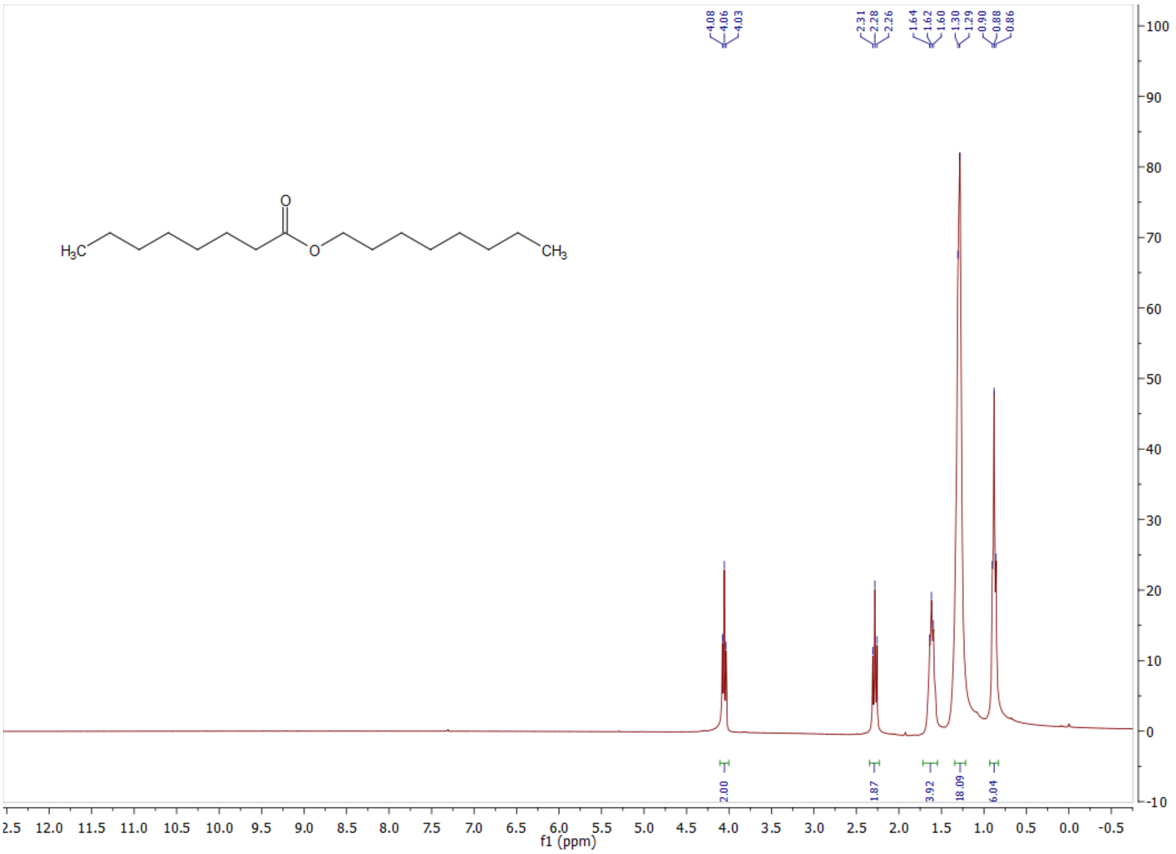

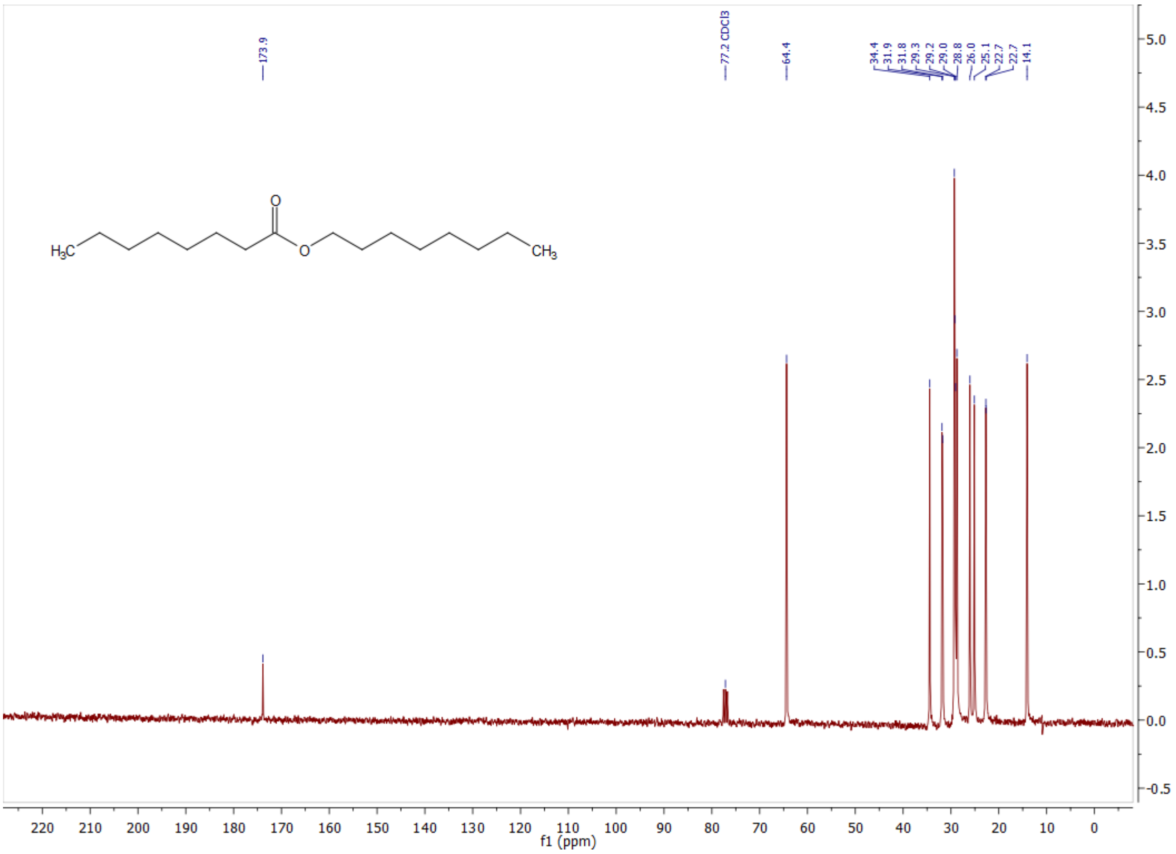

Cyclopentyl octanoate (2g)

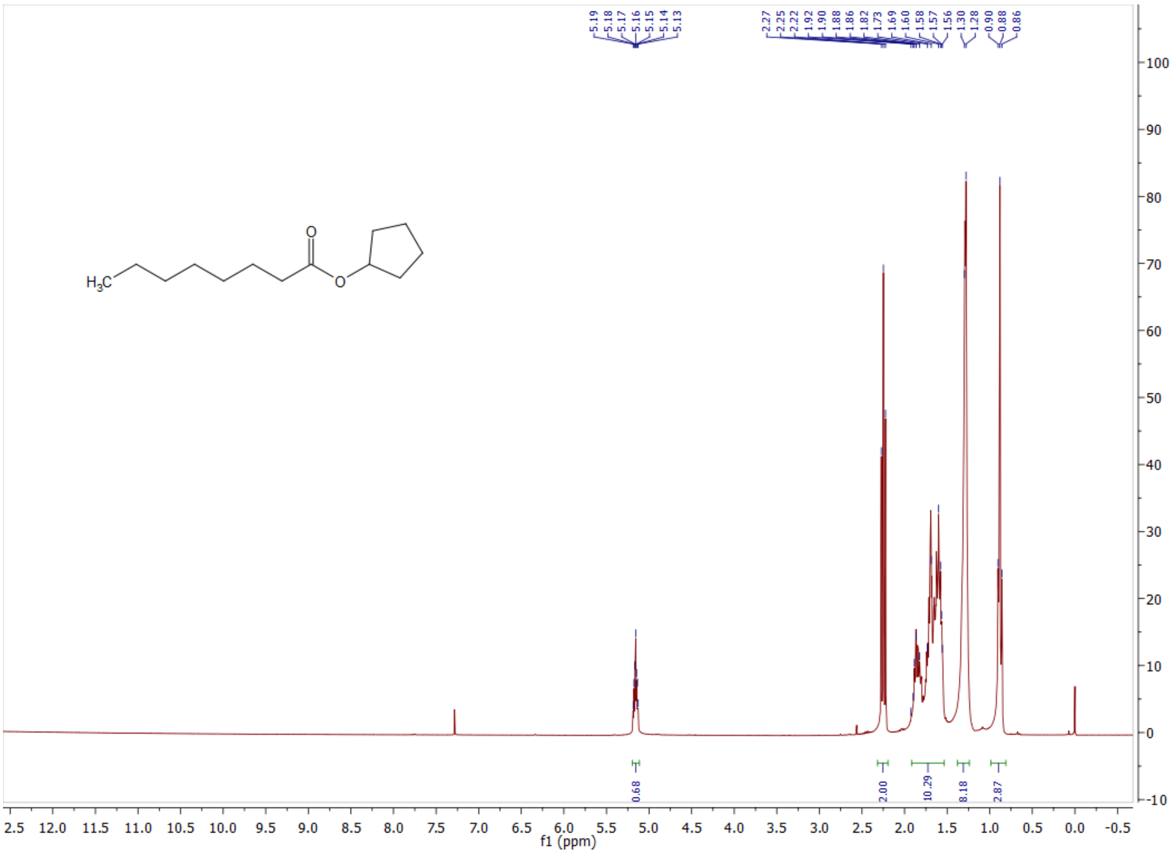

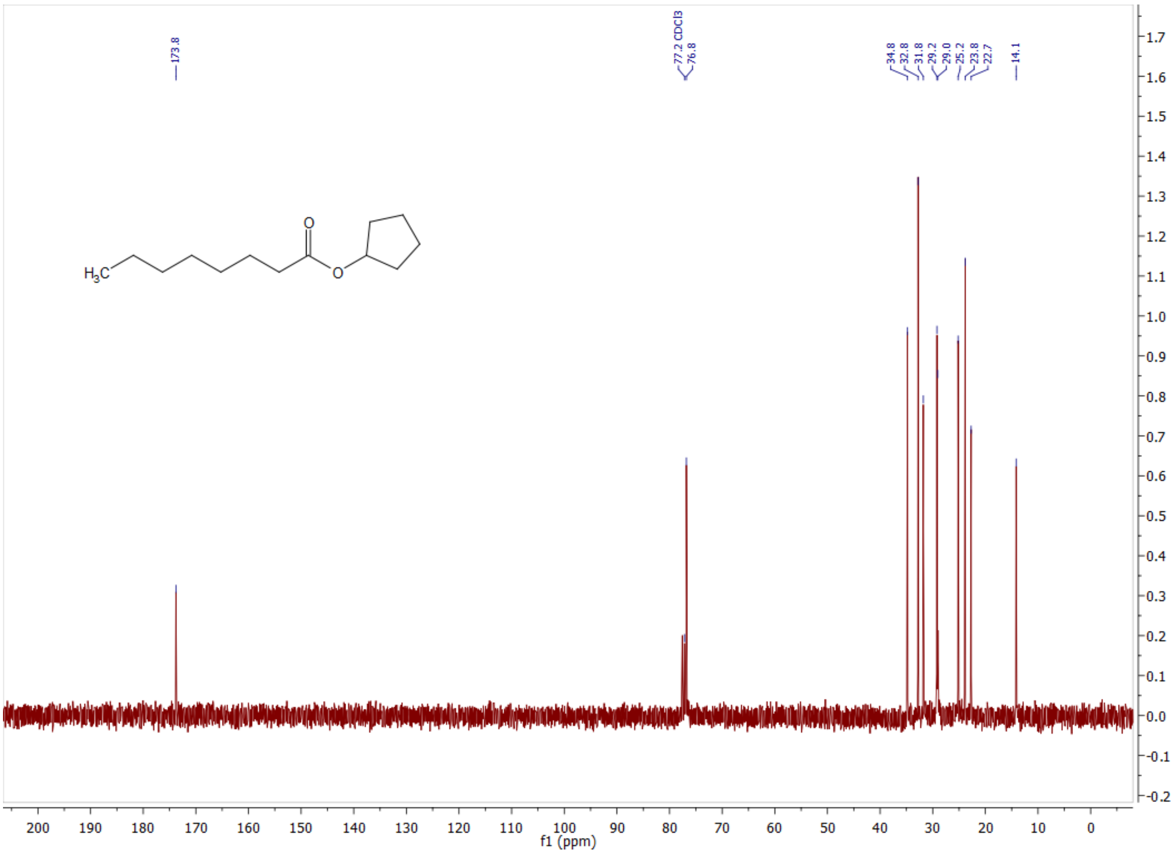

Succinimide

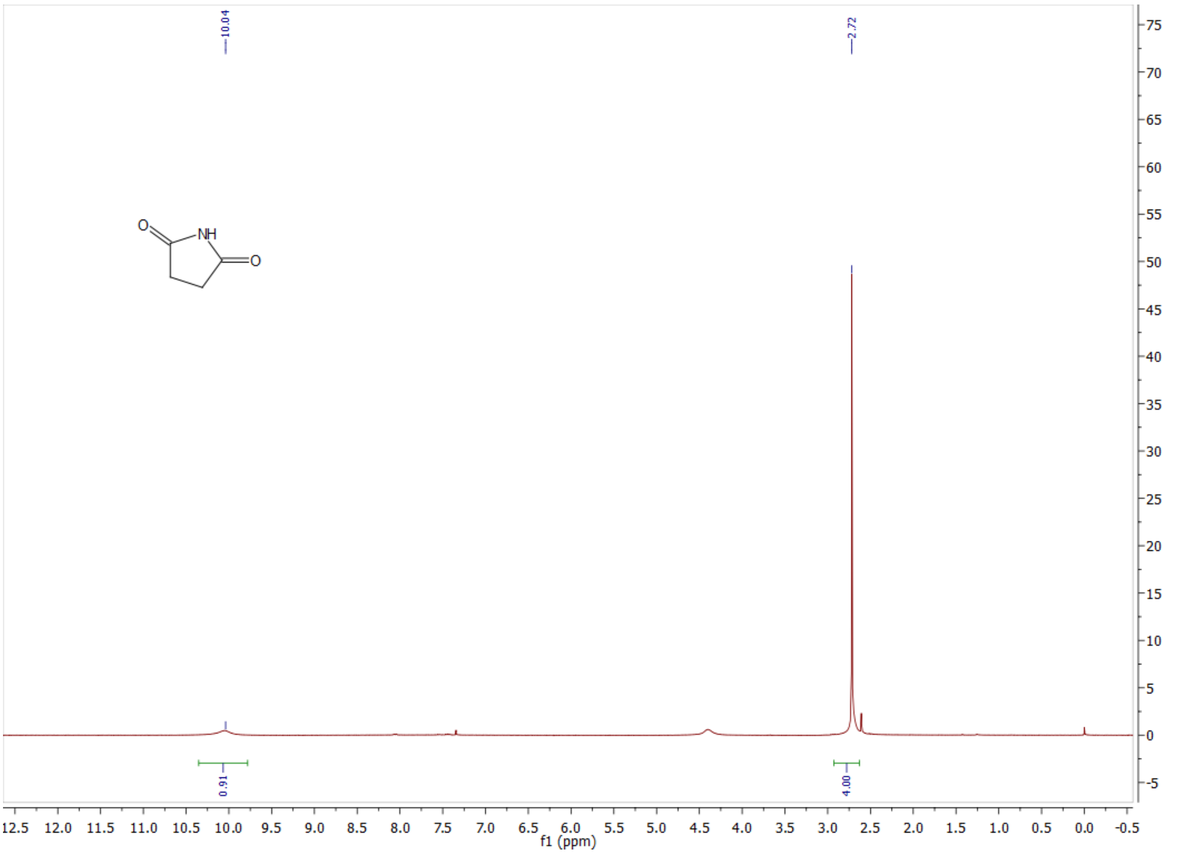

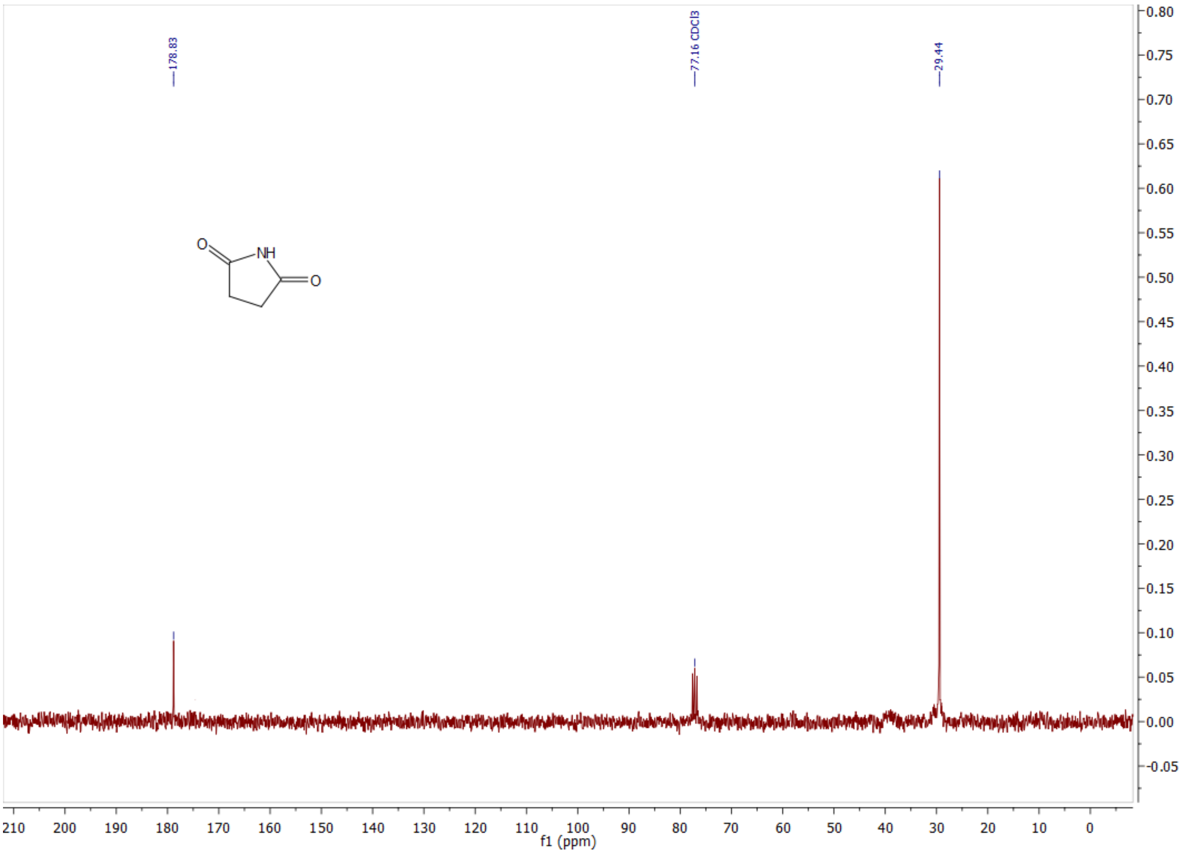

Supplement: Supplementary file 1 [file molecules-23-02235-s001.pdf]
